# FedMamba-ChebKAN: Federated Mamba with Chebyshev-KAN for heterogeneous IIoT fault detection

**DOI:** 10.1371/journal.pone.0354012

**Published:** 2026-11-11

**Authors:** Issa M. Alsmadi, Mohammed Tawfik, Islam S. Fathi

**Affiliations:** 1 Department of Computer Science, Faculty of Information Technology, Ajloun National University, Ajloun, Jordan; 2 Faculty of Computer and Information Technology, Sana’a University, Sana’a, Yemen; 3 Department of Information Systems, Al Alson Higher Institute, Cairo, Egypt; Maulana Abul Kalam Azad University of Technology West Bengal, INDIA

## Abstract

Fault detection in edge-based Industrial Internet of Things (IIoT) systems requires models that generalise across heterogeneous devices while respecting data-governance and bandwidth constraints. Federated learning (FL) avoids raw-data exchange, but standard optimisers degrade under strong statistical heterogeneity, and Kolmogorov–Arnold Network (KAN) heads parameterised by client-adaptive B-splines add a grid-misalignment problem that weight averaging cannot resolve. We present FedMamba-ChebKAN, which couples a Mamba selective state-space encoder, with 𝒪(L) cost over long sensor sequences, to a *Chebyshev-KAN* head whose polynomial activations share one client-invariant basis, so that coefficient averaging is well posed without re-interpolation. Its aggregation rule, SpectralFedAvg, truncates each KAN edge at a tail-energy threshold and averages coefficients at a consensus degree on the shared basis; a variant, SpectralFedAvg-BN, keeps BatchNorm on the clients. A proposition bounds the error of the aggregated edge function by a truncation term plus a client-heterogeneity term. We evaluate on CWRU, Paderborn and MIMII (12 clients each) and NASA C-MAPSS (20 clients), with splits made on recordings or engines before windowing, all federated baselines on the same architecture, eight seeds per configuration, and exact Wilcoxon tests with Benjamini–Hochberg correction. SpectralFedAvg-BN reaches 98.0±0.5% accuracy on CWRU, 12.3 points above FedAvg (*q* = 0.023) and level with FedBN, and the lowest RMSE among the federated methods on C-MAPSS (20.29±0.51 cycles, against 20.89 for FedAvg, *q* = 0.039). On Paderborn (98.0%) and MIMII (AUROC 0.805), the federated methods that share the architecture lie within about one standard deviation of each other. With DP-SGD on every client and an encoder pretrained on public data, the model keeps 89.5% CWRU accuracy at example-level $\left(\varepsilon \;\approx \;8,\delta \;=\;10^{-5}\right)$-DP and 92.4% at ε≈19. Code, client partitions and per-seed results are public.

## Introduction

Edge-based Industrial Internet of Things (IIoT) systems continuously collect high-rate sensor telemetry: vibration and current signals from rotating machinery, acoustic emissions from industrial equipment, and multivariate degradation streams from complex assets. Early fault detection in these environments directly reduces unplanned downtime, prevents cascading failures, and optimises predictive maintenance scheduling [1,2]. Despite the success of deep learning, centralised analytics is impractical at scale: raw sensor transmission is costly, latency constraints favour local decision-making, and operational data are increasingly restricted by privacy regulations and proprietary contracts that prohibit data transfer even when bandwidth is available [3,4].

Federated learning (FL) resolves both constraints by training a global model collaboratively across distributed edge clients while keeping raw data local and sharing only model updates [5]. In industrial fault detection, however, statistical heterogeneity is extreme: different devices observe distinct operating conditions, wear states, and fault exposure rates, producing highly imbalanced non-IID local datasets. Zhou *et al.* [6] demonstrate that FedAvg accuracy on the CWRU bearing dataset collapses to 60.47% under 23:1 class imbalance—a failure mode that disqualifies standard FL for safety-critical monitoring.

Three architectural opportunities motivate this work. **First, the backbone.** Existing federated fault-detection studies rely on 1D-CNN or LSTM backbones [1,6,7]: CNNs impose fixed receptive fields that miss long-range cyclostationarity, LSTMs incur memory cost that constrains sequence length, and Transformer attention scales quadratically, which is impractical for the 64 kHz Paderborn recordings [8]. Mamba [9], a selective state-space model whose state matrices are functions of the input, achieves 𝒪(L) cost while matching Transformer accuracy on long-range tasks. The federated case is already established in adjacent domains: FedMamba-IoMT [10] federates stacked selective-SSM blocks for medical-IoT intrusion detection with DP-SGD, Rényi accounting, and Byzantine-resilient aggregation, confirming that an SSM backbone trains stably under FL. Centralised work has separately begun pairing Mamba with KAN heads [11,12]. What is missing, and what we address, is the combination: a federated Mamba encoder whose KAN head is aggregated by a rule that accounts for the head’s basis parameterisation rather than treating it as ordinary weights. **Second, the head.** MLP heads apply fixed node activations and offer limited interpretability, a shortcoming when diagnoses must be auditable by maintenance engineers. KANs [13] instead learn a univariate function on each edge, giving per-edge interpretability and strong fault-classification accuracy [14]. Their original B-spline parameterisation, however, creates an aggregation problem specific to FL: once each client adapts its knot vector to local data, averaging misaligned grids degrades the approximation. Two routes exist—re-interpolate onto a consensus grid, or adopt a basis identical on every client. We take the latter: the Chebyshev basis $\{T_{n}(t){\}}_{n=0}^{N}$ is fixed on [−1,1] for all clients, making coefficient averaging well posed by construction. The closest prior work, CG-FKAN [15], sparsifies grids for communication efficiency but performs no explicit alignment. **Third, privacy.** FL prevents raw data transfer, yet updates still leak through gradient inversion and membership inference [16,17], and industrial deployments increasingly require auditable guarantees rather than mere data locality; the surrounding cloud infrastructure is itself an active attack surface addressed by complementary network-level defences [18,19]. That formal DP and industrial utility can be compatible is shown by Putra *et al.* [20] at 94.00% bearing accuracy under (ε,δ)-DP. We give the framework an example-level guarantee by running DP-SGD inside every client, so that the guarantee also holds against the coordinator, and we measure its accuracy cost.

To our knowledge, no published study combines (a) a Mamba/SSM encoder, (b) a Chebyshev-KAN head with a basis-invariant federated aggregation rule, and (c) evaluation on CWRU, Paderborn, MIMII, and C-MAPSS under non-IID conditions with K≥12 clients. The closest precedents each cover at most two of these three elements: VibrMamba [11] pairs Mamba with KAN but is centralised; FedMamba [21] federates Mamba sequence models with FedProx for multimodal intrusion detection, with a conventional classification head; F-KANs [22] and CG-FKAN [15] apply KAN in FL on non-industrial benchmarks (UCI Iris, communication channels) without Mamba; and FedSNGP [1] performs FL on CWRU/Paderborn with SNGP-based clustering, without Mamba or KAN. Existing federated work on C-MAPSS [8,23,24] reports RMSE in the range 14–17 on subsets of FD001–FD004 under standard FedAvg/FedProx with LSTM or transformer backbones; we use these as direct comparators. For acoustic anomaly detection, existing federated work [25] explores embedding sharing on DCASE-derived data but does not, to our knowledge, report results on the standard MIMII ±6/0/−6 dB protocol. The four-dataset benchmark with K≥12 clients presented here addresses this gap.

The contributions of this paper are: (i) FedMamba-ChebKAN, a federated framework integrating a Mamba selective SSM encoder (𝒪(L) complexity) with a Chebyshev-KAN head supporting supervised fault classification (CWRU, Paderborn) and unsupervised anomaly/RUL detection (MIMII, C-MAPSS); (ii) SpectralFedAvg, an FL aggregation rule that exploits the client-invariant Chebyshev basis through adaptive coefficient truncation and consensus-degree averaging, with Proposition 1 bounding the error of the aggregated edge function by a truncation term controlled by the tail-energy tolerance and a client-heterogeneity term, together with a client-local normalisation variant (SpectralFedAvg-BN) for feature-shift heterogeneity; we differentiate this rule from KAN-FL grid compression [15] and B-spline re-interpolation; (iii) an example-level differentially private variant in which every client runs DP-SGD on a model whose encoder is pretrained on public data, so that the guarantee holds against the coordinator and covers the transmitted truncation degrees, together with its measured privacy–utility trade-off; (iv) a reproducible four-dataset benchmark with K≥12 clients, in which splits are made on recordings or engines before windowing, all federated baselines share the architecture of the proposed method, every configuration is run with eight seeds, and the code, client partitions and per-seed results are public; it includes a federated evaluation on the MIMII ±6/0/−6 dB setting and on the official C-MAPSS test engines of all four subsets. The Related work section surveys related work; the Methods section presents the architecture, datasets, SpectralFedAvg, and the evaluation protocol; the Results section reports results, a component analysis, and statistical tests; the Discussion section interprets findings and discusses limitations; the Conclusion section concludes.

## Related work

### Federated learning under heterogeneity and for industrial fault detection

McMahan *et al.* [5] established FedAvg as the canonical FL training loop, while Konečný *et al.* [26] formalised the on-device setting characterised by massively distributed, unevenly distributed, and typically non-representative local datasets, identifying communication as the primary bottleneck. Li *et al.* [3] extended FedAvg to heterogeneous networks via a proximal regulariser, improving stability under variable participation and non-IID data. Karimireddy *et al.* [4] addressed client drift via SCAFFOLD using control variates, and the empirical study of Li *et al.* [27] confirms that no single FL algorithm dominates across all heterogeneity types and that FedAvg/FedProx become unstable under label-quantity skew. We adopt the four-algorithm comparison (Local, FedAvg, FedProx, SCAFFOLD), augmented by FedBN [28] as a personalisation baseline, as the scaffold for SpectralFedAvg.

For industrial fault diagnosis, FA-FedAvg [7] reports a 52.5% reduction in training iterations and 8.6% average accuracy gain on CWRU, while Zhou *et al.* [6] systematically analyse intra-client class imbalance, showing that FedAvg accuracy collapses to 60.47% under 23:1 imbalance on CWRU. FedSNGP [1] evaluates Gaussian-process uncertainty-guided clustering on CWRU and Paderborn, achieving 95.56% on CWRU and 99.81% on PU under their unknown-class scenario. Recent federated condition-monitoring work [8] applies FedAvg/FedProx/SCAFFOLD to industrial cyber-physical sensor streams, but targets a single sensing modality. Robustness, personalisation, and privacy are increasingly entangled in this setting: Zhang *et al.* [29] design a personalised FL fault-diagnosis method that remains accurate when a subset of clients is corrupted by network attacks, and Liu *et al.* [30] couple federated transfer learning with client-side privacy protection to share bearing-diagnosis knowledge across machines without exposing raw signals—yet both rely on conventional weight averaging and leave open how structured, basis-parameterised classification heads should be aggregated. For RUL specifically, Kamei and Taghipour [23] and Arunan *et al.* [24] establish federated baselines on C-MAPSS using transformer and matched-feature-extraction aggregation, reporting RMSE in the range 14–17 on FD001–FD004 under FedAvg/FedProx; we use these as direct comparators. For acoustic anomaly detection, Dohi and Kawaguchi [25] explore embedding sharing for distributed anomalous-sound detection on DCASE 2024 data but do not benchmark on the standard MIMII protocol. Compared to these works, FedMamba-ChebKAN simultaneously (i) evaluates across all four canonical IIoT benchmarks under a unified non-IID protocol, (ii) pairs a Mamba SSM backbone with a structured KAN head in a federated setting—federated Mamba itself having been demonstrated for IoMT intrusion detection [10], though with a conventional classifier and standard weight averaging—and (iii) explicitly eliminates the basis-misalignment problem in KAN-equipped FL via a Chebyshev parameterisation.

### Mamba state-space models and Kolmogorov–Arnold networks

Gu and Dao [9] introduced Mamba, a selective SSM whose structured state matrices are computed as functions of the input, enabling input-dependent filtering at 𝒪(L) computational and memory cost; Mamba-3B matches Transformer-based models twice its size while achieving 5× higher inference throughput. Its linear scaling has driven rapid adoption for long-sequence forecasting and diagnosis: Ahamed and Cheng [31] introduce TimeMachine, a quadruple-Mamba architecture that captures multi-scale dependencies for long-term multivariate forecasting at a small memory footprint, while Deng *et al.* [32] fuse multi-domain representations in an improved RSMamba that exceeds 99% accuracy for wheelset bearing fault diagnosis under composite operating conditions. Yi *et al.* [11] applied Mamba to rotating-machinery vibration in VibrMamba, replacing the standard linear projection head with a KAN component and reporting 99.77%, 99.84%, and 99.95% diagnostic accuracy on CWRU, XJTU, and HIT under centralised training. Wang *et al.* [33] extended Mamba to aero-engine inter-shaft bearing diagnosis via bidirectional SSM blocks with modal decomposition, while Sellam *et al.* [34] combined Mamba with Transformer-style association discrepancy for multivariate time-series anomaly detection. These centralised Mamba works confirm the architectural viability but provide no federated protocol. In the federated setting, Ahmad *et al.* [21] combine Mamba sequence models with FedProx and modality-aware aggregation for multimodal intrusion detection in heterogeneous IoT systems, reporting stable convergence under non-IID data and adversarial training; their task, modalities, and data differ from ours, so their numbers are not comparable with the present benchmarks, and their head is a conventional classifier rather than a basis-parameterised KAN. The design tension they leave open is not unique to SSMs: in financial time-series forecasting, Kundavaram *et al.* [35] show that hybrids combining econometric predictors with neural residual learners gain most when the ensemble weights are re-estimated from rolling error statistics rather than fixed a priori, because regime shifts otherwise degrade any static combination. The same principle motivates our per-edge adaptive truncation, where the retained spectral degree is re-derived every round from the local coefficient energy instead of being fixed globally.

KANs [13] parameterise each inter-node connection with a learnable univariate function $\varphi_{ij}\;:\;\mathbb{R}\;\to \;\mathbb{R}$ rather than applying a fixed activation to a learned scalar combination, enabling per-edge interpretability. The original work used B-splines, but the choice of basis is incidental to the Kolmogorov–Arnold representation: Chebyshev polynomials, Fourier series, and radial basis functions all yield valid KAN parameterisations with distinct numerical properties [36,37]. SS *et al.* [38] instantiate this idea as a Chebyshev-polynomial KAN, showing that a fixed orthogonal Chebyshev basis attains more parameter-efficient and numerically stable nonlinear approximation than B-splines on standard benchmarks; we adopt this parameterisation as our edge activation and show, in addition, that its client-invariance is precisely the property that renders federated coefficient averaging well posed. The Chebyshev basis is particularly attractive in federated settings because it is fixed on [−1,1] and identical on every client, eliminating the grid-alignment problem at the source. The contrast with the B-spline route is instructive in a setting we have examined directly: MambaResp-KAN [12] pairs a bidirectional Mamba encoder with a KAN classifier whose learnable B-spline activations deliver both parameter efficiency and per-edge interpretability, reaching 99.6% and 99.4% on two respiratory-sound benchmarks. That architecture is centralised, and the adaptive B-spline grid that makes it effective there is precisely what becomes problematic once the same head must be averaged across clients with differing local distributions—each client’s knots drift toward its own data, and the averaged coefficients no longer index a common basis. The Chebyshev parameterisation trades the B-spline’s local adaptivity for a global basis that every client shares, which is the property federated averaging actually requires. Dong *et al.* [39] evaluated KANs on 128 UCR time-series benchmarks, finding competitive accuracy with lower Lipschitz constants. For fault diagnosis, Rigas *et al.* [14] report essentially perfect F1 on CWRU bearing severity classification, and Cabral *et al.* [40] applied KANs to power-transformer dissolved-gas analysis with imbalanced data, achieving Hamming loss 0.0323. The intersection of FL and KAN is nascent: F-KANs [22] demonstrate 100% federated accuracy on UCI Iris from round 7; Sasse and Miceli de Farias [41] evaluate federated Spline-KANs on pathologically non-IID MNIST; and Lee *et al.* [42] benchmark FL-KAN on medical imaging. The closest prior work to ours is CG-FKAN [15], which addresses the per-round KAN communication overhead in FL by sparsifying coefficient vectors and transmitting only the most-essential entries under a communication budget, with a derived approximation-error upper bound. CG-FKAN and SpectralFedAvg target related but distinct problems: CG-FKAN *compresses* the B-spline state under bandwidth constraints, treating client grids as fixed; SpectralFedAvg *eliminates* client grid divergence at the source by using a basis-invariant Chebyshev parameterisation and aggregating via adaptive truncation. The two approaches are complementary, and a combined truncation-plus-sparsification scheme is a natural direction for future work. None of the FL-KAN works above has been evaluated on industrial fault-detection data, and none pairs a Chebyshev-KAN head with a Mamba SSM encoder.

### Privacy-preserving FL and positioning

Bonawitz *et al.* [16] proposed practical secure aggregation, ensuring the server observes only the sum of client updates while remaining robust to client dropout. Geyer *et al.* [17] formalised client-level differential privacy for FL, showing that per-client DP can be maintained at modest accuracy cost via calibrated Gaussian noise. Gu *et al.* [43] operationalise this for the industrial setting with FL2DP, injecting calibrated differential-privacy noise into federated updates for artificial-IoT services and characterising the resulting privacy–utility trade-off. For IIoT, Putra *et al.* [20] combine proof-of-authority blockchain with Gaussian DP aggregation for bearing fault classification at 94.00% accuracy under (ε,δ)-DP, while Mahmud *et al.* [44] achieve 99.94% intrusion-detection accuracy on UNSW-NB15 with FedOpt + DP. Noise injection is not the only route to confidentiality: Bandhela *et al.* [45] preserve analytical utility by mining over homomorphically encrypted records under a Brakerski/Fan–Vercauteren scheme, and Pallela and Banerjee [46] pair homomorphic authentication with federated training across several providers so that models are fitted without relocating data into a single administrative domain. Encryption-based designs avoid the accuracy cost of injected noise but do not by themselves bound what an aggregated update discloses, whereas DP supplies precisely that bound; the two are best read as complementary layers. A third layer sits below both, at the point of device admission: before a client may contribute an update at all, it must be authenticated, and doing so without leaking device identity motivates zero-knowledge and post-quantum constructions such as the blockchain-anchored ZKP authentication framework of Tawfik *et al.* [47]. A fully specified IIoT deployment therefore composes admission control, transport confidentiality, for which AES with end-to-end encryption remains the standard mitigation in multi-tenant cloud pipelines [48], and DP; we address the last at the level of individual training examples and take the first two as given. Unlike perturbation of the aggregate at the coordinator, the DP-SGD variant evaluated here adds the noise on the clients, so its guarantee does not rely on a trusted coordinator.

#### Federated time-series modelling.

A parallel line of work applies FL directly to sequence tasks rather than to fault diagnosis specifically. FeD-TST [49] federates a temporal sparse transformer for QoS forecasting across IoT network configurations, keeping per-configuration data local for confidentiality; FedST [50] builds a secure federated shapelet-transformation protocol for time-series classification with proven security and multi-order-of-magnitude speedups over the naive protocol. Both establish that sequence models can be federated without exposing raw traces, but neither addresses basis-parameterised heads, and neither targets industrial fault signatures. We also note a naming ambiguity worth flagging, since it recurs in reviews of this literature: FEDformer [51] is often assumed to be a federated method, but the prefix abbreviates *Frequency Enhanced Decomposed*, and the method is centralised. It is nonetheless the closest conceptual neighbour to SpectralFedAvg outside FL, because it likewise exploits the sparsity of time-series signals in a fixed frequency basis; the distinction is that FEDformer uses the frequency domain to *compress attention within one model*, whereas SpectralFedAvg uses a fixed orthogonal basis to make *coefficient averaging across clients* well posed, which is a federated-aggregation problem with no centralised analogue.

Table 1 positions FedMamba-ChebKAN relative to the closest related works across the five distinguishing dimensions of this study.

**Table 1:** Comparison of FedMamba-ChebKAN with the closest related works.

Method	FL	Mamba	KAN	Multi-dataset	Privacy
FedSNGP [1]	✓	–	–	Partial	–
FA-FedAvg [7]	✓	–	–	–	–
Zhou *et al.* [6]	✓	–	–	–	–
VibrMamba [11]	–	✓	✓	–	–
RSMamba [32]	–	✓	–	–	–
ChebyKAN [38]	–	–	✓	–	–
F-KANs [22]	✓	–	✓	–	–
CG-FKAN [15]	✓	–	✓	–	–
Putra *et al.* [20]	✓	–	–	–	✓
FL2DP [43]	✓	–	–	–	✓
Kamei & Taghipour [23]	✓	–	–	Partial	–
FedMamba-IoMT [10]	✓	✓	–	Partial	✓
FedMamba [21]	✓	✓	–	Partial	–
FEDformer [51]^‡^	–	–	–	Partial	–
FeD-TST [49]	✓	–	–	–	–
FedST [50]	✓	–	–	Partial	✓
**FedMamba-ChebKAN (Ours)**	✓	✓	✓	✓	✓^§^

‡FEDformer is included because it is frequently grouped with federated time-series methods, but despite its name it is *not* a federated method: the “FED” prefix denotes *Frequency Enhanced Decomposed*. It is a centralised transformer, and we list it here for its frequency-domain decomposition, which is the property most closely related to SpectralFedAvg. ^§^Example-level (ε,δ)-DP through DP-SGD on every client (private variant).

## Methods

FedMamba-ChebKAN integrates four tightly coupled design decisions: (i) a three-tier edge–gateway–cloud hierarchy for hierarchical FL; (ii) a Mamba SSM encoder at linear complexity; (iii) a Chebyshev-KAN head with learnable polynomial activations on a client-invariant basis; and (iv) SpectralFedAvg, which aggregates Chebyshev coefficients through adaptive truncation and consensus-degree averaging, with an optional client-local normalisation variant (SpectralFedAvg-BN) and an example-level differentially private variant. Fig 1 summarises the client data, the local model, one federated round, and three component details.

**Fig 1:**
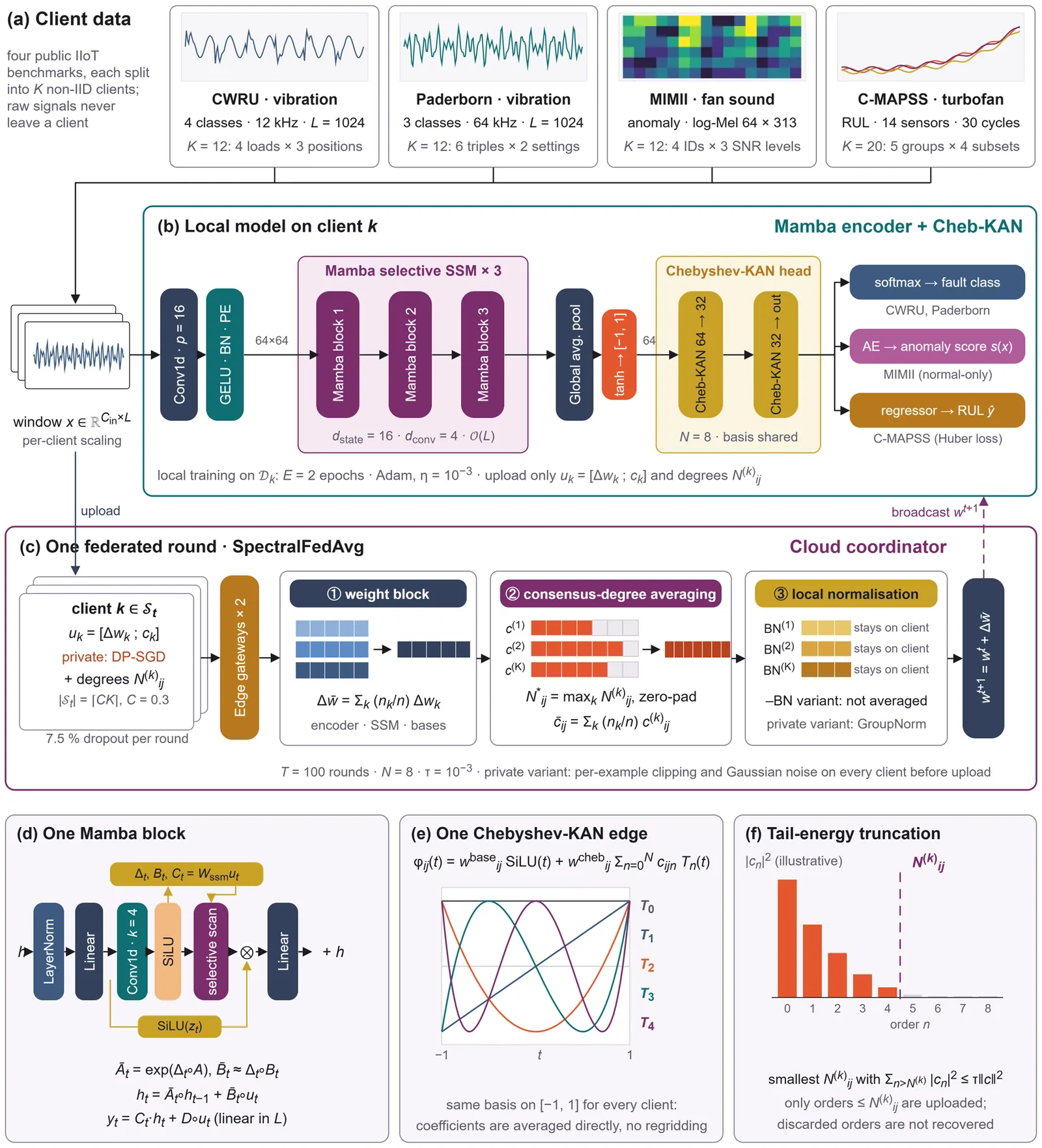
Overview of FedMamba-ChebKAN. (a) Client data: the four public IIoT benchmarks with their task, sampling rate, input size and client definition. CWRU: four classes, 12 kHz, windows of 1024 samples, 12 clients formed by four motor loads and three sensor positions. Paderborn: three classes, 64 kHz, 12 clients formed by six bearing triples and two operating settings. MIMII fan: anomaly detection on 64×313 log-Mel spectrograms, 12 clients formed by four machine IDs and three SNR levels. C-MAPSS: RUL prediction from 14 sensors over 30 cycles, 20 clients formed by five engine groups in each of the four subsets. Raw signals never leave a client. (b) Local model on client *k*: a Conv1d patch embedding (patch length 16 for the vibration windows) with GELU, BatchNorm and positional encoding; three Mamba selective state-space blocks (*d*_state_ = 16, *d*_conv_ = 4, cost linear in the sequence length); global average pooling; a tanh map to [−1,1]; and a two-layer Chebyshev-KAN head (64→32→ output, degree *N* = 8, basis shared by all clients). Task heads are a softmax classifier (CWRU, Paderborn), an autoencoder anomaly score trained on normal clips (MIMII) and a RUL regressor trained with the Huber loss (C-MAPSS). Each client trains for *E* = 2 epochs with Adam and uploads only the update $u_{k}\;=\;[\Delta w_{k};c_{k}]$ and the truncation degrees $N_{ij}^{\left(k\right)}$. (c) One SpectralFedAvg round at the cloud coordinator: a fraction *C* = 0.3 of the clients is sampled per round (7.5 % dropout) and their uploads pass through the edge gateways. Step 1 averages the non-Chebyshev weights with weights $n_{k}/n$; step 2 averages each KAN edge at the consensus degree $N_{ij}^{*}\;=\;\max_{k}N_{ij}^{\left(k\right)}$ after zero-padding; step 3 keeps BatchNorm on the clients in SpectralFedAvg-BN (GroupNorm in the private variant). The new global model $w^{t+1}$ is broadcast for the next of *T* = 100 rounds; in the private variant every client runs DP-SGD, with per-example clipping and Gaussian noise, before its upload. (d) One Mamba block: LayerNorm, input projection, causal depthwise convolution (*k* = 4), SiLU, input-dependent $\Delta_{t}$, $B_{t}$ and $C_{t}$, selective scan, SiLU gate, output projection and residual connection, with the discretised recurrence below. (e) One Chebyshev-KAN edge $\varphi_{ij}(t)\;=\;w_{ij}^{base}SiLU(t)+w_{ij}^{cheb}\sum_{n=0}^{N}c_{ijn}T_{n}(t)$ and the first five Chebyshev polynomials on [−1,1]; the basis is identical on every client, so coefficients are averaged directly without regridding. (f) Tail-energy truncation on an illustrative coefficient spectrum: each client keeps the smallest degree $N_{ij}^{\left(k\right)}$ whose discarded coefficients carry at most a fraction τ of the coefficient energy, and only the orders up to $N_{ij}^{\left(k\right)}$ are uploaded.

### System architecture and benchmark datasets

The three-tier hierarchy comprises: *Tier 1 – IoT clients*, where client k∈{1,...,K} holds private data ${𝒟}_{k}\;=\;\{(x_{i},y_{i}){\}}_{i=1}^{n_{k}}$ and after *E* local training epochs transmits only the update $u_{k}\;=\;[\Delta w_{k};c_{k}]$, formed from the weight update $\Delta w_{k}\;=\;w_{k}^{t+1}\;-\;w^{t}$ and the truncated Chebyshev coefficients $c_{k}$, together with the per-edge truncation degrees $N_{ij}^{\left(k\right)}$; raw sensor data never leave the device; *Tier 2 – edge gateways*, which buffer stragglers and forward client updates (in the simulation reported here they forward updates unchanged, and intermittent participation is modelled by client dropout); and *Tier 3 – cloud coordinator*, which maintains the global model $w^{t}$, orchestrates rounds, runs SpectralFedAvg, and broadcasts $w^{t+1}$. In a production fleet, assigning clients to gateways would be a load-balancing decision in its own right, for which swarm-intelligence schedulers that adapt allocations to live resource and latency telemetry offer a natural mechanism [52].

We evaluate on four canonical IIoT benchmarks (Table 2) covering distinct sensing modalities and task formulations. Client counts are K≥12 per dataset, reflecting realistic IIoT fleet sizes. Every configuration of the main comparison is run with *R* = 8 independent seeds (three for the privacy study); partitions are fixed and the seeds vary initialisation, client sampling and mini-batch order.

**Table 2:** Benchmark datasets and federated partitions. Every split is made on recordings, recording segments or engines before windowing; S2 File lists the full assignment.

Dataset	Task	Clients (*K*)	Training data per client	Test set
CWRU [53]	4-class	4 loads × 3 sensor positions (12)	195–260 windows	1760 windows
Paderborn [54]	3-class	2 settings × 6 bearing triples (12)	219 windows	1440 windows
MIMII [55]	Anomaly	4 machine IDs × 3 SNR (12)	215 normal clips	2400 clips
C-MAPSS [56]	RUL regression	4 FD subsets × 5 engine groups (20)	900 windows	707 engines

Every split below is made on whole recordings, recording segments or engines *before* windowing, so no window shares samples with a window in another split, and normalisation statistics are fitted on each client’s training data only. **CWRU** [53]: 12 kHz recordings of the drive-end test rig with four classes (normal; ball, inner-race and outer-race at 6:00, each pooling the 7-, 14- and 21-mil faults). The 12 clients are the 4 motor loads (0–3 hp) × 3 accelerometer positions (drive end, fan end, base). Each recording is cut into contiguous 60/20/20 % segments for training, validation and testing before windowing (*L* = 1024 samples, 50 % overlap only inside a segment), and each client keeps 65 training and 40 validation and 40 test windows per class. The normal-baseline recordings carry no base-accelerometer channel, so the three base-position clients hold no normal windows, a natural form of label skew. **Paderborn** [54]: 64 kHz vibration of six bearings with real damage or none (healthy K001 and K002; outer race KA04 and KA15; inner race KI04 and KI14) under two operating settings (N15_M07_F10 and N15_M01_F10). Each of the 12 clients holds one healthy, one outer-race and one inner-race bearing under one setting, using the six bearing combinations per setting that exclude the two complementary ones, so clients differ in devices and operating condition while sharing the three classes. The 20 recordings of each bearing and setting are dealt to its three clients without overlap and split 4/1/1 by recording before windowing (*L* = 1024); each client keeps 219 training windows (73 per class) and 120 validation and 120 test windows. **MIMII** [55]: fan recordings (channel 0, 16 kHz, 10 s) converted to 64×313 log-Mel spectrograms (STFT 1024, hop 512) [57]. The 12 clients are machine IDs 00, 02, 04 and 06 × SNR levels + 6, 0 and −6 dB [55,58]. Clips are independent recordings and are split by clip: each client trains on 215 normal clips, validates on 40 normal clips, and is tested on 100 unseen normal and 100 abnormal clips. **C-MAPSS** [56]: 14 informative sensors, windows of 30 cycles, and RUL target y=min(RUL,125). The 20 clients are 5 groups of training engines per FD subset; within a client one engine in ten is held out for validation, and 900 training windows are kept. Sensors are standardised per operating condition (six conditions in FD002 and FD004) with statistics from the client’s training engines. The test set is the official test set of each subset (707 engines in total), scored on the last window of each engine against the official RUL, i.e., one prediction per engine as in the PHM08 protocol, with RMSE and the asymmetric score


$$\begin{array}{c} Score=\sum_{i}\left\{ \begin{array}{cc} e^{-d_{i}/13}-1, & d_{i}<0\text{ (early)},\\ e^{d_{i}/10}-1, & d_{i}\geq 0\text{ (late)}, \end{array}\right. \end{array}$$ (1)

where $d_{i}\;=\;{\hat{y}}_{i}-y_{i}$ for engine *i* [59,60]. Across all datasets, ⌈CK⌉ clients with *C* = 0.3 are sampled per round, and each sampled client drops out with probability 0.075 to simulate intermittent participation. All methods share the same partitions and seeds.

### FedMamba-ChebKAN model architecture

The local model (Fig 1b) comprises three stages.

#### Patch embedding.

Given $X\;\in \;{\mathbb{R}}^{C_{in}\times L}$, $𝐄\;=\;BN(GELU(Conv1d(X;W_{emb})))\;\in \;{\mathbb{R}}^{d_{m}\times L_{p}}$ with $L_{p}\;=\;\lfloor L/p\rfloor$. Learnable positional encodings $𝐏\;\in \;{\mathbb{R}}^{L_{p}\times d_{m}}$ are added: ${𝐇}^{\left(l\right)}\;=\;{𝐄}^{⊤}+𝐏\;\in \;{\mathbb{R}}^{L_{p}\times d_{m}}$. The patch length is *p* = 16 for the 1024-sample vibration windows ($L_{p}\;=\;64$ tokens), *p* = 8 for the MIMII spectrograms (*C*_in_ = 64 mel bands, $L_{p}\;=\;39$) and *p* = 1 for C-MAPSS (*C*_in_ = 14, $L_{p}\;=\;30$).

#### Mamba selective SSM blocks.

Each of *N*_layers_ stacked blocks [9] processes **H**^(*l*)^ via input projection $[\tilde{x},z]\;=\;{𝐇}^{\left(l\right)}{𝐖}_{in}\;\in \;{\mathbb{R}}^{L_{p}\times 2d_{inner}}$ (where $d_{inner}\;=\;E\;d_{m}$), causal depthwise convolution of kernel *d*_conv_ on $\tilde{x}$ followed by SiLU yielding $u\;\in \;{\mathbb{R}}^{L_{p}\times d_{inner}}$, and the selective SSM mechanism


$$\begin{array}{cc} \left[{\hat{\Delta }}_{t},B_{t},C_{t}\right] & ={𝐖}_{ssm}u_{t}\in {\mathbb{R}}^{1+2d_{state}}, \end{array}$$ (2)

$$\begin{array}{cc} \Delta_{t} & =softplus({𝐖}_{\Delta }{\hat{\Delta }}_{t}+b_{\Delta })\in {\mathbb{R}}^{d_{inner}}, \end{array}$$ (3)

with 𝐀=−exp(log𝐀) (log-parameterised for stability). Following Mamba’s original parameterisation [9], we adopt the standard approximation ${\bar{𝐁}}_{t}\;\approx \;\Delta_{t}\;\circ \;B_{t}$ in place of the exact ZOH formula:


$$\begin{array}{c} {\bar{𝐀}}_{t}=\exp(\Delta_{t}\circ 𝐀),\;{\bar{𝐁}}_{t}\approx \Delta_{t}\circ B_{t}. \end{array}$$ (4)

The linear recurrence is


$$\begin{array}{c} h_{t}={\bar{𝐀}}_{t}\circ h_{t-1}+{\bar{𝐁}}_{t}\circ u_{t},\;y_{t}=C_{t}\;\cdot \;h_{t}+𝐃\circ u_{t}. \end{array}$$ (5)

We evaluate the recurrence exactly in chunks of eight steps using cumulative log-decays; for numerical range the per-step log-decay $\Delta_{t}A$ is floored at −6, since a per-step retention below $e^{-6}\;\approx \;0.25$ % already acts as a reset. The gated output $y_{t}\;\circ \;SiLU(z_{t})$ is projected to $d_{m}$ with LayerNorm and residual wrapping each block ($d_{m}\;=\;64$, *N*_layers_ = 3, *d*_state_ = 16, *d*_conv_ = 4, expansion *E* = 2). Mean pooling over tokens produces $f\;=\;\frac{1}{L_{p}}\sum_{t}h_{t}^{\left(N_{layers}\right)}\;\in \;{\mathbb{R}}^{d_{m}}$; for C-MAPSS, whose target is defined at the last cycle of the window, the final token of the causal encoder is used instead.

#### Chebyshev-KAN head.

Each KAN layer maps ${\mathbb{R}}^{d_{n}}\;\to \;{\mathbb{R}}^{d_{n+1}}$ via $\left[{KAN}_{n}(f){]}_{j}\;=\;\sum_{i=1}^{d_{n}}\varphi_{ij}({\tilde{f}}_{i}\right)$, where ${\tilde{f}}_{i}\;=\;\tanh(f_{i})\;\in \;[-1,1]$ maps activations onto the Chebyshev domain and each edge function is a truncated Chebyshev series


$$\begin{array}{c} \varphi_{ij}(t)=w_{ij}^{base}\;SiLU(t)+w_{ij}^{cheb}\sum_{n=0}^{N}c_{ijn}\;T_{n}(t), \end{array}$$ (6)

with $T_{n}(t)\;=\;\cos(n\arccos t)$ the *n*-th Chebyshev polynomial of the first kind [36,37] and *N* the maximum polynomial degree (default *N* = 8). Coefficients are initialised with amplitude decaying as (1 + *n*)^–2^, a smoothness prior under which the tail-energy criterion below is informative from the first round. The classification head has two KAN layers (64→32→ classes). Unlike B-spline KANs, the basis is *client-invariant*: every client uses the identical orthogonal basis $\{T_{n}{\}}_{n=0}^{N}$ on [−1,1], so no per-client knot vector, no grid-update step, and no client-grid divergence ever arises. We therefore omit the grid-update equation present in the original KAN formulation. For C-MAPSS the classification head is replaced by a Chebyshev-KAN bottleneck (64→32) and a linear regressor that predicts RUL/125, trained with a Huber loss whose threshold corresponds to 13 cycles; predictions are rescaled to cycles for evaluation. For MIMII it is replaced by a Chebyshev-KAN-bottleneck autoencoder $\hat{X}\;=\;Dec({KAN}_{bn}(f))$, whose decoder maps the 32-dimensional code through a linear layer to a 64×40 map and a transposed convolution (kernel and stride 8) back to 64×313; it is trained on normal clips only, with anomaly score $s(X)\;=\;{\left\|X-\hat{X}\right\|}_{F}^{2}/(C_{in}L)$.

### SpectralFedAvg with adaptive truncation and consensus-degree averaging

#### Motivation.

The fixed Chebyshev basis eliminates the spline grid-misalignment problem of B-spline KANs by construction: all clients use identical basis functions $\{T_{n}{\}}_{n=0}^{N}$, so coefficient averaging is meaningful without re-interpolation. The federated challenge shifts from *grid alignment* to *spectral heterogeneity*: clients with smoother local target functions concentrate energy in low-order coefficients, whereas clients with sharp local fault signatures spread energy to higher orders. SpectralFedAvg exploits the rapid coefficient decay of Chebyshev expansions of smooth functions [36,37] via two steps: (i) per-edge *adaptive truncation* that compresses the spectrum to a consensus degree, and (ii) consensus-degree coefficient averaging on the shared basis.

#### Adaptive truncation.

Each client transmits, per edge (*i*,*j*), the leading $N_{ij}^{\left(k\right)}\;+\;1$ entries of its coefficient vector $c_{ij}^{\left(k\right)}\;\in \;{\mathbb{R}}^{N+1}$ together with $N_{ij}^{\left(k\right)}$, the smallest degree satisfying the local tail-energy bound


$$\begin{array}{c} \sum_{n=N_{ij}^{\left(k\right)}+1}^{N}|c_{ijn}^{\left(k\right)}{|}^{2}\;\leq \;\tau \;\cdot \;{\left\|c_{ij}^{\left(k\right)}\right\|}_{2}^{2}, \end{array}$$ (7)

with tolerance $\tau \;=\;10^{-3}$. The coordinator forms the consensus degree $N_{ij}^{*}\;=\;\max_{k}N_{ij}^{\left(k\right)}$ (preserving the most expressive client) and zero-pads shorter vectors. Zero-padding restores a common length but not the coefficients a client discarded; their effect is bounded in Proposition 1. Only the leading coefficients are uploaded, so truncation reduces the Chebyshev part of the payload; the measured degree distribution and payload are reported in the Results section.

#### Spectral averaging.

Because the basis is shared, the per-edge consensus is simply the weighted mean


$$\begin{array}{c} {\bar{c}}_{ij}\;=\;\sum_{k\in {𝒮}_{t}}\frac{n_{k}}{n}\;c_{ij}^{\left(k\right)}, \end{array}$$ (8)

with $n\;=\;\sum_{k}n_{k}$. No re-interpolation, no pseudoinverse, no Greville abscissae — one weighted-sum per edge, at per-edge cost $𝒪(K\;N^{*})$ and total per round $𝒪(K\;N_{\ell }\;d_{in}\;d_{out}\;N)$; for our defaults (*K* = 12, two KAN layers, *d*_in_ = 64, *d*_out_ = 32, *N* = 8) this is $\approx 4.9\;\times \;10^{4}$ flops per round, an order of magnitude cheaper than the B-spline re-interpolation alternative and negligible against the communication cost.

#### Client-local normalisation (SpectralFedAvg-BN).

Sensor position (CWRU), bearing unit (Paderborn) and recording condition shift the distribution of the input features between clients. SpectralFedAvg-BN therefore keeps the BatchNorm layer of the patch embedding on each client, as FedBN does [28], and aggregates all other parameters with SpectralFedAvg; each client is evaluated with its own normalisation statistics. Both variants are reported.

Algorithm 1 states the full procedure.

**Algorithm 1** SpectralFedAvg (Chebyshev-KAN federated training). SpectralFedAvg-BN skips the BatchNorm parameters in Steps 1–2 and keeps them on the clients; in the private variant, LocalTrain is DP-SGD.

**Require:** Global model $w^{t}$, *K* clients, sampling rate *C*, local epochs *E*, rounds *T*, max degree *N*, tolerance τ

**Ensure:** Trained global model $w^{T}$

 1: **for** t=1,...,T **do**

 2:  Sample ${𝒮}_{t}$ of ⌈CK⌉ clients; drop each with probability 0.075

 3:  **for** each $k\in {𝒮}_{t}$ **in parallel do**

 4:   $w_{k}^{t+1}\leftarrow LocalTrain(w^{t},{𝒟}_{k},E)$; $\Delta w_{k}\leftarrow w_{k}^{t+1}\;-\;w^{t}$

 5:   For each KAN edge, $N_{ij}^{\left(k\right)}\;\leftarrow$ smallest degree satisfying Eq. (7); keep coefficients $0..N_{ij}^{\left(k\right)}$

 6:   Upload $\Delta w_{k}$, the truncated coefficients and the degrees $\left\{N_{ij}^{\left(k\right)}\right\}$

 7:  **end for**

 8:  $n\leftarrow \sum_{k\in {𝒮}_{t}}n_{k}$; $\Delta \bar{w}\leftarrow \sum_{k}(n_{k}/n)\Delta w_{k}$ ▷ Step 1: non-Chebyshev block

 9:  **for** each KAN edge (*i*,*j*) **do**

10:   $N_{ij}^{*}\leftarrow \max_{k}N_{ij}^{\left(k\right)}$; zero-pad; ${\bar{c}}_{ij}\leftarrow \sum_{k}(n_{k}/n)\;c_{ij}^{\left(k\right)}$ ▷ Step 2, Eq. (8)

11:  **end for**

12:  $w^{t+1}\leftarrow w^{t}+\Delta \bar{w}$; update the Chebyshev state; broadcast

13: **end for**

14: **return** $w^{T}$

#### Error of the aggregated edge function.

The following proposition bounds, for one edge and one round, the error introduced by Steps 2a–2b. It separates the part that SpectralFedAvg controls (truncation) from the part that no averaging rule can remove (client heterogeneity).

**Proposition 1 (Truncation and aggregation error).** *Fix an edge (i,j) and a round, and let* $p_{k}\;=\;n_{k}/n$ *for* $k\;\in \;{𝒮}_{t}$*. On client k, let* $\varphi^{\left(k\right)}(t)\;=\;\sum_{n=0}^{N}c_{n}^{\left(k\right)}T_{n}(t)$ *be the Chebyshev part of the edge function in* Eq. (6) *(with* $w_{ij}^{cheb}$ *absorbed into the coefficients), and let* ${\tilde{\varphi }}^{\left(k\right)}$ *be its truncation to the degree N*^*(k)*^ *selected by* Eq. (7)*. Let* $\bar{\varphi }$ *be the function defined by the averaged coefficients of* Eq. (8)*. Then*


$$\begin{array}{c} \|\varphi^{\left(k\right)}-{\tilde{\varphi }}^{\left(k\right)}{\|}_{L^{\infty }[-1,1]}\;\leq \;\sqrt{N-N^{\left(k\right)}}\;\sqrt{\tau }\;\|c^{\left(k\right)}{\|}_{2}, \end{array}$$ (9)

*and, for every client* $k\;\in \;{𝒮}_{t}$,


$$\begin{array}{c} \|\bar{\varphi }-\varphi^{\left(k\right)}{\|}_{L^{\infty }[-1,1]}\;\leq \;\sqrt{\tau }\sum_{j\in {𝒮}_{t}}p_{j}\sqrt{N-N^{\left(j\right)}}\;\|c^{\left(j\right)}{\|}_{2}\;+\;\Gamma_{k}, \end{array}$$ (10)

*where*


$$\begin{array}{c} \Gamma_{k}=\|\sum_{j}p_{j}\varphi^{\left(j\right)}-\varphi^{\left(k\right)}{\|}_{L^{\infty }[-1,1]}\;\leq \;\sum_{j}p_{j}\|c^{\left(j\right)}-c^{\left(k\right)}{\|}_{1}. \end{array}$$ (11)

*Proof.* Because $|T_{n}(t)|\;\leq \;1$ on [−1,1], $\left\|\varphi^{\left(k\right)}-{\tilde{\varphi }}^{\left(k\right)}{\|}_{\infty }\;\leq \;\sum_{n>N^{\left(k\right)}}|c_{n}^{\left(k\right)}\right|$, and by the Cauchy–Schwarz inequality this sum is at most $\sqrt{N-N^{\left(k\right)}}\;(\sum_{n>N^{\left(k\right)}}|c_{n}^{\left(k\right)}{|}^{2}{)}^{1/2}$; the tail energy is at most $\tau \|c^{\left(k\right)}{\|}_{2}^{2}$ by Eq. (7), which gives Eq. (9). Zero-padding to $N_{ij}^{*}$ changes the length of the coefficient vectors but not the functions they represent, so $\bar{\varphi }\;=\;\sum_{j}p_{j}{\tilde{\varphi }}^{\left(j\right)}$ and $\bar{\varphi }-\varphi^{\left(k\right)}\;=\;\sum_{j}p_{j}({\tilde{\varphi }}^{\left(j\right)}-\varphi^{\left(j\right)})+(\sum_{j}p_{j}\varphi^{\left(j\right)}-\varphi^{\left(k\right)})$. The triangle inequality and Eq. (9) bound the first term, and $|T_{n}|\;\leq \;1$ bounds $\Gamma_{k}$, which gives Eq. (10).

#### Interpretation of the bound.

Three points follow. First, zero-padding does not recover coefficients that a client discarded: the truncation error is set by each client’s own degree *N*^(*j*)^, not by the consensus degree $N^{*}$. Second, the truncation term is controlled by τ: with $\tau \;=\;10^{-3}$, *N* = 8, and $N^{\left(k\right)}\;\geq \;3$, the factor $\sqrt{N-N^{\left(k\right)}}\sqrt{\tau }$ is at most $\sqrt{5}\times 0.032\;\approx \;0.071$, so truncation changes an edge function by at most 7.1% of its coefficient norm. Third, $\Gamma_{k}$ measures how far a client’s own edge function lies from the population average; it is present for every averaging rule, including FedAvg, and is the term that personalised heads would reduce. When client edge functions are smooth, the Jackson and Bernstein theorems [36,37] explain why small degrees usually satisfy Eq. (7), because Chebyshev coefficients then decay algebraically or geometrically; this concerns local approximation and is not a convergence rate for federated training.

#### Scope of the bound.

The bound applies to one edge function. Each output coordinate of a KAN layer sums *d*_in_ edge functions, so its error is at most the sum of the corresponding per-edge bounds. The proposition does not bound the propagation of these errors through the nonlinear composition of layers, nor the convergence of the federated optimisation. A convergence analysis that tracks the round-varying degrees $N_{ij}^{*}$ together with client drift remains open; when every client keeps the full degree ($N_{ij}^{\left(k\right)}\;=\;N$), SpectralFedAvg reduces to FedAvg on the Chebyshev coefficients.

### Training, privacy, and evaluation

#### Federated training.

We use *T* = 100 rounds, *C* = 0.3, *E* = 2 local epochs, Adam ($\eta \;=\;10^{-3}$, weight decay 10^–4^, a fresh optimiser state per round), batch 64 (32 for MIMII, 256 for C-MAPSS) and gradient clipping at norm 1.0. **Baselines**: FedAvg [5], FedProx [3] (μ=0.01), SCAFFOLD [4] (option-II control variates, the correction added to the gradient before each optimiser step) and FedBN [28] use *the same* Mamba+Chebyshev-KAN model as SpectralFedAvg, so differences between them isolate the aggregation rule. **Local-only** trains each client alone for 60 epochs, selects its model on its own validation data and is evaluated on the union of all clients’ test sets; we report the mean over clients. The **pooled reference** trains the same Mamba+Chebyshev-KAN model on the union of all clients’ training data for 60 epochs. **SpectralFedAvg**: *N* = 8, $\tau \;=\;10^{-3}$. Every federated method is evaluated on the union of validation sets every five rounds, and the round with the best validation score is retained and evaluated once on the test sets. Experiments ran on one NVIDIA RTX A4000 Laptop GPU (8 GB) with PyTorch 2.12; the code is public at https://github.com/kmkholm/FedMamba-ChebKAN.

#### Example-level differential privacy.

In the private variant every client trains with DP-SGD [61]: at each local step it computes the gradient of every training window separately, clips each to norm *C* = 1, sums them, adds Gaussian noise $𝒩(0,\sigma^{2}C^{2}𝐈)$ and divides by the batch size, and Adam then uses this noisy gradient. Because the noise is added before anything leaves the client, the gateways, the coordinator and the other clients observe only post-processed outputs of a differentially private mechanism. The guarantee therefore does not rely on a trusted coordinator, and it covers the Chebyshev coefficients and the truncation degrees, which are computed from the noisy model. It is an example-level guarantee: it bounds what the released updates reveal about any single training window of a client. Three changes make the model compatible with per-example clipping: BatchNorm, which mixes the examples of a batch, is replaced by GroupNorm [62] with eight groups; every window is standardised by its own mean and standard deviation rather than by client statistics; and the model of the last round is reported, so that no model selection uses private validation data. Because DP-SGD degrades sharply when a model is trained from scratch on few examples [63,64], the encoder and the first Chebyshev-KAN layer are first trained on public data from a different test rig: the Paderborn healthy, outer-race and inner-race bearings under the two operating settings that the federated benchmark does not use, decimated to 12.8 kHz to match the CWRU time scale (14 438 training windows). Because the noise added per step is fixed while the summed gradient grows with the number of examples, each client in the privacy study uses every non-overlapping window of its training segments (639–924 windows per client instead of the 195–260 sampled for the main comparison; the recordings, splits, validation and test sets are unchanged), all of them in every step, with four local epochs per round, *T* = 100 rounds and a learning rate of $5\;\times \;10^{-3}$, with σ∈{4,8,15}. The privacy loss of each client is computed with the Rényi-DP accountant [65,66] (Google dp_accounting 0.6.0) from the number of noisy steps the client actually took, at $\delta \;=\;10^{-5}$, and we report the largest ε over the clients. These settings were chosen on seed 0 (the learning rate by validation accuracy), and the reported runs use seeds 1–3. The private variant is evaluated on CWRU, where heterogeneity across sensor positions is strongest.

#### Evaluation.

We define each metric formally for reproducibility. Let $y_{i}\;\in \;\{1,...,K\}$ denote the ground-truth class and ${\hat{y}}_{i}$ the predicted class for sample *i* in a test set of size *N*, and let ${TP}_{k},{FP}_{k},{FN}_{k}$ denote per-class confusion-matrix entries.

#### Classification (CWRU, Paderborn).

We report accuracy, macro-F1, and macro one-vs-rest AUROC, defined as


$$\begin{array}{cc} Acc & =\frac{1}{N}\sum_{i=1}^{N}1[{\hat{y}}_{i}\;=\;y_{i}], \end{array}$$ (12)

$$\begin{array}{cc} {F1}_{k} & =\frac{2\;{TP}_{k}}{2\;{TP}_{k}+{FP}_{k}+{FN}_{k}},\;Macro\text{-}F1=\frac{1}{K}\sum_{k=1}^{K}{F1}_{k}, \end{array}$$ (13)

$$\begin{array}{cc} Macro\text{-}AUROC & =\frac{1}{K}\sum_{k=1}^{K}{AUROC}_{k}(\text{class}\;k\;\text{vs. rest}), \end{array}$$ (14)

where 1[·] is the indicator function and ${AUROC}_{k}$ is the area under the receiver-operating-characteristic curve for the binary problem of class *k* versus all others. Metrics are computed on the union of the clients’ test sets; methods with client-local BatchNorm evaluate each client’s test windows with that client’s statistics. The centralised run trains the same Mamba+Chebyshev-KAN architecture on the pooled data of all clients; it is a pooled-data reference rather than a guaranteed upper bound, and its difference from FedMamba-ChebKAN is reported and tested explicitly.

#### Anomaly detection (MIMII).

For binary anomaly detection with continuous score s(x) and binary label y∈{0,1} (1 = anomalous), let TPR(θ)=Pr(s(x)≥θ∣y=1) and FPR(θ)=Pr(s(x)≥θ∣y=0). We report


$$\begin{array}{cc} AUROC & =\int_{0}^{1}TPR({FPR}^{-1}(u))\;du, \end{array}$$ (15)

$$\begin{array}{cc} AUPRC & =\int_{0}^{1}Prec({Rec}^{-1}(u))\;du, \end{array}$$ (16)

$$\begin{array}{cc} FAR@95\% & =FPR\;\left(\theta_{0.95}\right),\;\theta_{0.95}=\inf\{\theta :TPR(\theta )\;\geq \;0.95\}, \end{array}$$ (17)

where Prec(θ)=TP(θ)/(TP(θ)+FP(θ)) and Rec(θ)=TPR(θ). Following the MIMII protocol [55], the three metrics are computed per machine ID and averaged over the four IDs; the main results use the 0 dB clients, and the ±6 dB results are given in S1 Data.

#### RUL prediction (C-MAPSS).

For RUL regression with predictions ${\hat{y}}_{i}$ and targets $y_{i}$ over the *N* test engines of a subset (one prediction per engine) we report


$$\begin{array}{c} RMSE=\sqrt{\frac{1}{N}\sum_{i=1}^{N}({\hat{y}}_{i}-y_{i}{)}^{2}} \end{array}$$ (18)

together with the PHM08 asymmetric score of Eq. (1), which penalises late predictions (${\hat{y}}_{i}\;>\;y_{i}$) more strongly than early ones to reflect operational risk. Both are computed per FD subset and averaged over the four subsets.

#### Statistical tests.

Let $d_{r}\;=\;m_{r}^{prop}\;-\;m_{r}^{base}$ denote the per-seed performance difference, r∈{1,...,R} with *R* = 8. We use the paired two-sided Wilcoxon signed-rank statistic


$$\begin{array}{c} W=\min\;(\sum_{r:\;d_{r}>0}rank(|d_{r}|),\;\sum_{r:\;d_{r}<0}rank(|d_{r}|)), \end{array}$$ (19)

with *H*_0_: median$(d_{r})\;=\;0$, where differences are oriented so that a positive value favours the proposed method. The *p* value is exact: the null distribution is enumerated over all $2^{R}$ sign patterns of the observed ranks, which remains exact when differences are tied. With *R* = 8 seeds the smallest attainable two-sided *p* value is $2/2^{8}\;\approx \;0.0078$, so the test detects only differences whose sign is consistent across nearly all seeds; a non-significant result is not evidence of equivalence. The effect size is the rank-biserial correlation $r_{rb}\;=\;(W^{+}\;-\;W^{-})/(W^{+}\;+\;W^{-})$, which is positive when the proposed method is better. We control the false discovery rate with the Benjamini–Hochberg procedure [67], separately over all comparisons with federated baselines and over the comparisons with the pooled reference, and report raw *p* and adjusted *q* values.

## Results

All values are means ± standard deviations over *R* = 8 seeds, computed from the per-seed results in S1 Data; all federated methods and the pooled reference use the same Mamba+Chebyshev-KAN model (126 592 parameters for the vibration datasets).

### Performance across the four benchmarks

Fig 2 and Tables 3 and 4 summarise the main comparison.

**Table 3:** Classification on CWRU and Paderborn (*K* = 12 each).

Method	CWRU			Paderborn		
	Acc. (%)	Macro-F1	Macro-AUROC	Acc. (%)	Macro-F1	Macro-AUROC
Local-only	56.1±0.9	0.536±0.009	0.800±0.007	66.0±1.6	0.654±0.015	0.835±0.011
FedAvg	85.7±1.0	0.868±0.009	0.948±0.010	98.0±0.3	0.980±0.003	0.999±0.001
FedProx	87.2±1.6	0.882±0.015	0.955±0.013	98.4±0.3	0.984±0.003	0.999±0.000
SCAFFOLD	67.1±2.5	0.686±0.025	0.870±0.011	89.0±1.3	0.888±0.015	0.976±0.005
FedBN	98.0±0.8	0.982±0.007	0.999±0.001	97.9±0.8	0.979±0.009	0.999±0.000
**SpectralFedAvg**	85.5±0.9	0.867±0.008	0.946±0.012	98.2±0.4	0.982±0.004	0.999±0.001
**SpectralFedAvg-BN**	98.0±0.5	0.982±0.004	0.999±0.000	98.0±0.7	0.980±0.007	0.999±0.000
Pooled reference	98.7±0.2	0.988±0.002	1.000±0.000	99.3±0.2	0.993±0.002	1.000±0.000

Mean ± s.d. over *R* = 8 seeds on the union of the clients’ test sets; **bold**: best federated result.

**Table 4:** MIMII anomaly detection (*K* = 12, 0 dB clients, averaged over machine IDs) and C-MAPSS RUL prediction (*K* = 20, official test engines, mean over FD001–FD004).

Method	MIMII (fan, 0 dB)			C-MAPSS (FD001–FD004)	
	AUROC	AUPRC	FAR@95%↓	RMSE↓	Score↓
Local-only	0.790±0.001	0.792±0.001	0.542±0.003	31.24±0.56	54044±21178
FedAvg	0.804±0.008	0.798±0.005	0.514±0.021	20.89±0.63	5314±1472
FedProx	0.800±0.006	0.797±0.004	0.520±0.014	20.75±0.62	5432±1591
SCAFFOLD	0.735±0.015	0.737±0.010	0.593±0.055	60.19±19.88	$(1.3\pm 2.4)\times 10^{9}$
FedBN	0.803±0.007	0.798±0.005	0.512±0.015	20.78±1.01	5572±2712
**SpectralFedAvg**	0.805±0.006	0.799±0.004	0.505±0.016	20.41±0.60	4509±1351
**SpectralFedAvg-BN**	0.805±0.008	0.799±0.006	0.508±0.023	20.29±0.51	4005±1557
Pooled reference	0.806±0.002	0.791±0.002	0.519±0.014	19.98±0.24	3909±843

Mean ± s.d. over *R* = 8 seeds; ↓ lower is better.

**Fig 2:**
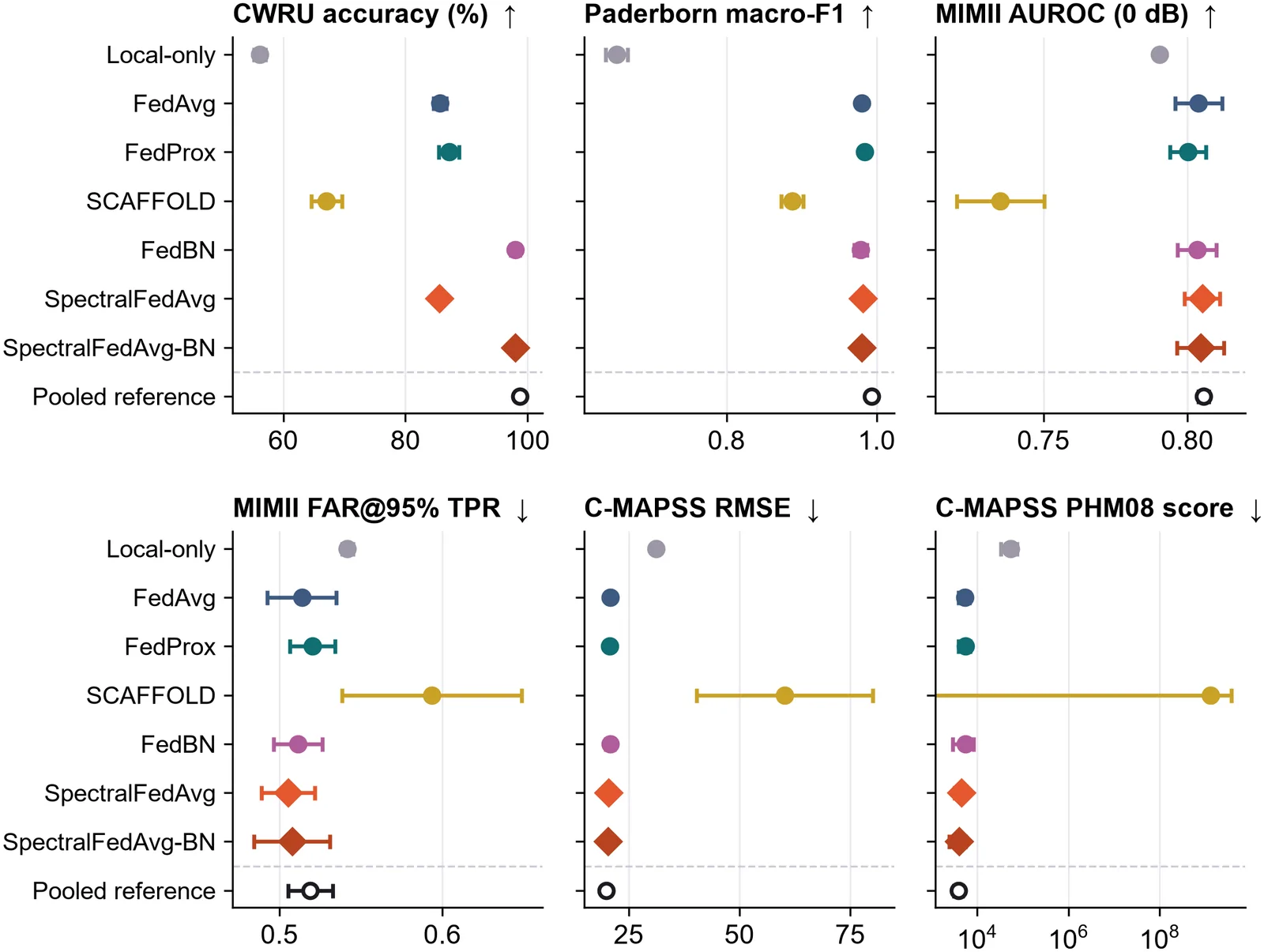
Performance of all methods on the four benchmarks. Each panel shows, for Local-only, FedAvg, FedProx, SCAFFOLD, FedBN, SpectralFedAvg, SpectralFedAvg-BN and the pooled reference, the mean over *R* = 8 seeds (marker) and ±1 standard deviation (horizontal bar). Diamonds mark the two SpectralFedAvg variants, the open marker the pooled reference, and arrows indicate whether higher (↑) or lower (↓) values are better. Top row: CWRU accuracy (%), Paderborn macro-F1 and MIMII AUROC on the 0 dB clients; bottom row: MIMII false-alarm rate at 95 % true-positive rate, C-MAPSS RMSE (cycles) and C-MAPSS PHM08 score on a logarithmic axis, because SCAFFOLD diverges in several seeds. All methods use the same Mamba+Chebyshev-KAN model, client partitions and training budget; the numerical values are listed in Tables 3 and 4.

#### CWRU.

The two methods that keep BatchNorm on the clients reach 98.0% accuracy (SpectralFedAvg-BN 98.0±0.5%, FedBN 98.0±0.8%), within 0.7 points of the pooled reference (98.7±0.2%). The methods that average BatchNorm statistics stay between 85.5 and 87.2%, and Local-only reaches 56.1%. Sensor position therefore dominates the heterogeneity on CWRU: drive-end, fan-end and base accelerometers see the same faults with different amplitude distributions, and averaging their normalisation statistics costs about 12 points; the remaining errors of SpectralFedAvg-BN lie among the fault classes, mainly inner-race windows predicted as outer-race or ball faults, and normal operation is never misclassified (Fig 3). **Paderborn.** All federated methods except SCAFFOLD lie between 97.9 and 98.4% (FedProx 98.4±0.3%, SpectralFedAvg 98.2±0.4%, SpectralFedAvg-BN 98.0±0.7%), against 99.3% for the pooled reference and 66.0% for Local-only, so sharing the three classes across clients makes this partition easy for every averaging rule. **MIMII.** AUROC lies between 0.800 and 0.805 for FedAvg, FedProx, FedBN and both SpectralFedAvg variants, close to the pooled reference (0.806); detection of fan anomalies at 0 dB is limited by the signal rather than by the aggregation rule. **C-MAPSS.** SpectralFedAvg-BN has the lowest RMSE (20.29±0.51) and PHM08 score among the federated methods, followed by SpectralFedAvg (20.41±0.60), FedProx (20.75), FedBN (20.78) and FedAvg (20.89), against 19.98±0.24 for the pooled reference; for every method the error grows from FD001 to the multi-condition, two-fault subset FD004 (Fig 4). Across all four datasets, SCAFFOLD is the least stable method: its control variates assume plain SGD steps, whereas every method here uses the same Adam optimiser, and on C-MAPSS it diverges in several seeds.

**Fig 3:**
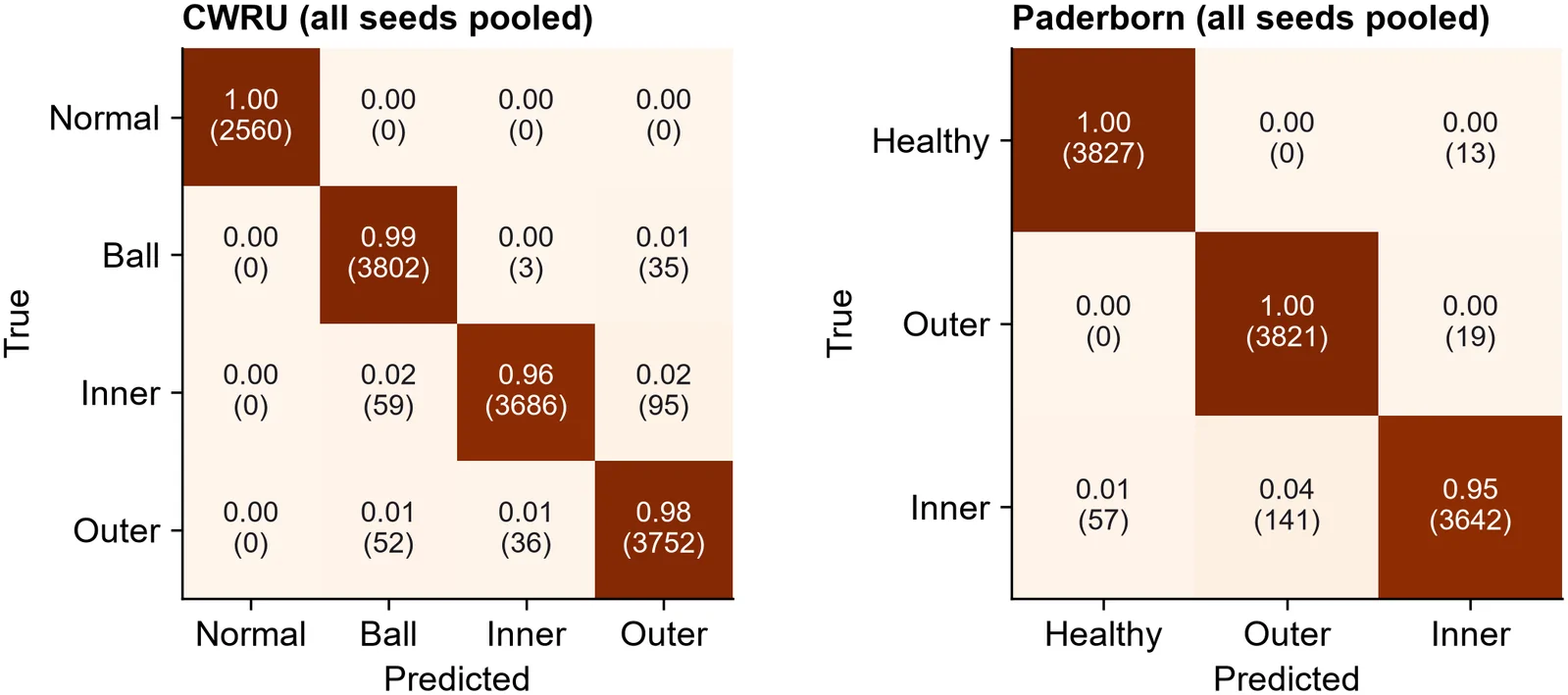
Test-set confusion matrices of SpectralFedAvg-BN on CWRU and Paderborn. Left: CWRU with the classes normal, ball fault, inner-race fault and outer-race fault; right: Paderborn with the classes healthy, outer-race damage and inner-race damage. Rows are true classes and columns predicted classes; each cell gives the proportion of the true class and, in brackets, the number of test windows, pooled over the test sets of all 12 clients and the eight seeds. Darker cells correspond to larger proportions.

**Fig 4:**
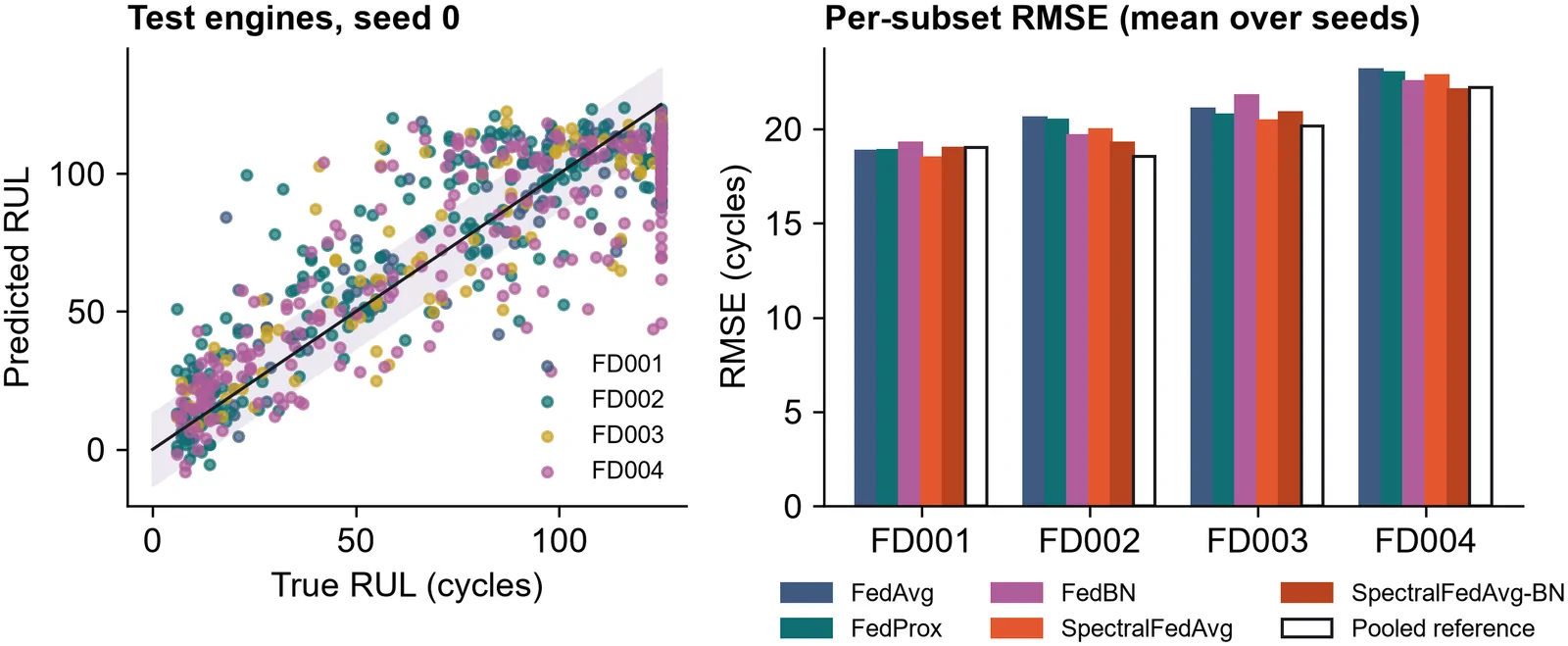
C-MAPSS remaining-useful-life prediction. Left: predicted versus true RUL of SpectralFedAvg (seed 0) for the 707 official test engines of FD001–FD004, one prediction per engine from its last window, coloured by subset; the black line is the identity, the shaded band marks ±13 cycles, and true RUL is capped at 125 cycles. Right: RMSE (cycles) per FD subset, mean over the eight seeds, for FedAvg, FedProx, FedBN, SpectralFedAvg, SpectralFedAvg-BN and the pooled reference (open bars); SCAFFOLD is omitted because it diverges in several seeds (Table 4).

### Statistical significance

Table 5 tests SpectralFedAvg-BN against the federated baselines and the pooled reference. On CWRU it is better than FedAvg, FedProx and SCAFFOLD in all eight seeds (*p* = 0.0078, *q* = 0.023, *r*_rb_ = + 1) and indistinguishable from FedBN (*p* = 1.0); it remains below the pooled reference (*p* = 0.016). On C-MAPSS its RMSE is lower than that of FedAvg after correction (*q* = 0.039), whereas the differences from FedProx (*q* = 0.083) and FedBN (*q* = 0.18) are not significant. On Paderborn no difference among the methods that share the architecture is significant except against SCAFFOLD, and on MIMII SpectralFedAvg-BN is significantly better than FedProx (*q* = 0.039) and SCAFFOLD but not than FedAvg or FedBN. With eight seeds, these non-significant differences should be read as small rather than as evidence of equivalence.

**Table 5:** Paired Wilcoxon signed-rank tests of SpectralFedAvg-BN (*R* = 8 seeds). Entries are exact two-sided *p* / Benjamini–Hochberg *q* / rank-biserial *r*_rb_ (positive favours SpectralFedAvg-BN). The adjustment covers 32 comparisons with federated baselines (eight metrics, see S1 Data) and, separately, eight comparisons with the pooled reference.

Dataset	Metric	vs. FedAvg	vs. FedProx	vs. SCAFFOLD	vs. FedBN	vs. pooled ref.
CWRU	Acc.	0.0078 / 0.023 / + 1.00	0.0078 / 0.023 / + 1.00	0.0078 / 0.023 / + 1.00	1.000 / 1.000 / + 0.00	0.016 / 0.031 / −0.94
Paderborn	Acc.	1.000 / 1.000 / + 0.00	0.266 / 0.386 / −0.47	0.0078 / 0.023 / + 1.00	0.984 / 1.000 / + 0.03	0.0078 / 0.031 / −1.00
MIMII	AUROC	0.383 / 0.510 / + 0.39	0.016 / 0.038 / + 0.94	0.0078 / 0.023 / + 1.00	0.383 / 0.510 / + 0.39	0.945 / 0.945 / −0.06
C-MAPSS	RMSE	0.016 / 0.038 / + 0.94	0.039 / 0.083 / + 0.83	0.0078 / 0.023 / + 1.00	0.109 / 0.184 / + 0.67	0.039 / 0.062 / −0.83

### Convergence and communication

Fig 5 shows the test metric of the global model during training. On CWRU the two methods with client-local BatchNorm pass 95% accuracy within 30–35 rounds and reach about 97.5% by round 100, whereas the methods that average normalisation statistics level off near 83–85% after about 30 rounds. On MIMII all methods except SCAFFOLD follow the same curve. Table 6 and Fig 6 report the measured upload. A FedAvg update of the 126 592-parameter model is 506 kB per participating client and round; SpectralFedAvg uploads 502 kB and SpectralFedAvg-BN 501 kB, because truncation removes only the negligible high-order tail of the Chebyshev block, which is itself a small part of the model. On CWRU, 67.6 % of the per-edge degrees stay at the maximum *N* = 8 and 17.9 % at 7 (C-MAPSS: 46.2 % at 8). Truncation is therefore not a communication saving at this model size; SCAFFOLD doubles the upload with its control variates.

**Table 6:** Communication cost on CWRU (*K* = 12, *T* = 100). Upload measured per participating client and round; nominal uplink = upload × ⌈CK⌉=4 × 100 rounds, ignoring dropout.

Method	kB/client/round	Nominal uplink (MB)	CWRU Acc. (%)
FedAvg	506.4	202.5	85.7±1.0
FedProx	506.4	202.5	87.2±1.6
SCAFFOLD	1012.7	405.1	67.1±2.5
FedBN	505.3	202.1	98.0±0.8
SpectralFedAvg	502.4	200.9	85.5±0.9
SpectralFedAvg-BN	501.2	200.5	98.0±0.5

**Fig 5:**
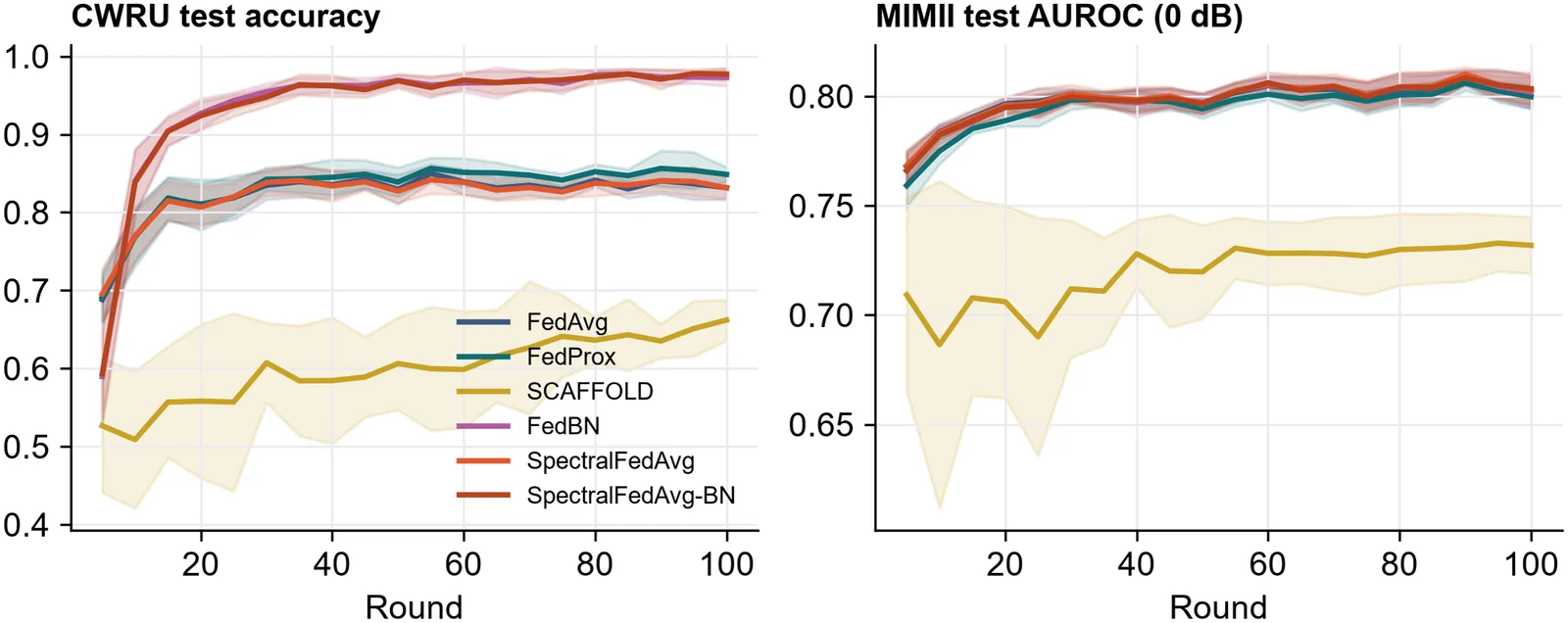
Convergence of the global model on CWRU and MIMII. Test accuracy on CWRU (left) and test AUROC on the 0 dB MIMII clients (right) of the global model, evaluated every five rounds over *T* = 100 rounds for FedAvg, FedProx, SCAFFOLD, FedBN, SpectralFedAvg and SpectralFedAvg-BN. Lines are means over the eight seeds and shaded bands ±1 standard deviation; on CWRU the two methods with client-local BatchNorm exceed 95 % accuracy within 30–35 rounds, whereas the methods that average normalisation statistics level off near 83–85 %.

**Fig 6:**
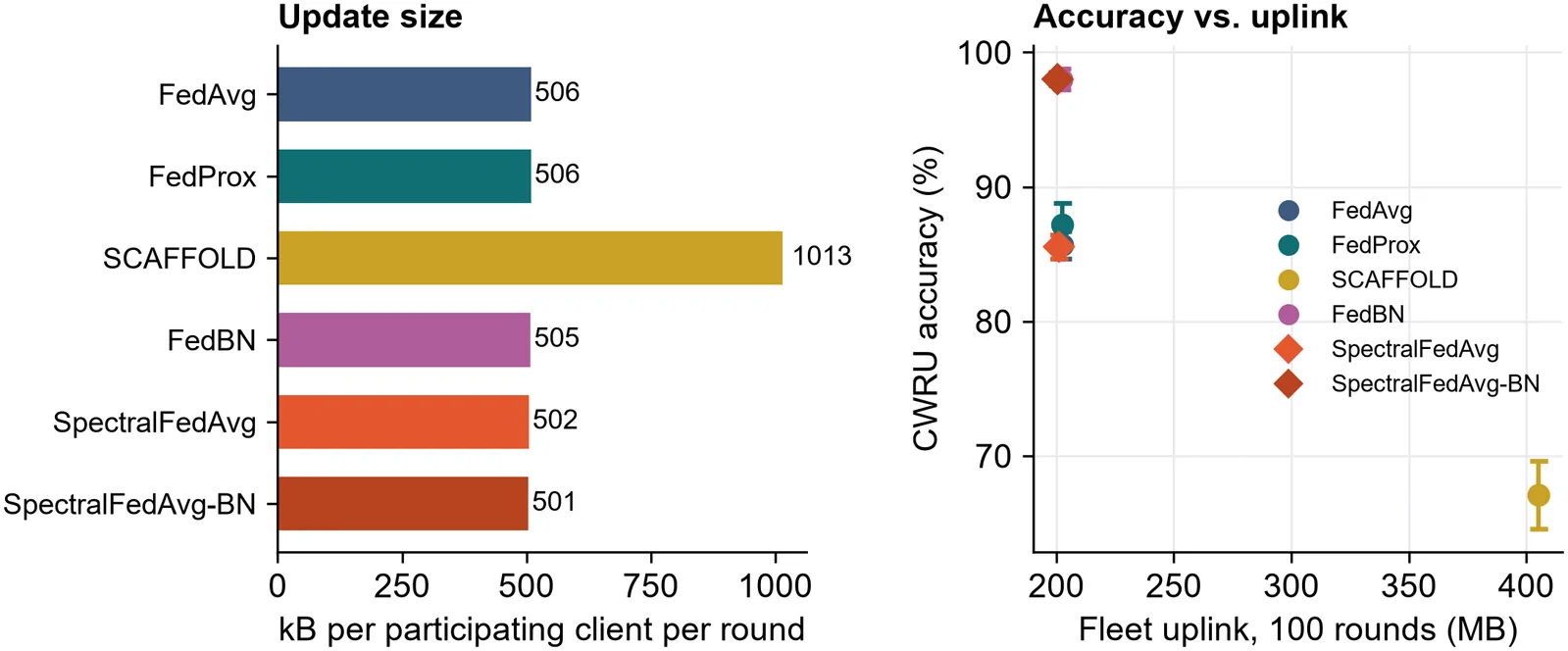
Communication cost on CWRU. Left: measured upload per participating client and round (kB) for FedAvg, FedProx, SCAFFOLD, FedBN, SpectralFedAvg and SpectralFedAvg-BN, computed from the transmitted tensors and including the bytes saved by truncation and the one-byte truncation degrees; SCAFFOLD also uploads its control variates. Right: CWRU accuracy (mean ±1 standard deviation over the eight seeds) against the nominal fleet uplink over 100 rounds, computed as the upload per client and round multiplied by four participating clients and 100 rounds.

### Aggregation and normalisation analysis

Table 7 and Fig 7 compare three configurations of the same model from the main runs: C (FedAvg), E (SpectralFedAvg) and E-BN (SpectralFedAvg-BN). Replacing FedAvg by SpectralFedAvg leaves accuracy on CWRU, Paderborn and MIMII within one standard deviation and lowers the C-MAPSS RMSE from 20.89 to 20.41; keeping BatchNorm on the clients adds about 12.5 points on CWRU and lowers the C-MAPSS RMSE further to 20.29, without changing Paderborn or MIMII.

**Table 7:** Aggregation and normalisation analysis from the main runs (mean ± s.d., *R* = 8 seeds).

Configuration	CWRU Acc. (%)	Paderborn Acc. (%)	MIMII AUROC	C-MAPSS RMSE↓
C: FedAvg	85.7±1.0	98.0±0.3	0.804±0.008	20.89±0.63
E: SpectralFedAvg	85.5±0.9	98.2±0.4	0.805±0.006	20.41±0.60
E-BN: SpectralFedAvg-BN	98.0±0.5	98.0±0.7	0.805±0.008	20.29±0.51

**Fig 7:**
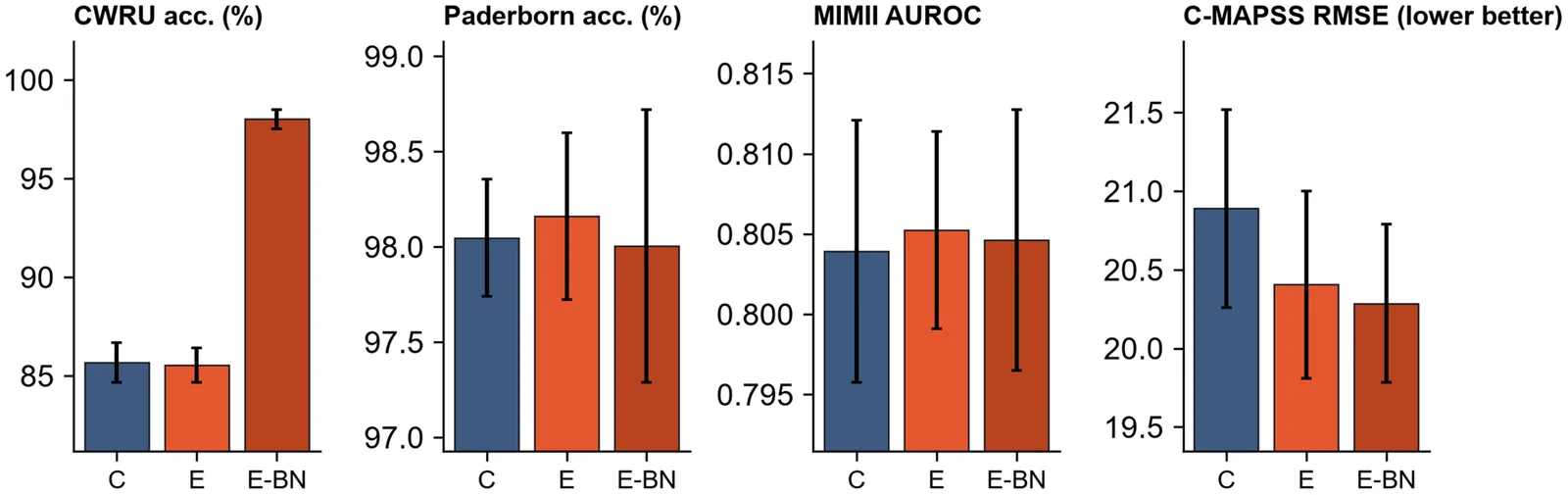
Aggregation and normalisation analysis. Bars compare three configurations of the same Mamba+Chebyshev-KAN model taken from the main runs: C (FedAvg), E (SpectralFedAvg) and E-BN (SpectralFedAvg-BN). Panels show CWRU accuracy (%), Paderborn accuracy (%), MIMII AUROC on the 0 dB clients and C-MAPSS RMSE (cycles, lower is better). Bar heights are means and error bars ±1 standard deviation over the eight seeds; the values are listed in Table 7.

### Explainability

Figs 8 and 9 examine a trained SpectralFedAvg-BN model on real CWRU test windows. Integrated gradients [68] attribute each prediction to the input samples. For the fault classes the attribution concentrates on the impulsive transients of the vibration signal, and in the outer-race window these impacts recur every 9.4 ms, the period of the outer-race defect frequency at this shaft speed (106 Hz at 1772 rpm) (Fig 8a). Removing one 250 Hz band at a time from the input shows that each decision rests on a few narrow bands (Fig 8b): 2.0–2.25 kHz for normal operation, 3.25–3.75 kHz and 4.0–4.5 kHz for the three fault classes, and in addition 0.5–0.75 kHz for the outer-race fault. These are the resonance regions in which bearing impacts are usually demodulated for this data set [53], and removing any other band lowers the probability of the true class by at most 0.06.

**Fig 8:**
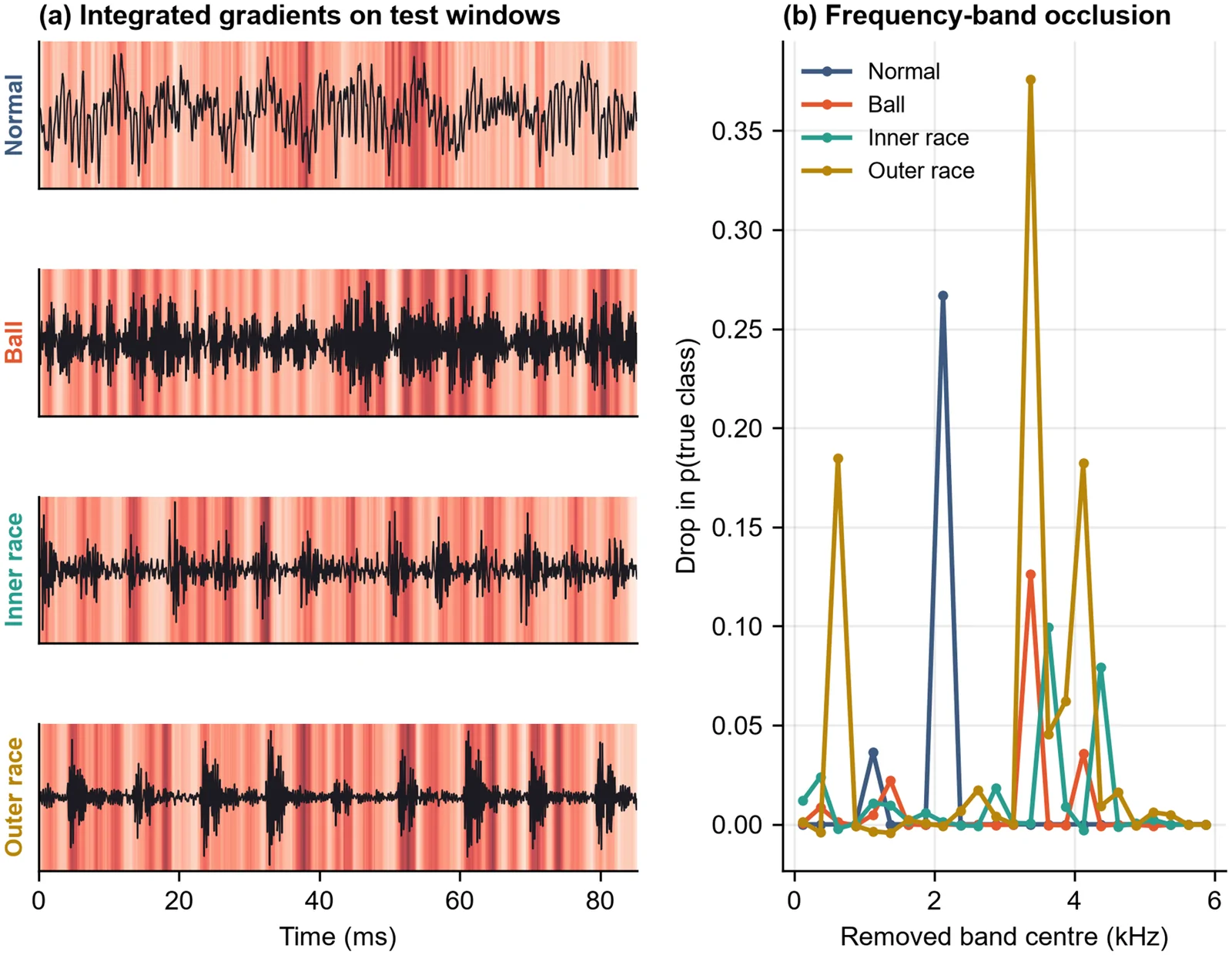
Attribution of SpectralFedAvg-BN predictions on real CWRU test windows. (a) One correctly classified test window per class (normal, ball, inner race, outer race) from the drive-end client at 1 hp load (1024 samples, 85 ms), with the integrated-gradients relevance of each sample shown as a red background (darker means more relevant; zero-signal baseline, 64 integration steps, moving average over 16 samples). The outer-race window shows impacts every 9.4 ms, the period of the outer-race defect frequency (106 Hz) at 1772 rpm. (b) Mean drop in the predicted probability of the true class when one 250 Hz band is removed from the input spectrum, computed over the correctly classified test windows of the drive-end and fan-end clients; the peaks mark the frequency bands on which the decision for each class depends.

**Fig 9:**
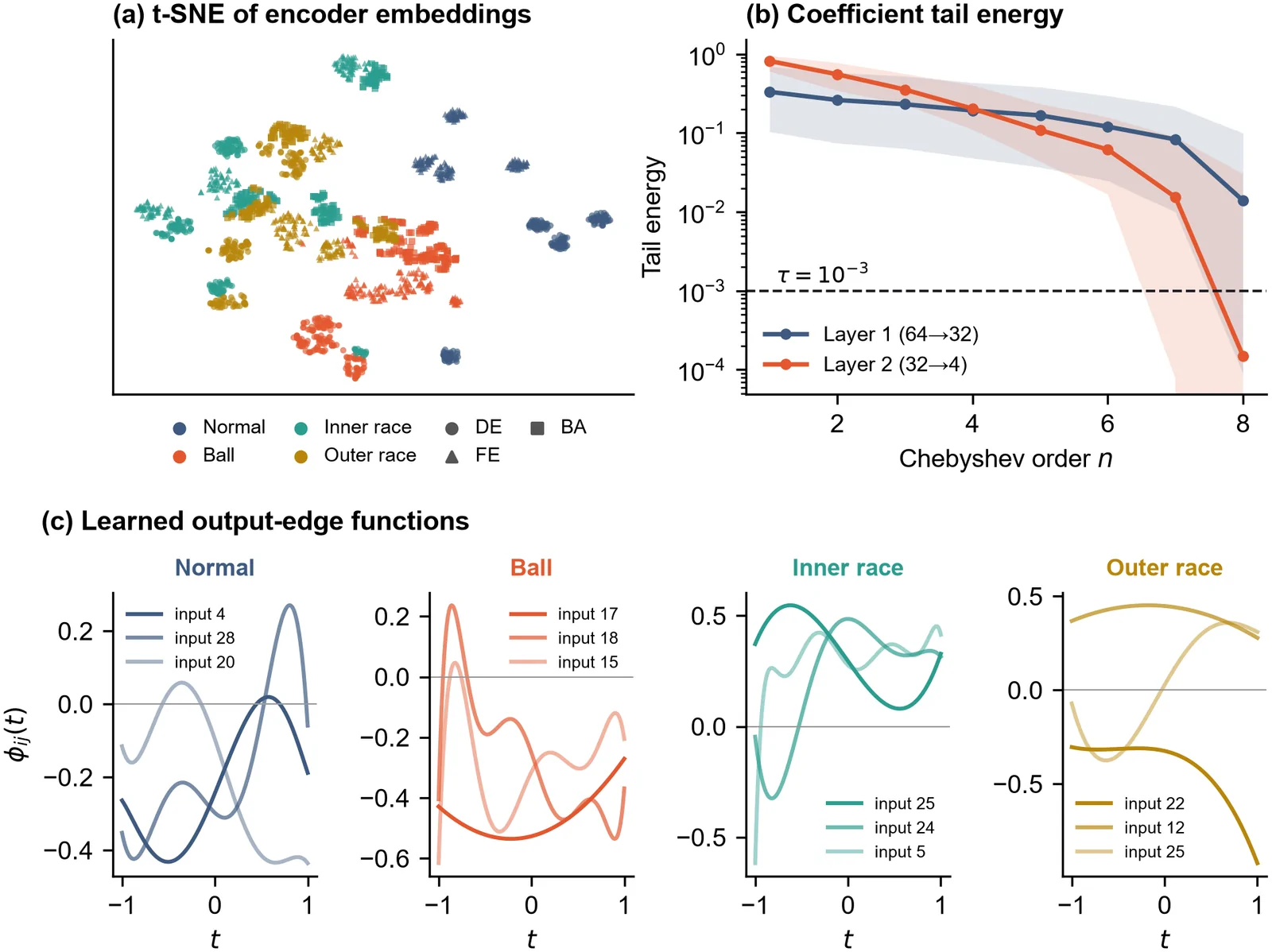
Representation and Chebyshev-KAN head of the trained SpectralFedAvg-BN model. (a) t-SNE projection of the 64-dimensional encoder embeddings of all CWRU test windows of the 12 clients, each computed with its client’s local BatchNorm, coloured by class, with marker shape indicating the sensor position (DE: drive end, FE: fan end, BA: base). (b) Tail energy $\sum_{m\geq n}c_{m}^{2}/\|c{\|}^{2}$ of the Chebyshev coefficients as a function of the order *n* for the first (64→32) and output (32→4) KAN layers; lines show the median and shaded areas the interquartile range over all edges of a layer, and the dashed line marks the truncation tolerance $\tau \;=\;10^{-3}$. (c) The three learned output-edge functions $\varphi_{ij}(t)$ with the largest magnitude for each class, plotted over the input range t∈[−1,1]; the legend gives the index *i* of the input feature.

In the embedding space of the encoder, visualised with t-SNE [69], the four classes form separate clusters that are further split by sensor position, the heterogeneity that client-local normalisation absorbs (Fig 9a). The Chebyshev coefficients decay with order: in the output layer the median tail energy falls below τ only at order 8, and in the first layer it stays above τ, so truncation acts mainly on the highest order of the output layer, in line with the small communication saving reported above (Fig 9b). The learned output-edge functions are smooth on [−1,1] and range from near-quadratic to oscillatory shapes (Fig 9c); they are reported as properties of the model, without physical labels for individual encoder features.

### Privacy–utility trade-off

Table 8 reports the private variant on CWRU. Without noise, the DP-compatible model (GroupNorm, per-window standardisation, public pretraining, all training windows) reaches 92.5±1.2% accuracy. With DP-SGD on every client, accuracy is 92.4±0.1% at ε≈19, 89.5±0.5% at ε≈8 and 85.6±1.1% at ε≈4 ($\delta \;=\;10^{-5}$). The cost of privacy at ε≈8 is therefore about 3 points.

**Table 8:** Privacy–utility trade-off on CWRU (*K* = 12). Example-level DP-SGD on every client with public pretraining and all non-overlapping training windows; mean ± s.d. over seeds 1–3, last-round model; ε is the largest over the clients ($\delta \;=\;10^{-5}$).

Setting	ε	Accuracy (%)	Macro-F1
No noise	∞	92.5±1.2	0.931±0.012
DP-SGD, σ=4	19.1	92.4±0.1	0.931±0.001
DP-SGD, σ=8	8.1	89.5±0.5	0.904±0.004
DP-SGD, σ=15	3.9	85.6±1.1	0.867±0.010

## Discussion

### Sources of the observed differences

Three findings stand out. First, client-local normalisation is the largest effect: when clients observe the same faults through different sensors (CWRU), keeping BatchNorm local raises accuracy by about 12 points, and SpectralFedAvg-BN and FedBN reach the same level. Second, on a fixed model the Chebyshev aggregation rule changes accuracy only slightly; its clearest benefit is on C-MAPSS, where SpectralFedAvg-BN attains the lowest RMSE among the federated methods and improves significantly on FedAvg. The basis-invariant parameterisation is what makes averaging the KAN head well posed, but at the scale of this model truncation rarely activates and contributes little to accuracy or communication. Third, when clients share the full label space (Paderborn) or when the task is limited by signal quality (MIMII at 0 dB), all reasonable aggregation rules perform alike. These results argue for client-local normalisation together with a basis-invariant head as the practical configuration, rather than for the aggregation rule alone.

#### Contextual comparison with externally published results.

Tables 3 and 4 report only methods run under our protocol, because protocol differences make numerical juxtaposition with published numbers misleading. FedSNGP [1] reports 99.81% on Paderborn under an open-set label-shift scenario with a different partition. VibrMamba [11] reports 99.77% on CWRU under centralised training with no partition, which shows that the Mamba backbone is capable but says nothing about federated training. On MIMII, the original autoencoder baseline [55] reports per-machine AUROC values for fans in a similar range at 0 dB. On C-MAPSS, the published LSTM [59] (RMSE 16.14) and attention-GRU [60] (RMSE 12.83) results are centralised and single-subset or single-seed, whereas our values average the four subsets, including the multi-condition FD002 and FD004, over 20 clients. The relevant reference for our method is the pooled-data run of the same model, which we report and test.

### Privacy: scope and implications

The private variant gives every client an example-level (ε,δ) guarantee that holds against every party observing its updates, including the coordinator, because the noise is added on the client before anything is uploaded. This is a stronger threat model than perturbing the aggregate at the coordinator, which presumes a trusted coordinator, and it extends to the transmitted Chebyshev coefficients and truncation degrees as post-processing, so the spectral aggregation of SpectralFedAvg needs no separate protection. The measured cost is small: at ε≈19 accuracy is within 0.1 points of the same model without noise, at ε≈8 it is 3.0 points lower, and at ε≈4 the private model still reaches 85.6%, with four clients sampled per round and a few hundred windows per client. Two design choices make this possible: public pretraining of the encoder on a different bearing test rig, which leaves the guarantee unchanged, and use of every training window of a client, which raises the summed gradient relative to the fixed noise of each step. The guarantee protects individual windows of 1024 samples; protection of whole recordings follows by group privacy, and client-level guarantees, which would also hide a client’s participation, are a natural extension with secure aggregation [16] and larger fleets.

### Limitations and future work

The federated setting is simulated: the gateway tier forwards updates unchanged, intermittent participation is modelled by random dropout, and inference latency, memory and energy on edge hardware were not measured. The model is small (about $1.3\;\times \;10^{5}$ parameters, 0.5 MB at float32), which suits mid-tier edge devices, but on-device training under a duty cycle requires hardware-in-the-loop measurement. All methods share one optimiser (Adam); SCAFFOLD, which was designed for SGD local steps, was unstable under this choice, so its numbers should not be read as representative of SCAFFOLD with SGD. On C-MAPSS our federated RMSE values lie above the best centralised results in the literature, partly because they average the multi-condition subsets. CWRU and C-MAPSS come from laboratory rigs or simulation and may not capture sensor drift, maintenance interventions or evolving operating policies; MIMII uses factory recordings with controlled SNR mixing. Client distributions are assumed stationary during training. With eight seeds the Wilcoxon test detects only consistent differences. Per-edge Chebyshev coefficients can be inspected directly, but we did not validate any mapping between encoder features and physical quantities and therefore make no claim of mechanical interpretability. Directions that follow directly from this work are drift-aware continual FL, client-level privacy with secure aggregation on larger fleets, personalised Chebyshev-KAN heads that keep coefficient vectors local while aggregating the encoder, and on-device benchmarking.

## Conclusion

We presented FedMamba-ChebKAN, a federated framework for industrial fault detection that pairs a Mamba selective state-space encoder with a Chebyshev-KAN head and aggregates the head with SpectralFedAvg. Because the Chebyshev basis is identical on every client, coefficient averaging is well posed without re-interpolation, and Proposition 1 bounds the error of the aggregated edge function by a truncation term plus a client-heterogeneity term. Under a protocol with splits made before windowing, all federated baselines on the same architecture and eight seeds per configuration, SpectralFedAvg-BN reaches 98.0% accuracy on CWRU, 12.3 points above FedAvg and level with FedBN, and the lowest federated RMSE on C-MAPSS (20.29), while all methods that share the architecture perform alike on Paderborn and MIMII. Client-local normalisation is the decisive ingredient under sensor heterogeneity, and truncation saves little communication at this model size. With DP-SGD on every client and public pretraining, the model retains 89.5% CWRU accuracy at example-level ε≈8, a guarantee that does not depend on a trusted coordinator. Code, client partitions and per-seed results are public to support direct comparison under the same protocol.

## Supporting information

S1 Data Per-seed results. One row per run (dataset, method, seed and configuration, including the privacy study) with every reported metric, the selected round, the measured upload and the run time (CSV); every mean, standard deviation and test in the paper can be recomputed from this file.
(CSV)

S2 File Client partitions and configuration. Client-to-recording, client-to-clip and client-to-engine assignments for every dataset, and all architecture, optimisation and federated settings.
(JSON)
