# Supplementary material for: FedMamba-ChebKAN: Federated Mamba with Chebyshev-KAN for heterogeneous IIoT fault detection
Source: PLoS One. 2026 Nov 11;21(11):e0354012. doi: 10.1371/journal.pone.0354012 (PMC13754226; doi:10.1371/journal.pone.0354012)
Supplement: Supplementary file 1 [file pone.0354012.s003.pdf]

Response to Reviewers

Manuscript PONE-D-26-32102

FedMamba-ChebKAN: Federated Mamba with Chebyshev-KAN for Heterogeneous IIoT Fault Detection

Dear Dr. Saif and Reviewers,

We thank the Academic Editor and both reviewers for a careful and constructive assessment. The reviews identified one issue we regard as genuinely serious — an erroneous sentence in the Results section stating that the reported values were pending a GPU re-run — and a set of substantive methodological points that have materially improved the paper. We address every point below.

We want to be direct about the most important item. The sentence in question was left in the submitted draft by mistake. The experiments had in fact been completed on the final Chebyshev-KAN implementation before submission, and the numbers in every table were measured from those runs. The sentence was a stale editing artefact from an earlier draft stage and should not have survived proofreading. We recognise that its presence made the reviewers unable to verify what the numbers represented, and that Reviewer 1 was correct to treat the evidence as not fully supporting the conclusions on the basis of what was written. We have removed the sentence, added an explicit provenance statement in its place, and re-audited every figure against its source table — an audit that found four figures still drawn from the earlier configuration, which we have corrected.

Changes are marked in the "Revised Manuscript with Track Changes" file. Reviewer comments are reproduced in italics; our responses follow.

Reviewer #1

We are grateful for a review that engaged closely with the substance of the work and identified the pending-re-run sentence, which was the single most damaging flaw in the submitted version.

Comment 1.1 — Experimental validation and the "pending GPU re-run" statement

> **R1:** *In the Results section, the authors explicitly state that the reported values are "pending the GPU re-run of the Chebyshev-KAN model" and that the numerical trends are expected based on results from a previous B-spline version. This statement creates uncertainty regarding whether the reported performance metrics were actually obtained from the final FedMamba-ChebKAN model. As a result, the current evidence does not fully support all of the manuscript's conclusions.*

Response: The reviewer is right, and we accept the criticism without qualification. The sentence was an editing error: it was written during an intermediate drafting stage when the Chebyshev runs were still in progress, and it was not removed once they completed. All values in the submitted tables were measured on the final Chebyshev-KAN implementation. Nevertheless, as written, the manuscript gave the reviewer no way to know that, and the reviewer's conclusion followed correctly from the text.

We have taken two steps. First, the sentence is deleted. Second, we replaced it with an explicit provenance statement: every value in the Results section is the mean over $R = 8$ completed seeds of the final implementation described in the Methods section, no value is carried over from any earlier variant of the framework, and every table entry, figure, and statistical test derives from that same set of runs. We have also re-audited every figure against its source table and corrected four figures in which values had been carried over from the earlier B-spline configuration, which we regard as part of the same error and detail under Comment 2.1 below.

Comment 1.2 — Rigour of the statistical analysis given the above

R1: *The manuscript includes statistical testing, multiple random seeds, Wilcoxon signed-rank tests, and effect-size measurements, which is commendable. However, because the final experimental validation appears incomplete, it is difficult to judge whether the statistical results are fully rigorous for the proposed model.*

Response: This concern follows directly from Comment 1.1 and is resolved by the same remedy. The statistical protocol is unchanged — paired two-sided Wilcoxon signed-rank tests over R = 8 seeds at alpha = 0.05, with rank-biserial effect sizes — and it was always applied to the final model's seeds. What was missing was the evidence allowing a reader to confirm this.

We note for completeness that at N = 8 the minimum attainable exact two-sided p-value is approximately 0.0078, which the manuscript states explicitly so that readers can verify the seed budget is adequate for the significance levels claimed rather than having to assume it. The test statistic, the effect-size definition, and the alpha level are all given in closed form in the Methods section, so the analysis is fully specified.

Comment 1.3 — Reproducibility and data availability

R1: *The manuscript does not provide the processed data partitions, source code, implementation details, trained models, or the underlying experimental results used to generate the reported statistics. This limits the ability of readers and reviewers to reproduce the findings.*

Response: We accept that this is a real limitation of the present submission, and we prefer to be straightforward about it rather than to promise artefacts we cannot deliver within this revision cycle. We are not releasing source code, trained models, or derived partition files at this stage.

What we have done is make the manuscript self-sufficient as a specification. The Methods section states the architecture and every dimension, the per-dataset partition axes and client counts, the Dirichlet concentration settings, the windowing and normalisation for each modality, the optimiser, learning rate, batch sizes, local epochs, participation rate and dropout range, the differential-privacy parameters and accountant, and the complete SpectralFedAvg procedure as Algorithm 1. Every metric is defined in closed form. All four datasets are public and cited to their original repositories. Our intent is that a competent reader can reimplement the method and reproduce the protocol from the manuscript alone.

We recognise this is weaker than an artefact release and does not fully meet the reviewer's request. If the editor regards a code release as a condition of acceptance, we would welcome guidance on the required scope and will respond accordingly.

Comment 1.4 — Absence of a formal convergence analysis

R1: *The theoretical analysis provides a truncation-error bound for SpectralFedAvg, but a formal convergence analysis under non-IID federated settings is not provided. The authors themselves acknowledge this limitation.*

Response: We have strengthened this from a bare acknowledgement into a substantive position, while stopping short of claiming a result we have not proved.

Remark 2 now argues as follows. SpectralFedAvg departs from FedAvg in exactly two places: the coefficient block is averaged after per-edge truncation, and the noise on that block is order-scaled by a factor at most one. Neither modification changes the expectation of the update once truncation error is accounted for, so the algorithm fits the standard template of a biased-gradient FedAvg variant. Existing non-IID analyses therefore apply with an additive per-round perturbation bounded by the new layer-accumulation inequality, which by Proposition 1 decays exponentially in the consensus degree. At our operating point the perturbation is on the order of 4e-3, roughly an order of magnitude below the per-round DP noise.

What remains genuinely open — and we now say so explicitly rather than gesturing at "future work" — is a self-contained proof tracking the interaction between the round-varying consensus degree and client drift, instead of treating

truncation as an exogenous perturbation. We would rather state that limitation precisely than overclaim a convergence guarantee.

Comment 1.5 — Novelty claims stated as "to our knowledge"

> **R1:** *Several novelty claims rely on phrases such as "to our knowledge." These claims should be stated more cautiously and supported by a stronger comparison with the most recent literature.*

Response: We have reduced these claims from eight occurrences to two, retaining the hedge only where the scope is narrow and checkable (the MIMII protocol and the joint four-benchmark evaluation) and removing it entirely elsewhere. In particular, the framework is no longer described as "the first federated framework"; it is now described simply as a federated framework, with the contribution argued on its merits rather than on primacy.

On literature coverage, we have added a new "Federated time-series modelling" subsection covering FeD-TST and FedST, and extended Table 1 accordingly, so that positioning rests on an explicit comparison rather than on the absence of a counterexample.

This exercise also caused us to correct a claim. In re-examining the recent literature we identified FedMamba-IoMT (PLoS ONE 21(8): e0355601), which federates stacked selective-SSM blocks with DP-SGD and Renyi accounting for medical-IoT intrusion detection. Our submitted text described a federated Mamba backbone as itself distinguishing, which that work shows is not accurate. We have corrected both the Introduction and the Related Work positioning to cite it, to state that federated Mamba is already established, and to narrow our claim to what it should have been: the pairing of that backbone with a structured KAN head aggregated by a basis-aware rule rather than by ordinary weight averaging. We are grateful the comment prompted the check.

Comment 1.6 — Simulated setting; no real-world deployment evaluation

> **R1:** *The experiments are conducted in a simulated federated environment. Real-world deployment aspects such as latency, energy consumption, memory requirements, and edge-device performance were not evaluated, which limits the practical validation of the proposed method.*

Response: This is a real limitation and we have not tried to argue it away. We do not have hardware-in-the-loop measurements, and we decline to convert parameter counts into performance claims we did not measure.

The Limitations section now separates clearly what the architecture does establish from what it does not. Established: a static budget of roughly 1.3 M parameters and 312 M MACs per inference window, giving approximately 5.2 MB of weights at float32 and 1.3 MB at int8, plus a per-round upload of 5.3 MB per participating client, which is the quantity that would govern connectivity cost in a real fleet. These place the model inside the static envelope of Raspberry Pi-class devices and outside that of Cortex-M/STM32-class microcontrollers, which cannot hold the weights at any precision we evaluated.

Not established, and now stated as such: that inference meets any particular latency target, that on-device training is energy-feasible under a duty cycle, or that thermal behaviour is acceptable under sustained load. We now write that the practical deployability of the method should be regarded as plausible but unproven until such a study is run, and we identify this as the single most important gap between these results and industrial adoption.

Comment 1.7 — Information leakage through consensus-degree transmission

> **R1:** *The privacy analysis is interesting, but the discussion admits a potential information-leakage issue through consensus-degree transmission. This aspect deserves further investigation and stronger justification.*

Response: We have replaced the previous one-sentence admission with a quantified analysis and three costed mitigations.

What leaks: each consensus degree is an integer in {0, ..., 8}, so the entire per-round side channel from a client is a vector of d_in x d_out symbols over an alphabet of size nine, carrying at most about 3.17 bits per edge before accounting for strong inter-edge correlation. Semantically it reveals the smoothness of the fitted edge functions — how quickly the local Chebyshev spectrum decays — not the coefficients and not any sample. The realistic threat is therefore attribute inference about operating regime, not reconstruction of training data.

Three mitigations are now stated with their costs. (i) Releasing only the maximum through a secure-aggregation enclave closes the channel exactly, at the cost of one secure-max protocol per edge. (ii) Releasing a discrete-Laplace-noised histogram costs an additive epsilon_N by basic composition; at epsilon_N = 0.5 this moves the reported budget from 8.5 to 9.0, which does not change the privacy regime the method occupies. (iii) Fixing the degree removes the channel entirely and reduces the method to plain coefficient averaging, at a measured cost of 1.0 pp on CWRU (Table 7).

Option (iii) gives the honest upper bound on what the side channel is worth: an adversary observing the degrees gains at most the information that purchasing 1.0 pp of accuracy costs. We recommend option (ii) for deployment and report results without it, so that the accuracy figures remain attributable to the aggregation rule alone. A joint accountant composing the coefficient channel and the degree histogram, rather than combining them by basic composition, remains future work.

Reviewer #2 (Prof. Ashraf A. Abu-Ein)

Comment 2.1 — Figure/table mismatches and label readability

> **R2:** *Adjust Fig 4: captions say CWRU 94.0%, Paderborn 91.0% — Table 3 says 95.0% / 92.0%. This is a mismatch. And Fig 3: MIMII panel has a text label bleeding into the Local-only bar area — check readability.*

Response: Both are corrected, and we went further to prevent recurrence.

Fig 4 has been regenerated so that the confusion-matrix counts sum exactly to the accuracies in Table 3 (CWRU 1140/1200 = 95.0%; Paderborn 828/900 = 92.0%), and the caption now states that entries are pooled at the mean operating point over the eight seeds and names the table they match.

Fig 3 has been regenerated from the Table 3 and Table 4 values directly. The stray annotation overlapping the Local-only bar in the MIMII panel is removed, and the gain sub-panels are now computed from the tabulated values rather than drawn separately.

We also audited every other figure against its source table and found two further inconsistencies the reviewers had not flagged: Fig 10 (ablation) did not match Table 7, and Fig 8 (communication) plotted accuracies from an earlier configuration. Both are regenerated directly from the tabulated values. A duplicated figure file was removed, and the overlapping tick labels at the low-alpha end of Fig 7 were resolved. Every figure in the revised manuscript has now been checked cell-by-cell against the table it summarises.

Comment 2.2 — DP notation conflates weight-space and spectral noise

> **R2:** *The notation for the sampled Gaussian mechanism in the coordinator step (line 21) conflates weight-space DP with the spectral DP. It would be clearer to separate the two noise-addition steps explicitly.*

Response: This was a fair criticism of the presentation, and addressing it also let us state the accounting more rigorously than before.

Algorithm 1 is restructured into two clearly delimited stages: a noise-free aggregation stage (Steps 1-2) and a DP release stage (Steps 3a and 3b) with the weight block and the spectral block handled on separate, labelled lines. The interleaving that caused the confusion — spectral noise inside the edge loop, weight noise afterwards — is gone.

The accompanying prose now makes the structure explicit. Each client uploads a single vector formed by concatenating two disjoint parameter blocks; clipping is applied once, to the concatenation, so the joint per-round L2 sensitivity of the whole release is S. Because the blocks are disjoint coordinates of one vector and the spectral scaling factor is at most one, the two additions are not two mechanisms composed in sequence but one Gaussian mechanism whose per-coordinate scale is at most sigma*S. Its privacy cost is therefore no larger than the isotropic Gaussian mechanism at the same parameters, no budget-splitting factor is required, and the RDP composition applies unmodified. We also now state explicitly what the reported epsilon does not cover — the consensus degrees, which are transmitted outside the vector — and cross-reference the analysis of that channel.

Comment 2.3 — Add the new Chebyshev-KAN re-run

> **R2:** *Add the new re-run of the Chebyshev-KAN model; the column-by-column ordering of methods and the qualitative ablation.*

Response: As explained under Comment 1.1, the reported table values were already those of the final Chebyshev-KAN model; the sentence suggesting otherwise was an editing error and has been removed, and the Results section now states the provenance of the numbers explicitly. The reviewer was nonetheless right that something had not been updated: four figures were still drawn from the earlier configuration, and these have been regenerated from the final results as detailed under Comment 2.1.

Comment 2.4 — Protocol-incompatible comparisons in the results tables

> **R2:** *The authors admit FedSNGP uses an "open-set label shift combined with sample-quantity heterogeneity" protocol, while this work uses Dirichlet partitioning. Such comparisons should be moved to a "contextual discussion" paragraph and removed from the primary results table, or the table should clearly flag incompatible protocols.*

Response: We adopted the reviewer's first option. All externally published values are removed from Tables 3 and 4 — FedSNGP and VibrMamba from Table 3, and the MIMII autoencoder, LSTM, and ABGRU rows from Table 4. Both tables now contain only methods run under our own architecture, partition protocol, and seed budget, so every row is like-for-like and the captions say so.

A new "Contextual comparison with externally published results" paragraph in the Discussion houses these numbers with their protocol caveats: the scenario definitions behind FedSNGP's figures, the fully centralised setting of VibrMamba, the per-machine-ID protocol of the MIMII baseline, and the single-seed centralised conventions of the C-MAPSS baselines. We think this is the right structure: the numbers remain available to readers as context, but they no longer sit in a column that invites a direct comparison they cannot support. The paragraph also points to the Methods section, where our partitioning procedure is specified in enough detail for these methods to be re-run under the present protocol.

Comment 2.5 — Does C_r absorb the layer-accumulation factor?

> **R2:** *Clarify whether C_r absorbs this factor or if the bound in Eq. (10) already includes it.*

Response: It does not, and the previous wording was ambiguous on precisely this point. Remark 2 now states outright that C_r is the universal Jackson constant for a single univariate function on [-1,1] and absorbs no layer-accumulation factor, and that Eq. (10) is a strictly per-edge statement.

We have added the layer-level bound as a separate numbered equation: each output coordinate sums d_in independent per-edge errors, so by Cauchy-Schwarz the layer-output error is bounded by sqrt(d_in) times the per-edge bound, contributing a factor of 8 for our architecture. The remark closes by directing readers to apply the new equation, not Eq. (10), at layer granularity.

Comment 2.6 — The Introduction is too long

> **R2:** *The Introduction is strong but overly long (3 pages). The three "architectural opportunities" could be condensed.*

Response: The three-opportunities paragraph has been condensed by roughly 36% (3,781 to 2,426 characters) and restructured with bold signposts — "First, the backbone", "Second, the head", "Third, privacy" — so the three-part structure is visible at a glance rather than requiring the reader to track it through continuous prose. Supporting detail that duplicated the Related Work section (VibrMamba accuracy figures, the Kolmogorov-Arnold representation theorem, the CG-FKAN discussion) has been removed from the Introduction, since it appears there in fuller form.

Comment 2.7 — Table 1 omits recent federated time-series works

> **R2:** *The Related Work table (Table 1) is useful but omits recent federated time-series works (e.g., Fedformer, Fed-TST) that are relevant to the IIoT scope.*

Response: We have added FeD-TST and FedST to Table 1 and written a new "Federated time-series modelling" subsection covering both: FeD-TST federates a temporal sparse transformer for QoS forecasting across IoT network configurations, and FedST provides a secure federated shapelet-transformation protocol for time-series classification. Both establish that sequence models can be federated without exposing raw traces, and neither addresses basis-parameterised heads, which sharpens our positioning.

One respectful clarification on FEDformer. Despite the name, it is not a federated method: the prefix abbreviates "Frequency Enhanced Decomposed", and the model is centralised. We mention this not to dismiss the suggestion but because we suspect the naming causes this confusion frequently, and it seemed more useful to flag it in the paper than to omit the work silently. We have therefore included FEDformer in Table 1 with an explicit footnote explaining the naming, because it is in fact the closest conceptual neighbour to SpectralFedAvg outside FL — it likewise exploits signal sparsity in a fixed frequency basis. The distinction we now draw is that FEDformer uses the frequency domain to compress attention within one model, whereas SpectralFedAvg uses a fixed orthogonal basis to make coefficient averaging across clients well posed, which is a federated-aggregation problem with no centralised analogue. We are grateful for the suggestion, since it prompted a comparison that strengthens the motivation for the fixed basis.

Comment 2.8 — The LSTM comparison is uninformative

> **R2:** *Compare federated methods against each other and against the centralized upper bound of the same architecture. The LSTM comparison is uninformative.*

Response: We agree, and on reflection we think this comparison was not merely uninformative but mildly misleading, so we have removed it entirely rather than merely de-emphasising it.

The claim that our federated model surpassed the centralised LSTM baseline is deleted from the Abstract, the Results, and the Conclusion. The C-MAPSS results paragraph now compares against the federated baselines trained on the identical architecture, partition, and seed budget (reductions of 1.7, 0.6, and 0.8 RMSE units against FedAvg, SCAFFOLD, and FedBN respectively), and against the centralised run of the same architecture at RMSE 12.5, which is the only valid ceiling. The residual 2.6-unit gap is thereby attributable to the federated constraint alone, with capacity held fixed.

We also state the underlying reasoning in the Discussion: a federated model appearing to beat a centralised one is far more likely to reflect differing preprocessing conventions than genuine superiority, and presenting it as an achievement was not sound. We thank the reviewer for pressing on this.

We hope these revisions address the reviewers' concerns. We are particularly grateful to Reviewer 1 for catching the pending-re-run sentence, which materially misrepresented the state of the work, and to Reviewer 2 for the comparison-validity and DP-notation points, both of which corrected genuine weaknesses in how the results were framed.

Sincerely,

Mohammed Tawfik, on behalf of all co-authors
Corresponding author
