# Supplementary material for: FedMamba-ChebKAN: Federated Mamba with Chebyshev-KAN for heterogeneous IIoT fault detection
Source: PLoS One. 2026 Nov 11;21(11):e0354012. doi: 10.1371/journal.pone.0354012 (PMC13754226; doi:10.1371/journal.pone.0354012)
Supplement: Supplementary file 2 [file pone.0354012.s004.pdf]

Response to Reviewers

Manuscript PONE-D-26-32102R1

FedMamba-ChebKAN: Federated Mamba with Chebyshev-KAN for Heterogeneous IIoT Fault Detection

Dear Dr. Saif,

Thank you for the opportunity to submit a further revision, and please pass our thanks to both reviewers for their careful reading of the R1 manuscript and for confirming that all previous comments have been addressed. This revision responds to the three remaining points: (1) Reviewer 1's remark on data and code availability, (2) the legibility of Figures 7 and 10, and (3) the spacing artefacts noted in the Abstract. Every change is marked in the tracked-changes copy; the reviewer text is reproduced below in full, followed by our response and the corresponding change.

Reviewer #1

**Comment 1.1**	*"The authors have made a serious effort to address the concerns raised during the previous review round. The revised manuscript is substantially improved in terms of methodological clarity, statistical reporting, literature positioning, figure consistency, and discussion of limitations. […] The statistical analysis appears appropriately documented, with multiple independent runs, Wilcoxon signed-rank testing, effect-size reporting, and clearly defined evaluation metrics. The discussion of privacy guarantees, convergence limitations, and deployment constraints has been strengthened and is presented in a balanced manner."*
**Response**	We thank the reviewer for this assessment. No change was required in response to this comment.
**Comment 1.2**	*"One issue remains regarding reproducibility and data availability. Although all benchmark datasets are publicly accessible, the manuscript does not provide the underlying experimental outputs, source code, trained models, or partition files used to generate the reported results. Therefore, full availability of all data underlying the findings is not yet achieved according to the strictest interpretation of reproducibility requirements."*
**Response**	We thank the reviewer for distinguishing this point from the scientific validity of the work. All four benchmark datasets (CWRU, Paderborn, MIMII, NASA C-MAPSS) are publicly available from their original repositories, and the Data Availability statement gives the exact download locations; no new data were generated in this study, so all data underlying the findings are fully accessible without restriction.<br><br>With respect to experimental outputs, source code, trained models, and partition files: the manuscript already contains everything required to reproduce the reported results independently. The architecture is fully specified (Methods), the aggregation rule is given as Algorithm 1 with its DP mechanism, every evaluation metric is defined in closed form, the complete hyperparameter set is listed in the Experimental setup ($T = 200$, $E = 2$, $C = 0.3$, $d_m = 64$, $N_{layers} = 3$, $d_{state} = 16$, $d_{conv} = 4$, $p = 8$, KAN dims [64,32], $N = 8$, $\tau = 10^{-3}$, $\sigma = S = \gamma = 1.0$, $K = 12/20$), the non-IID partition protocol is described in the Datasets section together with the Dirichlet α levels, and the number of seeds, the statistical tests, and the effect-size

	measure are all stated. We therefore respectfully maintain the current data statement, which we believe satisfies the PLOS data policy for a study based entirely on public benchmark datasets. We are, of course, happy to respond to specific reproducibility questions from the editor.<br>Changes: none to the manuscript for this point.

Reviewer #2

**Comment 2.1**	*"The authors have done an excellent job addressing the concerns raised in the previous round of review. The revised manuscript […] is significantly improved. I recommend this manuscript for Acceptance. Below are a few minor, simple comments for the authors to consider for final polishing before publication."*
**Response**	We are grateful for the recommendation and have addressed both polishing items below.
**Comment 2.2**	*"Please do a final visual check of Figure 7 and Figure 10 to ensure all axis labels, legends, and annotations are perfectly legible in the final high-resolution export."*
**Response**	We inspected both figures at full resolution and found two genuine legibility problems, which are now fixed.<br>Figure 7 (non-IID robustness). The horizontal-axis tick labels for $\alpha = 0.05$ and $\alpha = 0.10$ overlapped (the "(extreme)"/"(severe)" descriptors collided with the "(mod.)" label of $\alpha = 0.30$). The tick labels have been re-laid-out on separate lines so that no label overlaps; the plotted data, legend, and Zhou et al. marker are unchanged. The caption was also corrected: it previously referred to "±1σ shaded" bands that the figure does not draw; it now lists the α levels shown on the axis.<br>Figure 10 (ablation). The six rotated multi-line configuration names on the x-axis of each panel were too small to read, and the C-MAPSS panel used an inverted axis with bars hanging from the top, which several readers found confusing. The figure has been regenerated from Table 7 with (i) short configuration letters A–F on the axis and a full-text key beneath the panels, (ii) conventional upward bars with ±1σ error bars in all three panels, (iii) the numerical value printed above each bar, and (iv) larger, non-overlapping Δ annotations on the CWRU panel. All values are identical to Table 7. The caption now defines configurations A–F explicitly and maps them to the rows of Table 7.<br>Both figures are exported at ≥300 dpi and have been checked against the PLOS figure specifications.
**Comment 2.3**	*"In the Abstract, ensure consistent spacing (e.g., "p rovidin g" and "freque ncy" appear to have minor spacing artifacts in the provided text draft, likely from PDF extraction)."*
**Response**	We checked the LaTeX source and the compiled PDF: the words "providing" and "frequency" contain no stray spaces in the source, and text extracted from our compiled PDF reads correctly. As the reviewer suspects, the artefacts arose from

	text extraction of the review PDF (character-spacing adjustments from the microtype package, which the PLOS template enables, are occasionally mis-parsed by extractors). To eliminate the possibility that the artefact recurs in the production PDF, we have also disabled microtype expansion/tracking for the Abstract in the revised source. No wording in the Abstract has changed.

Additional changes

Beyond the points above, Figure 1 (system architecture) has been redrawn as a vector figure so that all tier labels, module names, and the three SpectralFedAvg steps are legible at print size; its content is unchanged. While checking the Abstract for the spacing issue we also made a few minor wording refinements in the Abstract and Conclusion (e.g. "integrates" → "couples", "satisfying" → "respecting"); these are stylistic only and visible in the tracked-changes copy. No result, number, or reference was changed in this revision. The reference list is unchanged and contains no retracted articles. The author list and order in Editorial Manager have been checked against the manuscript byline.

We hope the manuscript is now suitable for publication in PLOS ONE and thank the editor and reviewers again for their time.
Yours sincerely,

Mohammed Tawfik (on behalf of all authors)
