# Supplementary material for: FedMamba-ChebKAN: Federated Mamba with Chebyshev-KAN for heterogeneous IIoT fault detection
Source: PLoS One. 2026 Nov 11;21(11):e0354012. doi: 10.1371/journal.pone.0354012 (PMC13754226; doi:10.1371/journal.pone.0354012)
Supplement: Supplementary file 3 [file pone.0354012.s005.pdf]

Response to Reviewers

Manuscript PONE-D-26-32102R2

FedMamba-ChebKAN: Federated Mamba with Chebyshev-KAN for Heterogeneous IIoT Fault Detection

Dear Dr. Saif,

Thank you for the opportunity to revise the manuscript, and please thank both reviewers. Reviewer 1 asked for the data and code underlying the results, and Reviewer 3 raised eight points on privacy, baselines, data splitting, consistency, statistics, theory, reproducibility and presentation. Several of these points could only be answered by new experiments, so we re-implemented the framework and obtained every result in this revision from new runs under a corrected protocol. The main changes are:

(1) Every split is now made on recordings, recording segments or engines before windowing, with normalisation fitted on training data only; C-MAPSS is evaluated on the official test engines, one prediction per engine.

(2) FedAvg, FedProx, SCAFFOLD and FedBN now use exactly the same Mamba + Chebyshev-KAN model, partitions, seeds and training budget as SpectralFedAvg; the 1-D CNN result has been removed.

(3) The privacy mechanism is replaced: every client now runs DP-SGD, so the guarantee holds against the coordinator and covers the truncation degrees; ε is computed with a validated accountant and the accuracy cost is measured.

(4) Proposition 1 is replaced by a bound that covers heterogeneous aggregation and makes no convergence-rate claim.

(5) Statistics use exact Wilcoxon tests, correctly signed rank-biserial effect sizes and Benjamini–Hochberg correction.

(6) All result figures are generated from the per-seed result files; Figure 1 is redrawn.

(7) The code, client partitions, configuration and per-seed results are public on GitHub and provided as S1 Data and S2 File.

Every table and figure in this revision is produced by the released code from the released per-seed result files, so each number can be checked independently; these results replace all values of the previous version. The new results are more modest and change the conclusions: the clearest gains come from keeping BatchNorm on the clients, and the manuscript now says so.

Every change is marked in the tracked-changes copy. The reviewer text is reproduced below, followed by our response and the corresponding change.

Reviewer #1 (Dr Muhammad Ali Abid)

**Comment 1.1**	*"The authors have responded comprehensively to the comments raised during the previous review rounds, and the manuscript has improved significantly […] Subject to the journal's policy regarding data and code availability, I believe the manuscript is suitable for publication."*
**Response**	We thank the reviewer for this assessment and for the recommendation.

**Comment 1.2**	*"One remaining issue concerns data availability and reproducibility. Although all benchmark datasets are publicly available and properly referenced, the manuscript*

	does not provide source code, trained models, partition files, or the underlying experimental outputs used to generate the reported results. […]"
**Response**	We agree and have released everything needed to reproduce the results. The full implementation (preprocessing, partition generation, models, federated training, the private variant, explainability and analysis), the dependency versions and the client-partition manifests for every dataset are available at https://github.com/kmkholm/FedMamba-ChebKAN. The per-seed results of every reported run are provided as S1 Data, and the client partitions and the configuration of every run as S2 File; trained models can be regenerated with the released code and seeds.<br>Every table and figure in the revised manuscript is computed by the released analysis code from these per-seed results.
**Changes**	New Supporting information (S1 Data, S2 File); Data availability statement rewritten with the repository.

Reviewer #3

**Comment 3.0**	*"[…] several issues currently affect the validity of the privacy guarantee, fairness of the comparisons, and reliability of the reported results. I therefore recommend major revision."*
**Response**	We thank the reviewer for a careful reading. We agreed with the substance of every point; because several of them concern the experiments themselves, we re-implemented the framework and obtained all results from new runs, as described below.

**Comment 3.1**	*"The differential-privacy guarantee requires fundamental correction. […] The authors should correct the mechanism, clarify the threat model, and recalculate the privacy budget using a validated accountant. Otherwise, the claims of formal differential privacy and protection against an honest-but-curious coordinator should be removed or substantially narrowed."*
**Response**	We agree with the analysis: with joint clipping of the raw update, giving high-order coefficients less noise weakens the guarantee, because the least-perturbed coordinate governs the privacy loss; the transmitted degrees were unprotected; and noise added by the coordinator cannot protect clients against that coordinator. We therefore corrected the mechanism and the threat model, recalculated the budget, and re-measured the accuracy cost.<br>(a) Mechanism. The coordinator-side perturbation has been replaced by example-level DP-SGD inside every client (Abadi et al., 2016): per-example gradients are clipped to norm $C = 1$, Gaussian noise $N(0, \sigma^2 C^2 I)$ is added on the client, and Adam uses the noisy gradient. Everything a client uploads, including the Chebyshev coefficients and the truncation degrees, is computed from this noisy model, so the degrees are covered by post-processing. BatchNorm is replaced by GroupNorm, windows are standardised individually, and the last-round model is reported, so that no statistic or model selection uses private data outside the mechanism.<br>(b) Threat model. Because the noise is added before anything leaves the client, the guarantee holds against the coordinator, the gateways and the other clients; it protects every individual training window. The claim of client-level protection has been removed; the Discussion explains why a client-level guarantee with 12 clients is

	not realistic (the Rényi accountant gives ε ≈ 24.5 already at σ = 1 for q = 0.3 and T = 100) and what it would require (secure aggregation, larger fleets; matched clipping for order-scaled noise).<br>(c) Accounting. ε is computed per client with the Rényi-DP accountant of Google's dp_accounting library (v0.6.0) from the number of noisy steps each client actually took, at δ = 1e-5, and the largest value over the clients is reported.<br>(d) Results (CWRU, seeds 1–3; encoder pretrained on public Paderborn data from operating settings not used in the benchmark; every client uses all non-overlapping windows of its training segments): 92.5 ± 1.2 % without noise, 92.4 ± 0.1 % at ε = 19.1, 89.5 ± 0.5 % at ε = 8.1 and 85.6 ± 1.1 % at ε = 3.9 (new Table 8). At ε ≈ 19 the private model is within 0.1 points of the same model without noise; the Discussion ("Privacy: scope and implications") states the scope of the guarantee.
**Changes**	Abstract; keywords; contributions; Related work and Table 1; Methods ("Example-level differential privacy", Algorithm 1); Results ("Privacy–utility trade-off", Table 8); Discussion ("Privacy: scope and implications"); Conclusion; new references (Abadi et al. 2016 is now cited for DP-SGD; Wu and He 2020; Hölzl et al. 2023; Mohammadi et al. 2026).

**Comment 3.2**	*"The baseline comparison does not clearly separate the contribution of the model architecture from that of SpectralFedAvg. […] FedAvg, FedProx, SCAFFOLD, FedBN, and SpectralFedAvg should be compared using the same Mamba plus Cheb-KAN architecture, client partitions, seeds, and tuning budget. The recent FedMamba study […] should also be discussed […] a Mamba plus FedProx baseline should be implemented under the present protocol."*
**Response**	We agree and have done exactly this. FedAvg, FedProx, SCAFFOLD, FedBN and SpectralFedAvg now use the same Mamba + Chebyshev-KAN model (126,592 parameters on the vibration data), the same client partitions, the same eight seeds and the same optimiser settings and round budget, so their differences isolate the aggregation rule. The FedProx run is therefore the Mamba + FedProx baseline requested by the reviewer. The 1-D CNN FedAvg result has been removed, and we added SpectralFedAvg-BN, which keeps BatchNorm on the clients.<br>Results (mean ± s.d., 8 seeds). CWRU accuracy: FedAvg 85.7 ± 1.0 %, FedProx 87.2 ± 1.6 %, SCAFFOLD 67.1 ± 2.5 %, FedBN 98.0 ± 0.8 %, SpectralFedAvg 85.5 ± 0.9 %, SpectralFedAvg-BN 98.0 ± 0.5 %, pooled reference 98.7 ± 0.2 %. Paderborn: all methods except SCAFFOLD 97.9–98.4 %. MIMII AUROC: 0.800–0.805 for all methods except SCAFFOLD. C-MAPSS RMSE: FedAvg 20.89, FedProx 20.75, FedBN 20.78, SpectralFedAvg 20.41, SpectralFedAvg-BN 20.29 ± 0.51, pooled reference 19.98.<br>With the architecture held fixed, the aggregation rule alone changes accuracy only slightly; the large gain on CWRU comes from client-local normalisation, which SpectralFedAvg-BN shares with FedBN. The manuscript now states this directly, and the component analysis (configurations C, E and E-BN, Table 7 and Fig 7) separates the aggregation rule from client-local normalisation. SCAFFOLD, which was designed for SGD local steps, was unstable with the Adam optimiser shared by all methods; we report this as a limitation rather than tuning it separately.<br>We thank the reviewer for the FedMamba reference. Ahmad et al. (Internet of Things 36 (2026) 101877) is now discussed in the Introduction and Related work and added to Table 1; as the reviewer notes, its task and data differ from ours, so its published numbers are not compared.
**Changes**	Methods ("Federated training"); Tables 3 and 4; Fig 2; component analysis (Table 7,

	Fig 7); Discussion; Limitations; Introduction, Related work and Table 1 (FedMamba); new reference.

**Comment 3.3**	*"The data-splitting procedure may permit leakage. […] The authors should split recordings, bearing units, or engines first, generate windows afterward, and fit all normalization parameters using training data only. Exact split ratios, sample counts, class distributions, and client assignments should be reported. The relationship between the physical client partitions and the Dirichlet partitions must also be clarified."*
**Response**	We agree and have rebuilt all partitions so that every split is made before windowing and all normalisation statistics are fitted on each client's training data only.<br>CWRU: 12 clients = 4 loads × 3 accelerometer positions; each recording is cut into contiguous 60/20/20 % segments before windowing (1024 samples, 50 % overlap only inside a segment); 65 training and 40 validation and 40 test windows per class and client (2860 / 1760 / 1760 windows in total). The normal recordings have no base-accelerometer channel, so the base clients hold no normal windows.<br>Paderborn: 12 clients = 2 operating settings × 6 bearing triples (one healthy, one outer-race and one inner-race bearing with real damage); the 20 recordings of each bearing and setting are dealt to its clients without overlap and split 4/1/1 by recording; 219 training windows per client (73 per class), 120 validation and 120 test windows. The clients share the three classes while differing in bearing devices and operating condition.<br>MIMII: 12 clients = 4 machine IDs × 3 SNR levels; split by clip; 215 normal training clips, 40 normal validation clips, and 100 normal plus 100 abnormal test clips per client.<br>C-MAPSS: 20 clients = 5 groups of training engines per FD subset; one engine in ten per client held out for validation; testing on the official test engines (707) with one prediction per engine.<br>The client-to-recording, client-to-clip and client-to-engine assignments, class counts and sample counts are given in S2 File and the repository. The Dirichlet study has been removed, so all results use the physical partitions above.
**Changes**	Methods: dataset paragraph and Table 2 rewritten; S2 File; non-IID Dirichlet figure removed.

**Comment 3.4**	*"Several evaluation procedures and numerical results are inconsistent. […] These inconsistencies should be corrected using the underlying per-seed results."*
**Response**	C-MAPSS macro-AUROC: removed; no classification target is defined for C-MAPSS.<br>PHM08 score: now computed per engine on the official test set (last window of each engine against the official RUL), per FD subset and averaged over the four subsets.<br>Noise: the main comparison is run without noise for every method, and the text now says so; noise is used only in the privacy study.<br>Rounds to target: removed; convergence is shown by the logged test metric of every run (Fig 5). On CWRU the two methods with client-local BatchNorm pass 95 % accuracy within 30–35 rounds, whereas the methods that average normalisation statistics level off near 83–85 %.<br>Bandwidth: the upload is now measured from the transmitted tensors (including the bytes saved by truncation and the one-byte degrees). Per participating client and round, FedAvg uploads 506 kB, SpectralFedAvg 502 kB, SpectralFedAvg-BN 501 kB

	and SCAFFOLD 1013 kB; the nominal fleet uplink over 100 rounds is about 202 MB for FedAvg. The previous bandwidth-reduction claim has been removed: at this model size truncation saves less than 1 % of the upload.<br>All tables are generated from the per-seed result files (S1 Data).
**Changes**	Table 4; Methods (C-MAPSS protocol, training settings); Results (convergence and communication); Fig 5, Fig 6; Table 6.

**Comment 3.5**	*“The statistical analysis should be revised. Eight random seeds do not automatically make a Wilcoxon test adequately powered. […] define primary outcomes or apply an appropriate correction. The signs of the rank-biserial correlations should also be checked. Finally, the centralized model should be described as a pooled-data reference […]”*
**Response**	Power: the statement that the test is properly powered has been removed; the Methods explain that with eight seeds the smallest two-sided p is 0.0078, so only consistent differences can be detected, and that a non-significant result is not evidence of equivalence.<br>Multiplicity: Benjamini–Hochberg adjustment is applied over the 32 comparisons with federated baselines (eight metrics across the four datasets × four baselines) and, separately, the eight comparisons with the pooled reference; raw p and adjusted q are reported.<br>Exact test and effect size: p values are exact (enumeration of all 2^8 sign patterns, valid with ties), and $r_{rb} = (W+ - W-)/(W+ + W-)$ is oriented so that a positive value favours the proposed method, which fixes the sign problem.<br>The centralised model is called the pooled-data reference throughout.<br>Results (Table 5, SpectralFedAvg-BN): on CWRU it is better than FedAvg, FedProx and SCAFFOLD in all eight seeds ($p = 0.0078$, $q = 0.023$, $r_{rb} = +1$) and indistinguishable from FedBN ($p = 1.0$); on C-MAPSS its RMSE is lower than that of FedAvg ($q = 0.039$) but not significantly different from FedProx ($q = 0.083$) or FedBN ($q = 0.18$); on Paderborn and MIMII most differences are not significant. It remains below the pooled reference on CWRU and Paderborn. The text reports these non-significant results as such.
**Changes**	Methods (“Statistical tests”); Results (“Statistical significance”); Table 5.

**Comment 3.6**	*“The theoretical proposition does not appear to match the actual aggregation procedure. […]”*
**Response**	We agree with all three observations and replaced Proposition 1. It now bounds, for one edge and one round, the truncation error of each client edge function directly from the tail-energy criterion, $\|\phi^{(k)} - \tilde{\phi}^{(k)}\|_\infty \le \sqrt{N - N^{(k)}}\,\sqrt{\tau}\,\|c^{(k)}\|_2$, and the error between the aggregated function and each client function as the weighted truncation error plus a client-heterogeneity term Γ_k that is present for any averaging rule. The text after the proof states that zero-padding does not recover discarded coefficients. The Jackson and Bernstein theorems are cited only to explain coefficient decay, and every description of an exponential convergence rate has been removed.
**Changes**	Methods: “Error of the aggregated edge function” (Proposition 1, proof, interpretation and scope of the bound); Abstract; contributions.

**Comment 3.7**	*“The reproducibility materials are insufficient. […] The authors should release the*

	implementation, dependency versions, preprocessing scripts, split and client-assignment files, experiment configurations, privacy-accountant code, and data supporting all reported results. Pseudocode alone is insufficient to reproduce the proposed method."
**Response**	All of these are now released (see our response to Reviewer 1, comment 1.2): implementation, requirements file with versions, preprocessing and partition code, partition manifests, run configurations, the DP-SGD implementation, the public-pretraining script and the privacy-accountant code, at https://github.com/kmkholm/FedMamba-ChebKAN; the per-seed results of every reported run are provided as S1 Data and the client partitions and run configuration as S2 File.
**Changes**	Data availability; S1 Data; S2 File.

**Comment 3.8**	*"The presentation and interpretation claims should be moderated. The figures are difficult to read and should be regenerated at publication quality […] Figure 12 should be cited and explained or removed. Learned Mamba dimensions should not be labelled as specific physical quantities […] the Abstract should be reduced to the journal's 300-word limit."*
**Response**	Figures: Figure 1 is redrawn as an editable vector diagram (system, local model, one SpectralFedAvg round and three component details). All result figures are generated from the per-seed result files with one font and consistent colours and exported as 300-dpi TIFF files.<br>Removed: the radar chart (former Fig 12), the KAN activation grid (former Fig 11; its feature labels were not validated), the significance figure, and the Dirichlet figure (study not repeated).<br>Claims: mechanical-interpretability and governance-compliance statements removed, and the privacy claims narrowed to the example-level guarantee that is measured; the Limitations state that no mapping between encoder features and physical quantities was validated; "first" claims removed; promotional wording replaced.<br>Explainability: two new figures computed from the trained model on real CWRU test windows replace the former activation grid: integrated-gradients attribution and frequency-band occlusion (Fig 8), and t-SNE embeddings, the Chebyshev coefficient tail energy and the learned output-edge functions (Fig 9). No physical label is attached to an encoder feature; the one physical statement, the 9.4 ms spacing of the outer-race impacts (106 Hz defect frequency), is measured on the input signal.<br>Abstract: rewritten and shortened to 272 words.
**Changes**	Figures 1–9 (Figs 8 and 9 new, Results: "Explainability"); Abstract; Results; Discussion; Conclusion.

Figure numbering in this revision

Fig 1 — overview (replaces former Figs 1 and 2)

Fig 2 — performance on the four benchmarks

Fig 3 — confusion matrices (CWRU, Paderborn)

Fig 4 — C-MAPSS RUL prediction

Fig 5 — convergence

Fig 6 — communication cost

Fig 7 — aggregation and normalisation analysis

Fig 8 — attribution on real test windows (new)

Fig 9 — embeddings and Chebyshev-KAN head (new)

Removed — former Fig 6 (significance), Fig 7 (Dirichlet), Fig 11 (KAN activations, replaced by Fig 9c), Fig 12 (radar chart)

Additional changes

Training settings of the new runs: T = 100 rounds, patch length 16 for the 1024-sample vibration windows, batch 256 for C-MAPSS, and Chebyshev coefficients initialised with amplitude decaying as $(1+n)^{-2}$; all are stated in the Methods and the configuration files. A client-local normalisation variant (SpectralFedAvg-BN) is reported alongside SpectralFedAvg. The hyperparameter-sensitivity table and the Dirichlet study were not repeated and have been removed. The Discussion and Conclusion were rewritten to match the new results, and the Limitations now cover the shared optimiser (SCAFFOLD), the absolute C-MAPSS error and the model size. References were added for the new material: Ahmad et al. (2026), requested by Reviewer 3, Benjamini and Hochberg (1995), and the DP-SGD, normalisation and explainability references cited in the text. The reference list contains no retracted articles.

We hope the revised manuscript is now suitable for publication in PLOS ONE, and we thank the editor and reviewers again for their time.

Yours sincerely,

Mohammed Tawfik (on behalf of all authors)
