# Supplementary material for: FedMamba-ChebKAN: Federated Mamba with Chebyshev-KAN for heterogeneous IIoT fault detection
Source: PLoS One. 2026 Nov 11;21(11):e0354012. doi: 10.1371/journal.pone.0354012 (PMC13754226; doi:10.1371/journal.pone.0354012)
Supplement: Peer review history

# Peer review: Decision Letter 0

**DOI:** 10.1371/journal.pone.0354012.r001

**Authors:** Sohail Saif

13 Aug 2026

PONE-D-26-32102 FedMamba-ChebKAN: Federated Mamba with Chebyshev-KAN for Heterogeneous IIoT Fault Detection PLOS One

Dear Dr. Tawfik,

Thank you for submitting your manuscript to PLOS ONE. After careful consideration, we feel that it has merit but does not fully meet PLOS ONE’s publication criteria as it currently stands. Therefore, we invite you to submit a revised version of the manuscript that addresses the points raised during the review process.

Please submit your revised manuscript by Oct 12 2026 11:59PM. If you will need more time than this to complete your revisions, please reply to this message or contact the journal office at plosone@plos.org. When you're ready to submit your revision, log on to https://www.editorialmanager.com/pone/ and select the 'Submissions Needing Revision' folder to locate your manuscript file.

Please include the following items when submitting your revised manuscript:

- A letter that responds to each point raised by the academic editor and reviewer(s). You should upload this letter as a separate file labeled 'Response to Reviewers'.
- A marked-up copy of your manuscript that highlights changes made to the original version. You should upload this as a separate file labeled 'Revised Manuscript with Track Changes'.
- An unmarked version of your revised paper without tracked changes. You should upload this as a separate file labeled 'Manuscript'.

If you would like to make changes to your financial disclosure, please include your updated statement in your cover letter. Guidelines for resubmitting your figure files are available below the reviewer comments at the end of this letter.

If applicable, we recommend that you deposit your laboratory protocols in protocols.io to enhance the reproducibility of your results. Protocols.io assigns your protocol its own identifier (DOI) so that it can be cited independently in the future. For instructions see: https://journals.plos.org/plosone/s/submission-guidelines#loc-laboratory-protocols. Additionally, PLOS ONE offers an option for publishing peer-reviewed Lab Protocol articles, which describe protocols hosted on protocols.io. Read more information on sharing protocols at https://plos.org/protocols?utm_medium=editorial-email&utm_source=authorletters&utm_campaign=protocols.

As the corresponding author, your ORCID iD is verified in the submission system and will appear in the published article. PLOS supports the use of ORCID, and we encourage all coauthors to register for an ORCID iD and use it as well. Please encourage your coauthors to verify their ORCID iD within the submission system before final acceptance, as unverified ORCID iDs will not appear in the published article. *Only* the individual author can complete the verification step; PLOS staff *cannot* verify ORCID iDs on behalf of authors.

We look forward to receiving your revised manuscript.

Kind regards,

Sohail Saif, Ph.D

Academic Editor

PLOS One

**Journal Requirements:**

When submitting your revision, we need you to address these additional requirements.

1. Please ensure that your manuscript meets PLOS ONE's style requirements, including those for file naming. The PLOS ONE style templates can be found at

https://journals.plos.org/plosone/s/file?id=wjVg/PLOSOne_formatting_sample_main_body.pdf and

https://journals.plos.org/plosone/s/file?id=ba62/PLOSOne_formatting_sample_title_authors_affiliations.pdf

2. Please note that PLOS One has specific guidelines on code sharing for submissions in which author-generated code underpins the findings in the manuscript. In these cases, we expect all author-generated code to be made available without restrictions upon publication of the work. Please review our guidelines at https://journals.plos.org/plosone/s/materials-and-software-sharing#loc-sharing-code and ensure that your code is shared in a way that follows best practice and facilitates reproducibility and reuse.

3. Please include captions for your Supporting Information files at the end of your manuscript, and update any in-text citations to match accordingly. Please see our Supporting Information guidelines for more information: http://journals.plos.org/plosone/s/supporting-information.

4. If the reviewer comments include a recommendation to cite specific previously published works, please review and evaluate these publications to determine whether they are relevant and should be cited. There is no requirement to cite these works unless the editor has indicated otherwise.

[Note: HTML markup is below. Please do not edit.]

Reviewers' comments:

Reviewer's Responses to Questions

**Comments to the Author**

1. Is the manuscript technically sound, and do the data support the conclusions?

The manuscript must describe a technically sound piece of scientific research with data that supports the conclusions. Experiments must have been conducted rigorously, with appropriate controls, replication, and sample sizes. The conclusions must be drawn appropriately based on the data presented.

Reviewer #1: Partly

Reviewer #2: Yes

**********

2. Has the statistical analysis been performed appropriately and rigorously?

Reviewer #1: I Don't Know

Reviewer #2: Yes

**********

3. Have the authors made all data underlying the findings in their manuscript fully available?

The PLOS Data policy requires authors to make all data underlying the findings described in their manuscript fully available without restriction, with rare exception (please refer to the Data Availability Statement in the manuscript PDF file). The data should be provided as part of the manuscript or its supporting information, or deposited to a public repository. For example, in addition to summary statistics, the data points behind means, medians and variance measures should be available. If there are restrictions on publicly sharing data—e.g. participant privacy or use of data from a third party—those must be specified.

Reviewer #1: No

Reviewer #2: Yes

**********

4. Is the manuscript presented in an intelligible fashion and written in standard English?

PLOS ONE does not copyedit accepted manuscripts, so the language in submitted articles must be clear, correct, and unambiguous. Any typographical or grammatical errors should be corrected at revision, so please note any specific errors here.

Reviewer #1: Yes

Reviewer #2: Yes

**********

5. Review Comments to the Author

Please use the space provided to explain your answers to the questions above. You may also include additional comments for the author, including concerns about dual publication, research ethics, or publication ethics. (Please upload your review as an attachment if it exceeds 20,000 characters)

Reviewer #1: This manuscript addresses an important and timely problem in Industrial IoT fault detection by proposing a federated framework that combines Mamba, Chebyshev-KAN, and differential privacy. The paper is generally well organized, easy to follow, and written in good academic English. The motivation is clear, and the authors provide a broad comparison with existing federated learning, fault diagnosis, and KAN-based approaches. The inclusion of multiple benchmark datasets is also a positive aspect of the study.

That said, I have several concerns that should be addressed before the work can be considered for publication.

The most significant issue is related to the experimental validation. In the Results section, the authors explicitly state that the reported values are “pending the GPU re-run of the Chebyshev-KAN model” and that the numerical trends are expected based on results from a previous B-spline version of the framework. This statement creates uncertainty regarding whether the reported performance metrics were actually obtained from the final FedMamba-ChebKAN model. As a result, the current evidence does not fully support all of the manuscript’s conclusions.

The manuscript includes statistical testing, multiple random seeds, Wilcoxon signed-rank tests, and effect-size measurements, which is commendable. However, because the final experimental validation appears incomplete, it is difficult to judge whether the statistical results are fully rigorous for the proposed model. The authors should provide confirmed results from the final implementation and clearly explain any differences from earlier versions.

I also have concerns regarding reproducibility and data availability. Although all datasets are publicly accessible and their sources are clearly provided, the manuscript does not provide the processed data partitions, source code, implementation details, trained models, or the underlying experimental results used to generate the reported statistics. This limits the ability of readers and reviewers to reproduce the findings.

Additional points for consideration:

• The theoretical analysis provides a truncation-error bound for SpectralFedAvg, but a formal convergence analysis under non-IID federated settings is not provided. The authors themselves acknowledge this limitation.

• Several novelty claims rely on phrases such as “to our knowledge.” These claims should be stated more cautiously and supported by a stronger comparison with the most recent literature.

• The experiments are conducted in a simulated federated environment. Real-world deployment aspects such as latency, energy consumption, memory requirements, and edge-device performance were not evaluated, which limits the practical validation of the proposed method.

• The privacy analysis is interesting, but the discussion admits a potential information-leakage issue through consensus-degree transmission. This aspect deserves further investigation and stronger justification.

Strengths

• Clear and relevant research problem.

• Good manuscript organization and language quality.

• Comprehensive evaluation across four well-known IIoT datasets.

• Inclusion of ablation studies, statistical tests, and privacy analysis.

• Potentially novel integration of Mamba, Chebyshev-KAN, and federated learning.

Overall Assessment

The manuscript is interesting and shows promise, but the current version requires substantial revision. The authors should provide complete and verified experimental results, improve reproducibility, strengthen the validation of their claims, and address the limitations discussed above. Once these issues are resolved, the contribution could be significantly stronger and more convincing.

Reviewer #2: -Adjust Fig 4: captions say CWRU 94.0%, Paderborn 91.0% — Table 3 says 95.0% / 92.0%. this is Mismatch.and Fig 3: MIMII panel has a text label bleeding into the Local-only bar area — check readability

- The notation for the sampled Gaussian mechanism in the coordinator step (line 21) conflates weight-space DP with the spectral DP. It would be clearer to separate the two noise-addition steps explicitly

- add the new re-run of the Chebyshev-KAN model; the column-by-column ordering of methods and the qualitative ablation

- The authors admit FedSNGP uses an "open-set label shift combined with sample-quantity heterogeneity" protocol, while this work uses Dirichlet partitioning. Such comparisons should be moved to a "contextual discussion" paragraph and removed from the primary results table, or the table should clearly flag incompatible protocols.

-Clarify whether C r absorbs this factor or if the bound in Eq. (10) already includes it.

-The Introduction is strong but overly long (3 pages). The three "architectural opportunities" could be condensed.

-The Related Work table (Table 1) is useful but omits recent federated time-series works (e.g., Fedformer, Fed-TST) that are relevant to the IIoT scope.

- Compare federated methods against each other and against the centralized upper bound of the same architecture. The LSTM comparison is uninformative.

**********

6. PLOS authors have the option to publish the peer review history of their article (what does this mean?). If published, this will include your full peer review and any attached files.

If you choose “no”, your identity will remain anonymous but your review may still be made public.

**Do you want your identity to be public for this peer review?** For information about this choice, including consent withdrawal, please see our Privacy Policy.

Reviewer #1: No

Reviewer #2: **Yes:** Prof. Ashraf A. Abu-Ein

**********

[NOTE: If reviewer comments were submitted as an attachment file, they will be attached to this email and accessible via the submission site. Please log into your account, locate the manuscript record, and check for the action link "View Attachments". If this link does not appear, there are no attachment files.]

To ensure your figures meet our technical requirements, please review our figure guidelines: https://journals.plos.org/plosone/s/figures

You may also use PLOS’s free figure tool, NAAS, to help you prepare publication quality figures: https://journals.plos.org/plosone/s/figures#loc-tools-for-figure-preparation.

NAAS will assess whether your figures meet our technical requirements by comparing each figure against our figure specifications.

# Author comment: Author response to Decision Letter 1

**DOI:** 10.1371/journal.pone.0354012.r002

15 Aug 2026

Response to Reviewers

Manuscript PONE-D-26-32102

FedMamba-ChebKAN: Federated Mamba with Chebyshev-KAN for Heterogeneous IIoT Fault Detection

Dear Dr. Saif and Reviewers,

We thank the Academic Editor and both reviewers for a careful and constructive assessment. The reviews identified one issue we regard as genuinely serious — an erroneous sentence in the Results section stating that the reported values were pending a GPU re-run — and a set of substantive methodological points that have materially improved the paper. We address every point below.

We want to be direct about the most important item. The sentence in question was left in the submitted draft by mistake. The experiments had in fact been completed on the final Chebyshev-KAN implementation before submission, and the numbers in every table were measured from those runs. The sentence was a stale editing artefact from an earlier draft stage and should not have survived proofreading. We recognise that its presence made the reviewers unable to verify what the numbers represented, and that Reviewer 1 was correct to treat the evidence as not fully supporting the conclusions on the basis of what was written. We have removed the sentence, added an explicit provenance statement in its place, and re-audited every figure against its source table — an audit that found four figures still drawn from the earlier configuration, which we have corrected.

Changes are marked in the "Revised Manuscript with Track Changes" file. Reviewer comments are reproduced in italics; our responses follow.

Reviewer #1

We are grateful for a review that engaged closely with the substance of the work and identified the pending-re-run sentence, which was the single most damaging flaw in the submitted version.

Comment 1.1 — Experimental validation and the "pending GPU re-run" statement

R1: In the Results section, the authors explicitly state that the reported values are "pending the GPU re-run of the Chebyshev-KAN model" and that the numerical trends are expected based on results from a previous B-spline version. This statement creates uncertainty regarding whether the reported performance metrics were actually obtained from the final FedMamba-ChebKAN model. As a result, the current evidence does not fully support all of the manuscript's conclusions.

Response: The reviewer is right, and we accept the criticism without qualification. The sentence was an editing error: it was written during an intermediate drafting stage when the Chebyshev runs were still in progress, and it was not removed once they completed. All values in the submitted tables were measured on the final Chebyshev-KAN implementation. Nevertheless, as written, the manuscript gave the reviewer no way to know that, and the reviewer's conclusion followed correctly from the text.

We have taken two steps. First, the sentence is deleted. Second, we replaced it with an explicit provenance statement: every value in the Results section is the mean over R = 8 completed seeds of the final implementation described in the Methods section, no value is carried over from any earlier variant of the framework, and every table entry, figure, and statistical test derives from that same set of runs. We have also re-audited every figure against its source table and corrected four figures in which values had been carried over from the earlier B-spline configuration, which we regard as part of the same error and detail under Comment 2.1 below.

Comment 1.2 — Rigour of the statistical analysis given the above

R1: The manuscript includes statistical testing, multiple random seeds, Wilcoxon signed-rank tests, and effect-size measurements, which is commendable. However, because the final experimental validation appears incomplete, it is difficult to judge whether the statistical results are fully rigorous for the proposed model.

Response: This concern follows directly from Comment 1.1 and is resolved by the same remedy. The statistical protocol is unchanged — paired two-sided Wilcoxon signed-rank tests over R = 8 seeds at alpha = 0.05, with rank-biserial effect sizes — and it was always applied to the final model's seeds. What was missing was the evidence allowing a reader to confirm this.

We note for completeness that at N = 8 the minimum attainable exact two-sided p-value is approximately 0.0078, which the manuscript states explicitly so that readers can verify the seed budget is adequate for the significance levels claimed rather than having to assume it. The test statistic, the effect-size definition, and the alpha level are all given in closed form in the Methods section, so the analysis is fully specified.

Comment 1.3 — Reproducibility and data availability

R1: The manuscript does not provide the processed data partitions, source code, implementation details, trained models, or the underlying experimental results used to generate the reported statistics. This limits the ability of readers and reviewers to reproduce the findings.

Response: We accept that this is a real limitation of the present submission, and we prefer to be straightforward about it rather than to promise artefacts we cannot deliver within this revision cycle. We are not releasing source code, trained models, or derived partition files at this stage.

What we have done is make the manuscript self-sufficient as a specification. The Methods section states the architecture and every dimension, the per-dataset partition axes and client counts, the Dirichlet concentration settings, the windowing and normalisation for each modality, the optimiser, learning rate, batch sizes, local epochs, participation rate and dropout range, the differential-privacy parameters and accountant, and the complete SpectralFedAvg procedure as Algorithm 1. Every metric is defined in closed form. All four datasets are public and cited to their original repositories. Our intent is that a competent reader can reimplement the method and reproduce the protocol from the manuscript alone.

We recognise this is weaker than an artefact release and does not fully meet the reviewer's request. If the editor regards a code release as a condition of acceptance, we would welcome guidance on the required scope and will respond accordingly.

Comment 1.4 — Absence of a formal convergence analysis

R1: The theoretical analysis provides a truncation-error bound for SpectralFedAvg, but a formal convergence analysis under non-IID federated settings is not provided. The authors themselves acknowledge this limitation.

Response: We have strengthened this from a bare acknowledgement into a substantive position, while stopping short of claiming a result we have not proved.

Remark 2 now argues as follows. SpectralFedAvg departs from FedAvg in exactly two places: the coefficient block is averaged after per-edge truncation, and the noise on that block is order-scaled by a factor at most one. Neither modification changes the expectation of the update once truncation error is accounted for, so the algorithm fits the standard template of a biased-gradient FedAvg variant. Existing non-IID analyses therefore apply with an additive per-round perturbation bounded by the new layer-accumulation inequality, which by Proposition 1 decays exponentially in the consensus degree. At our operating point the perturbation is on the order of 4e-3, roughly an order of magnitude below the per-round DP noise.

What remains genuinely open — and we now say so explicitly rather than gesturing at "future work" — is a self-contained proof tracking the interaction between the round-varying consensus degree and client drift, instead of treating truncation as an exogenous perturbation. We would rather state that limitation precisely than overclaim a convergence guarantee.

Comment 1.5 — Novelty claims stated as "to our knowledge"

R1: Several novelty claims rely on phrases such as "to our knowledge." These claims should be stated more cautiously and supported by a stronger comparison with the most recent literature.

Response: We have reduced these claims from eight occurrences to two, retaining the hedge only where the scope is narrow and checkable (the MIMII protocol and the joint four-benchmark evaluation) and removing it entirely elsewhere. In particular, the framework is no longer described as "the first federated framework"; it is now described simply as a federated framework, with the contribution argued on its merits rather than on primacy.

On literature coverage, we have added a new "Federated time-series modelling" subsection covering FeD-TST and FedST, and extended Table 1 accordingly, so that positioning rests on an explicit comparison rather than on the absence of a counterexample.

This exercise also caused us to correct a claim. In re-examining the recent literature we identified FedMamba-IoMT (PLoS ONE 21(8): e0355601), which federates stacked selective-SSM blocks with DP-SGD and Renyi accounting for medical-IoT intrusion detection. Our submitted text described a federated Mamba backbone as itself distinguishing, which that work shows is not accurate. We have corrected both the Introduction and the Related Work positioning to cite it, to state that federated Mamba is already established, and to narrow our claim to what it should have been: the pairing of that backbone with a structured KAN head aggregated by a basis-aware rule rather than by ordinary weight averaging. We are grateful the comment prompted the check.

Comment 1.6 — Simulated setting; no real-world deployment evaluation

R1: The experiments are conducted in a simulated federated environment. Real-world deployment aspects such as latency, energy consumption, memory requirements, and edge-device performance were not evaluated, which limits the practical validation of the proposed method.

Response: This is a real limitation and we have not tried to argue it away. We do not have hardware-in-the-loop measurements, and we decline to convert parameter counts into performance claims we did not measure.

The Limitations section now separates clearly what the architecture does establish from what it does not. Established: a static budget of roughly 1.3 M parameters and 312 M MACs per inference window, giving approximately 5.2 MB of weights at float32 and 1.3 MB at int8, plus a per-round upload of 5.3 MB per participating client, which is the quantity that would govern connectivity cost in a real fleet. These place the model inside the static envelope of Raspberry Pi-class devices and outside that of Cortex-M/STM32-class microcontrollers, which cannot hold the weights at any precision we evaluated.

Not established, and now stated as such: that inference meets any particular latency target, that on-device training is energy-feasible under a duty cycle, or that thermal behaviour is acceptable under sustained load. We now write that the practical deployability of the method should be regarded as plausible but unproven until such a study is run, and we identify this as the single most important gap between these results and industrial adoption.

Comment 1.7 — Information leakage through consensus-degree transmission

R1: The privacy analysis is interesting, but the discussion admits a potential information-leakage issue through consensus-degree transmission. This aspect deserves further investigation and stronger justification.

Response: We have replaced the previous one-sentence admission with a quantified analysis and three costed mitigations.

What leaks: each consensus degree is an integer in {0, ..., 8}, so the entire per-round side channel from a client is a vector of d_in x d_out symbols over an alphabet of size nine, carrying at most about 3.17 bits per edge before accounting for strong inter-edge correlation. Semantically it reveals the smoothness of the fitted edge functions — how quickly the local Chebyshev spectrum decays — not the coefficients and not any sample. The realistic threat is therefore attribute inference about operating regime, not reconstruction of training data.

Three mitigations are now stated with their costs. (i) Releasing only the maximum through a secure-aggregation enclave closes the channel exactly, at the cost of one secure-max protocol per edge. (ii) Releasing a discrete-Laplace-noised histogram costs an additive epsilon_N by basic composition; at epsilon_N = 0.5 this moves the reported budget from 8.5 to 9.0, which does not change the privacy regime the method occupies. (iii) Fixing the degree removes the channel entirely and reduces the method to plain coefficient averaging, at a measured cost of 1.0 pp on CWRU (Table 7).

Option (iii) gives the honest upper bound on what the side channel is worth: an adversary observing the degrees gains at most the information that purchasing 1.0 pp of accuracy costs. We recommend option (ii) for deployment and report results without it, so that the accuracy figures remain attributable to the aggregation rule alone. A joint accountant composing the coefficient channel and the degree histogram, rather than combining them by basic composition, remains future work.

Reviewer #2 (Prof. Ashraf A. Abu-Ein)

Comment 2.1 — Figure/table mismatches and label readability

R2: Adjust Fig 4: captions say CWRU 94.0%, Paderborn 91.0% — Table 3 says 95.0% / 92.0%. This is a mismatch. And Fig 3: MIMII panel has a text label bleeding into the Local-only bar area — check readability.

Response: Both are corrected, and we went further to prevent recurrence.

Fig 4 has been regenerated so that the confusion-matrix counts sum exactly to the accuracies in Table 3 (CWRU 1140/1200 = 95.0%; Paderborn 828/900 = 92.0%), and the caption now states that entries are pooled at the mean operating point over the eight seeds and names the table they match.

Fig 3 has been regenerated from the Table 3 and Table 4 values directly. The stray annotation overlapping the Local-only bar in the MIMII panel is removed, and the gain sub-panels are now computed from the tabulated values rather than drawn separately.

We also audited every other figure against its source table and found two further inconsistencies the reviewers had not flagged: Fig 10 (ablation) did not match Table 7, and Fig 8 (communication) plotted accuracies from an earlier configuration. Both are regenerated directly from the tabulated values. A duplicated figure file was removed, and the overlapping tick labels at the low-alpha end of Fig 7 were resolved. Every figure in the revised manuscript has now been checked cell-by-cell against the table it summarises.

Comment 2.2 — DP notation conflates weight-space and spectral noise

R2: The notation for the sampled Gaussian mechanism in the coordinator step (line 21) conflates weight-space DP with the spectral DP. It would be clearer to separate the two noise-addition steps explicitly.

Response: This was a fair criticism of the presentation, and addressing it also let us state the accounting more rigorously than before.

Algorithm 1 is restructured into two clearly delimited stages: a noise-free aggregation stage (Steps 1-2) and a DP release stage (Steps 3a and 3b) with the weight block and the spectral block handled on separate, labelled lines. The interleaving that caused the confusion — spectral noise inside the edge loop, weight noise afterwards — is gone.

The accompanying prose now makes the structure explicit. Each client uploads a single vector formed by concatenating two disjoint parameter blocks; clipping is applied once, to the concatenation, so the joint per-round L2 sensitivity of the whole release is S. Because the blocks are disjoint coordinates of one vector and the spectral scaling factor is at most one, the two additions are not two mechanisms composed in sequence but one Gaussian mechanism whose per-coordinate scale is at most sigma*S. Its privacy cost is therefore no larger than the isotropic Gaussian mechanism at the same parameters, no budget-splitting factor is required, and the RDP composition applies unmodified. We also now state explicitly what the reported epsilon does not cover — the consensus degrees, which are transmitted outside the vector — and cross-reference the analysis of that channel.

Comment 2.3 — Add the new Chebyshev-KAN re-run

R2: Add the new re-run of the Chebyshev-KAN model; the column-by-column ordering of methods and the qualitative ablation.

Response: As explained under Comment 1.1, the reported table values were already those of the final Chebyshev-KAN model; the sentence suggesting otherwise was an editing error and has been removed, and the Results section now states the provenance of the numbers explicitly. The reviewer was nonetheless right that something had not been updated: four figures were still drawn from the earlier configuration, and these have been regenerated from the final results as detailed under Comment 2.1.

Comment 2.4 — Protocol-incompatible comparisons in the results tables

R2: The authors admit FedSNGP uses an "open-set label shift combined with sample-quantity heterogeneity" protocol, while this work uses Dirichlet partitioning. Such comparisons should be moved to a "contextual discussion" paragraph and removed from the primary results table, or the table should clearly flag incompatible protocols.

Response: We adopted the reviewer's first option. All externally published values are removed from Tables 3 and 4 — FedSNGP and VibrMamba from Table 3, and the MIMII autoencoder, LSTM, and ABGRU rows from Table 4. Both tables now contain only methods run under our own architecture, partition protocol, and seed budget, so every row is like-for-like and the captions say so.

A new "Contextual comparison with externally published results" paragraph in the Discussion houses these numbers with their protocol caveats: the scenario definitions behind FedSNGP's figures, the fully centralised setting of VibrMamba, the per-machine-ID protocol of the MIMII baseline, and the single-seed centralised conventions of the C-MAPSS baselines. We think this is the right structure: the numbers remain available to readers as context, but they no longer sit in a column that invites a direct comparison they cannot support. The paragraph also points to the Methods section, where our partitioning procedure is specified in enough detail for these methods to be re-run under the present protocol.

Comment 2.5 — Does C_r absorb the layer-accumulation factor?

R2: Clarify whether C_r absorbs this factor or if the bound in Eq. (10) already includes it.

Response: It does not, and the previous wording was ambiguous on precisely this point. Remark 2 now states outright that C_r is the universal Jackson constant for a single univariate function on [-1,1] and absorbs no layer-accumulation factor, and that Eq. (10) is a strictly per-edge statement.

We have added the layer-level bound as a separate numbered equation: each output coordinate sums d_in independent per-edge errors, so by Cauchy-Schwarz the layer-output error is bounded by sqrt(d_in) times the per-edge bound, contributing a factor of 8 for our architecture. The remark closes by directing readers to apply the new equation, not Eq. (10), at layer granularity.

Comment 2.6 — The Introduction is too long

R2: The Introduction is strong but overly long (3 pages). The three "architectural opportunities" could be condensed.

Response: The three-opportunities paragraph has been condensed by roughly 36% (3,781 to 2,426 characters) and restructured with bold signposts — "First, the backbone", "Second, the head", "Third, privacy" — so the three-part structure is visible at a glance rather than requiring the reader to track it through continuous prose. Supporting detail that duplicated the Related Work section (VibrMamba accuracy figures, the Kolmogorov-Arnold representation theorem, the CG-FKAN discussion) has been removed from the Introduction, since it appears there in fuller form.

Comment 2.7 — Table 1 omits recent federated time-series works

R2: The Related Work table (Table 1) is useful but omits recent federated time-series works (e.g., Fedformer, Fed-TST) that are relevant to the IIoT scope.

Response: We have added FeD-TST and FedST to Table 1 and written a new "Federated time-series modelling" subsection covering both: FeD-TST federates a temporal sparse transformer for QoS forecasting across IoT network configurations, and FedST provides a secure federated shapelet-transformation protocol for time-series classification. Both establish that sequence models can be federated without exposing raw traces, and neither addresses basis-parameterised heads, which sharpens our positioning.

One respectful clarification on FEDformer. Despite the name, it is not a federated method: the prefix abbreviates "Frequency Enhanced Decomposed", and the model is centralised. We mention this not to dismiss the suggestion but because we suspect the naming causes this confusion frequently, and it seemed more useful to flag it in the paper than to omit the work silently. We have therefore included FEDformer in Table 1 with an explicit footnote explaining the naming, because it is in fact the closest conceptual neighbour to SpectralFedAvg outside FL — it likewise exploits signal sparsity in a fixed frequency basis. The distinction we now draw is that FEDformer uses the frequency domain to compress attention within one model, whereas SpectralFedAvg uses a fixed orthogonal basis to make coefficient averaging across clients well posed, which is a federated-aggregation problem with no centralised analogue. We are grateful for the suggestion, since it prompted a comparison that strengthens the motivation for the fixed basis.

Comment 2.8 — The LSTM comparison is uninformative

R2: Compare federated methods against each other and against the centralized upper bound of the same architecture. The LSTM comparison is uninformative.

Response: We agree, and on reflection we think this comparison was not merely uninformative but mildly misleading, so we have removed it entirely rather than merely de-emphasising it.

The claim that our federated model surpassed the centralised LSTM baseline is deleted from the Abstract, the Results, and the Conclusion. The C-MAPSS results paragraph now compares against the federated baselines trained on the identical architecture, partition, and seed budget (reductions of 1.7, 0.6, and 0.8 RMSE units against FedAvg, SCAFFOLD, and FedBN respectively), and against the centralised run of the same architecture at RMSE 12.5, which is the only valid ceiling. The residual 2.6-unit gap is thereby attributable to the federated constraint alone, with capacity held fixed.

We also state the underlying reasoning in the Discussion: a federated model appearing to beat a centralised one is far more likely to reflect differing preprocessing conventions than genuine superiority, and presenting it as an achievement was not sound. We thank the reviewer for pressing on this.

We hope these revisions address the reviewers' concerns. We are particularly grateful to Reviewer 1 for catching the pending-re-run sentence, which materially misrepresented the state of the work, and to Reviewer 2 for the comparison-validity and DP-notation points, both of which corrected genuine weaknesses in how the results were framed.

Sincerely,

Mohammed Tawfik, on behalf of all co-authors

Corresponding author

Attachment Submitted filename: Response_to_Reviewers.pdf

# Peer review: Decision Letter 1

**DOI:** 10.1371/journal.pone.0354012.r003

**Authors:** Sohail Saif

7 Sep 2026

PONE-D-26-32102R1 FedMamba-ChebKAN: Federated Mamba with Chebyshev-KAN for Heterogeneous IIoT Fault Detection PLOS One

Dear Dr. Tawfik,

Thank you for submitting your manuscript to PLOS One. After careful consideration, we feel that it has merit but does not fully meet PLOS One’s publication criteria as it currently stands. Therefore, we invite you to submit a revised version of the manuscript that addresses the points raised during the review process.

Please submit your revised manuscript by Oct 07 2026 11:59PM. If you will need more time than this to complete your revisions, please reply to this message or contact the journal office at plosone@plos.org. When you're ready to submit your revision, log on to https://www.editorialmanager.com/pone/ and select the 'Submissions Needing Revision' folder to locate your manuscript file.

Please include the following items when submitting your revised manuscript:

- A letter that responds to each point raised by the academic editor and reviewer(s). You should upload this letter as a separate file labeled 'Response to Reviewers'.
- A marked-up copy of your manuscript that highlights changes made to the original version. You should upload this as a separate file labeled 'Revised Manuscript with Track Changes'.
- An unmarked version of your revised paper without tracked changes. You should upload this as a separate file labeled 'Manuscript'.

If you would like to make changes to your financial disclosure, please include your updated statement in your cover letter. Guidelines for resubmitting your figure files are available below the reviewer comments at the end of this letter.

If applicable, we recommend that you deposit your laboratory protocols in protocols.io to enhance the reproducibility of your results. Protocols.io assigns your protocol its own identifier (DOI) so that it can be cited independently in the future. For instructions see: https://journals.plos.org/plosone/s/submission-guidelines#loc-laboratory-protocols. Additionally, PLOS One offers an option for publishing peer-reviewed Lab Protocol articles, which describe protocols hosted on protocols.io. Read more information on sharing protocols at https://plos.org/protocols?utm_medium=editorial-email&utm_source=authorletters&utm_campaign=protocols.

As the corresponding author, your ORCID iD is verified in the submission system and will appear in the published article. PLOS supports the use of ORCID, and we encourage all coauthors to register for an ORCID iD and use it as well. Please encourage your coauthors to verify their ORCID iD within the submission system before final acceptance, as unverified ORCID iDs will not appear in the published article. *Only* the individual author can complete the verification step; PLOS staff *cannot* verify ORCID iDs on behalf of authors.

We look forward to receiving your revised manuscript.

Kind regards,

Sohail Saif, Ph.D

Academic Editor

PLOS One

Journal Requirements:

If the reviewer comments include a recommendation to cite specific previously published works, please review and evaluate these publications to determine whether they are relevant and should be cited. There is no requirement to cite these works unless the editor has indicated otherwise.

Please review your reference list to ensure that it is complete and correct. If you have cited papers that have been retracted, please include the rationale for doing so in the manuscript text, or remove these references and replace them with relevant current references. Any changes to the reference list should be mentioned in the rebuttal letter that accompanies your revised manuscript. If you need to cite a retracted article, indicate the article’s retracted status in the References list and also include a citation and full reference for the retraction notice.

[Note: HTML markup is below. Please do not edit.]

Reviewers' comments:

Reviewer's Responses to Questions

**Comments to the Author**

1. If the authors have adequately addressed your comments raised in a previous round of review and you feel that this manuscript is now acceptable for publication, you may indicate that here to bypass the “Comments to the Author” section, enter your conflict of interest statement in the “Confidential to Editor” section, and submit your "Accept" recommendation.

Reviewer #1: All comments have been addressed

Reviewer #2: All comments have been addressed

**********

2. Is the manuscript technically sound, and do the data support the conclusions?

The manuscript must describe a technically sound piece of scientific research with data that supports the conclusions. Experiments must have been conducted rigorously, with appropriate controls, replication, and sample sizes. The conclusions must be drawn appropriately based on the data presented.

Reviewer #1: Partly

Reviewer #2: (No Response)

**********

3. Has the statistical analysis been performed appropriately and rigorously?

Reviewer #1: Yes

Reviewer #2: Yes

**********

4. Have the authors made all data underlying the findings in their manuscript fully available?

The PLOS Data policy requires authors to make all data underlying the findings described in their manuscript fully available without restriction, with rare exception (please refer to the Data Availability Statement in the manuscript PDF file). The data should be provided as part of the manuscript or its supporting information, or deposited to a public repository. For example, in addition to summary statistics, the data points behind means, medians and variance measures should be available. If there are restrictions on publicly sharing data—e.g. participant privacy or use of data from a third party—those must be specified.

Reviewer #1: No

Reviewer #2: Yes

**********

5. Is the manuscript presented in an intelligible fashion and written in standard English?

PLOS ONE does not copyedit accepted manuscripts, so the language in submitted articles must be clear, correct, and unambiguous. Any typographical or grammatical errors should be corrected at revision, so please note any specific errors here.

Reviewer #1: Yes

Reviewer #2: Yes

**********

6. Review Comments to the Author

Please use the space provided to explain your answers to the questions above. You may also include additional comments for the author, including concerns about dual publication, research ethics, or publication ethics. (Please upload your review as an attachment if it exceeds 20,000 characters)

Reviewer #1: ors have made a serious effort to address the concerns raised during the previous review round. The revised manuscript is substantially improved in terms of methodological clarity, statistical reporting, literature positioning, figure consistency, and discussion of limitations. In particular, the previously concerning statement regarding pending experimental validation has been removed and replaced with a clear description of the provenance of the reported results. The figures, tables, and comparative analyses have also been revised and aligned more consistently with the reported findings.

The statistical analysis appears appropriately documented, with multiple independent runs, Wilcoxon signed-rank testing, effect-size reporting, and clearly defined evaluation metrics. The discussion of privacy guarantees, convergence limitations, and deployment constraints has been strengthened and is presented in a balanced manner.

One issue remains regarding reproducibility and data availability. Although all benchmark datasets are publicly accessible, the manuscript does not provide the underlying experimental outputs, source code, trained models, or partition files used to generate the reported results. Therefore, full availability of all data underlying the findings is not yet achieved according to the strictest interpretation of reproducibility requirements.

Overall, the authors have satisfactorily addressed the major reviewer comments, and the remaining concerns are primarily related to reproducibility rather than scientific validity. The manuscript is well written, technically sound, and significantly improved from the previous version.

Reviewer #2: -The authors have done an excellent job addressing the concerns raised in the previous round of review. The revised manuscript, "FedMamba-ChebKAN: Federated Mamba with Chebyshev-KAN for Heterogeneous IIoT Fault Detection," is significantly improved.

-I recommend this manuscript for Acceptance. Below are a few minor, simple comments for the authors to consider for final polishing before publication.

Please do a final visual check of Figure 7 and Figure 10 to ensure all axis labels, legends, and annotations are perfectly legible in the final high-resolution export.

-in the Abstract, ensure consistent spacing (e.g., "p rovidin g" and "freque ncy" appear to have minor spacing artifacts in the provided text draft, likely from PDF extraction

**********

7. PLOS authors have the option to publish the peer review history of their article (what does this mean?). If published, this will include your full peer review and any attached files.

If you choose “no”, your identity will remain anonymous but your review may still be made public.

**Do you want your identity to be public for this peer review?** For information about this choice, including consent withdrawal, please see our Privacy Policy.

Reviewer #1: **Yes:** Dr Muhammad Ali Abid

Reviewer #2: No

**********

[NOTE: If reviewer comments were submitted as an attachment file, they will be attached to this email and accessible via the submission site. Please log into your account, locate the manuscript record, and check for the action link "View Attachments". If this link does not appear, there are no attachment files.]

To ensure your figures meet our technical requirements, please review our figure guidelines: https://journals.plos.org/plosone/s/figures

You may also use PLOS’s free figure tool, NAAS, to help you prepare publication quality figures: https://journals.plos.org/plosone/s/figures#loc-tools-for-figure-preparation.

NAAS will assess whether your figures meet our technical requirements by comparing each figure against our figure specifications.

# Author comment: Author response to Decision Letter 2

**DOI:** 10.1371/journal.pone.0354012.r004

8 Sep 2026

Response to Reviewers

Manuscript PONE-D-26-32102R1

FedMamba-ChebKAN: Federated Mamba with Chebyshev-KAN for Heterogeneous IIoT Fault Detection

Dear Dr. Saif,

Thank you for the opportunity to submit a further revision, and please pass our thanks to both reviewers for their careful reading of the R1 manuscript and for confirming that all previous comments have been addressed. This revision responds to the three remaining points: (1) Reviewer 1's remark on data and code availability, (2) the legibility of Figures 7 and 10, and (3) the spacing artefacts noted in the Abstract. Every change is marked in the tracked-changes copy; the reviewer text is reproduced below in full, followed by our response and the corresponding change.

Reviewer #1

Comment 1.1 “The authors have made a serious effort to address the concerns raised during the previous review round. The revised manuscript is substantially improved in terms of methodological clarity, statistical reporting, literature positioning, figure consistency, and discussion of limitations. […] The statistical analysis appears appropriately documented, with multiple independent runs, Wilcoxon signed-rank testing, effect-size reporting, and clearly defined evaluation metrics. The discussion of privacy guarantees, convergence limitations, and deployment constraints has been strengthened and is presented in a balanced manner.”

Response We thank the reviewer for this assessment. No change was required in response to this comment.

Comment 1.2 “One issue remains regarding reproducibility and data availability. Although all benchmark datasets are publicly accessible, the manuscript does not provide the underlying experimental outputs, source code, trained models, or partition files used to generate the reported results. Therefore, full availability of all data underlying the findings is not yet achieved according to the strictest interpretation of reproducibility requirements.”

Response We thank the reviewer for distinguishing this point from the scientific validity of the work. All four benchmark datasets (CWRU, Paderborn, MIMII, NASA C-MAPSS) are publicly available from their original repositories, and the Data Availability statement gives the exact download locations; no new data were generated in this study, so all data underlying the findings are fully accessible without restriction.

With respect to experimental outputs, source code, trained models, and partition files: the manuscript already contains everything required to reproduce the reported results independently. The architecture is fully specified (Methods), the aggregation rule is given as Algorithm 1 with its DP mechanism, every evaluation metric is defined in closed form, the complete hyperparameter set is listed in the Experimental setup (T = 200, E = 2, C = 0.3, d_m = 64, N_layers = 3, d_state = 16, d_conv = 4, p = 8, KAN dims [64,32], N = 8, τ = 10⁻³, σ = S = γ = 1.0, K = 12/20), the non-IID partition protocol is described in the Datasets section together with the Dirichlet α levels, and the number of seeds, the statistical tests, and the effect-size measure are all stated. We therefore respectfully maintain the current data statement, which we believe satisfies the PLOS data policy for a study based entirely on public benchmark datasets. We are, of course, happy to respond to specific reproducibility questions from the editor.

Changes: none to the manuscript for this point.

Reviewer #2

Comment 2.1 “The authors have done an excellent job addressing the concerns raised in the previous round of review. The revised manuscript […] is significantly improved. I recommend this manuscript for Acceptance. Below are a few minor, simple comments for the authors to consider for final polishing before publication.”

Response We are grateful for the recommendation and have addressed both polishing items below.

Comment 2.2 “Please do a final visual check of Figure 7 and Figure 10 to ensure all axis labels, legends, and annotations are perfectly legible in the final high-resolution export.”

Response We inspected both figures at full resolution and found two genuine legibility problems, which are now fixed.

Figure 7 (non-IID robustness). The horizontal-axis tick labels for α = 0.05 and α = 0.10 overlapped (the “(extreme)”/“(severe)” descriptors collided with the “(mod.)” label of α = 0.30). The tick labels have been re-laid-out on separate lines so that no label overlaps; the plotted data, legend, and Zhou et al. marker are unchanged. The caption was also corrected: it previously referred to “±1σ shaded” bands that the figure does not draw; it now lists the α levels shown on the axis.

Figure 10 (ablation). The six rotated multi-line configuration names on the x-axis of each panel were too small to read, and the C-MAPSS panel used an inverted axis with bars hanging from the top, which several readers found confusing. The figure has been regenerated from Table 7 with (i) short configuration letters A–F on the axis and a full-text key beneath the panels, (ii) conventional upward bars with ±1σ error bars in all three panels, (iii) the numerical value printed above each bar, and (iv) larger, non-overlapping Δ annotations on the CWRU panel. All values are identical to Table 7. The caption now defines configurations A–F explicitly and maps them to the rows of Table 7.

Both figures are exported at ≥300 dpi and have been checked against the PLOS figure specifications.

Comment 2.3 “In the Abstract, ensure consistent spacing (e.g., “p rovidin g” and “freque ncy” appear to have minor spacing artifacts in the provided text draft, likely from PDF extraction).”

Response We checked the LaTeX source and the compiled PDF: the words “providing” and “frequency” contain no stray spaces in the source, and text extracted from our compiled PDF reads correctly. As the reviewer suspects, the artefacts arose from text extraction of the review PDF (character-spacing adjustments from the microtype package, which the PLOS template enables, are occasionally mis-parsed by extractors). To eliminate the possibility that the artefact recurs in the production PDF, we have also disabled microtype expansion/tracking for the Abstract in the revised source. No wording in the Abstract has changed.

Additional changes

Beyond the points above, Figure 1 (system architecture) has been redrawn as a vector figure so that all tier labels, module names, and the three SpectralFedAvg steps are legible at print size; its content is unchanged. While checking the Abstract for the spacing issue we also made a few minor wording refinements in the Abstract and Conclusion (e.g. "integrates" → "couples", "satisfying" → "respecting"); these are stylistic only and visible in the tracked-changes copy. No result, number, or reference was changed in this revision. The reference list is unchanged and contains no retracted articles. The author list and order in Editorial Manager have been checked against the manuscript byline.

We hope the manuscript is now suitable for publication in PLOS ONE and thank the editor and reviewers again for their time.

Yours sincerely,

Mohammed Tawfik (on behalf of all authors)

Attachment Submitted filename: Response_to_Reviewers_R2.pdf

# Peer review: Decision Letter 2

**DOI:** 10.1371/journal.pone.0354012.r005

**Authors:** Sohail Saif

2 Oct 2026

PONE-D-26-32102R2 FedMamba-ChebKAN: Federated Mamba with Chebyshev-KAN for Heterogeneous IIoT Fault Detection PLOS One

Dear Dr. Tawfik,

Thank you for submitting your manuscript to PLOS One. After careful consideration, we feel that it has merit but does not fully meet PLOS One’s publication criteria as it currently stands. Therefore, we invite you to submit a revised version of the manuscript that addresses the points raised during the review process.

Please submit your revised manuscript by Nov 16 2026 11:59PM. If you will need more time than this to complete your revisions, please reply to this message or contact the journal office at plosone@plos.org. When you're ready to submit your revision, log on to https://www.editorialmanager.com/pone/ and select the 'Submissions Needing Revision' folder to locate your manuscript file.

Please include the following items when submitting your revised manuscript:

- A letter that responds to each point raised by the academic editor and reviewer(s). You should upload this letter as a separate file labeled 'Response to Reviewers'.
- A marked-up copy of your manuscript that highlights changes made to the original version. You should upload this as a separate file labeled 'Revised Manuscript with Track Changes'.
- An unmarked version of your revised paper without tracked changes. You should upload this as a separate file labeled 'Manuscript'.

If you would like to make changes to your financial disclosure, please include your updated statement in your cover letter. Guidelines for resubmitting your figure files are available below the reviewer comments at the end of this letter.

If applicable, we recommend that you deposit your laboratory protocols in protocols.io to enhance the reproducibility of your results. Protocols.io assigns your protocol its own identifier (DOI) so that it can be cited independently in the future. For instructions see: https://journals.plos.org/plosone/s/submission-guidelines#loc-laboratory-protocols. Additionally, PLOS One offers an option for publishing peer-reviewed Lab Protocol articles, which describe protocols hosted on protocols.io. Read more information on sharing protocols at https://plos.org/protocols?utm_medium=editorial-email&utm_source=authorletters&utm_campaign=protocols.

As the corresponding author, your ORCID iD is verified in the submission system and will appear in the published article. PLOS supports the use of ORCID, and we encourage all coauthors to register for an ORCID iD and use it as well. Please encourage your coauthors to verify their ORCID iD within the submission system before final acceptance, as unverified ORCID iDs will not appear in the published article. *Only* the individual author can complete the verification step; PLOS staff *cannot* verify ORCID iDs on behalf of authors.

We look forward to receiving your revised manuscript.

Kind regards,

Sohail Saif, Ph.D

Academic Editor

PLOS One

Journal Requirements:

If the reviewer comments include a recommendation to cite specific previously published works, please review and evaluate these publications to determine whether they are relevant and should be cited. There is no requirement to cite these works unless the editor has indicated otherwise.

[Note: HTML markup is below. Please do not edit.]

Reviewers' comments:

Reviewer's Responses to Questions

**Comments to the Author**

1. If the authors have adequately addressed your comments raised in a previous round of review and you feel that this manuscript is now acceptable for publication, you may indicate that here to bypass the “Comments to the Author” section, enter your conflict of interest statement in the “Confidential to Editor” section, and submit your "Accept" recommendation.

Reviewer #1: All comments have been addressed

Reviewer #3: (No Response)

**********

2. Is the manuscript technically sound, and do the data support the conclusions?

The manuscript must describe a technically sound piece of scientific research with data that supports the conclusions. Experiments must have been conducted rigorously, with appropriate controls, replication, and sample sizes. The conclusions must be drawn appropriately based on the data presented.

Reviewer #1: Yes

Reviewer #3: Partly

**********

3. Has the statistical analysis been performed appropriately and rigorously?

Reviewer #1: Yes

Reviewer #3: No

**********

4. Have the authors made all data underlying the findings in their manuscript fully available?

The PLOS Data policy requires authors to make all data underlying the findings described in their manuscript fully available without restriction, with rare exception (please refer to the Data Availability Statement in the manuscript PDF file). The data should be provided as part of the manuscript or its supporting information, or deposited to a public repository. For example, in addition to summary statistics, the data points behind means, medians and variance measures should be available. If there are restrictions on publicly sharing data—e.g. participant privacy or use of data from a third party—those must be specified.

Reviewer #1: No

Reviewer #3: No

**********

5. Is the manuscript presented in an intelligible fashion and written in standard English?

PLOS ONE does not copyedit accepted manuscripts, so the language in submitted articles must be clear, correct, and unambiguous. Any typographical or grammatical errors should be corrected at revision, so please note any specific errors here.

Reviewer #1: Yes

Reviewer #3: No

**********

6. Review Comments to the Author

Please use the space provided to explain your answers to the questions above. You may also include additional comments for the author, including concerns about dual publication, research ethics, or publication ethics. (Please upload your review as an attachment if it exceeds 20,000 characters)

Reviewer #1: The authors have responded comprehensively to the comments raised during the previous review rounds, and the manuscript has improved significantly in terms of clarity, presentation, methodological transparency, statistical reporting, and discussion of limitations. The revisions to the figures, particularly Figures 1, 7, and 10, have improved readability and presentation quality. The clarification of the experimental provenance, differential privacy accounting, convergence discussion, and comparison strategy with external studies has strengthened the scientific rigor of the work.

The statistical analysis appears appropriate and well documented. The use of multiple independent runs (R=8), Wilcoxon signed-rank tests, effect-size reporting, confidence measures, and clearly defined evaluation metrics provides a reasonable level of rigor for assessing performance differences across methods. The authors have also addressed previous concerns regarding methodological consistency and result interpretation.

One remaining issue concerns data availability and reproducibility. Although all benchmark datasets are publicly available and properly referenced, the manuscript does not provide source code, trained models, partition files, or the underlying experimental outputs used to generate the reported results. Therefore, full reproducibility remains limited, and the availability of all data underlying the findings may not fully satisfy the strictest interpretation of open-science and reproducibility standards.

The manuscript appropriately acknowledges its remaining limitations, including the lack of real-world edge-device deployment experiments, the absence of a complete convergence proof for SpectralFedAvg under non-IID settings, and the unresolved consensus-degree privacy side-channel. These limitations are transparently discussed and do not undermine the primary contribution of the work.

Overall, the authors have satisfactorily addressed the reviewer comments from previous rounds. The manuscript is technically sound, clearly written, and presents a meaningful contribution to federated learning for industrial fault diagnosis. Subject to the journal’s policy regarding data and code availability, I believe the manuscript is suitable for publication.

Reviewer #3: The manuscript proposes FedMamba-ChebKAN for heterogeneous IIoT fault detection. The topic is relevant, and the evaluation across four datasets is potentially valuable. However, several issues currently affect the validity of the privacy guarantee, fairness of the comparisons, and reliability of the reported results. I therefore recommend major revision.

1. The differential-privacy guarantee requires fundamental correction. The proposed mechanism assigns less noise to higher-order coefficients, but less noise provides weaker privacy unless a correspondingly smaller sensitivity is established for those coefficients. The text also states that all transmitted updates are jointly clipped, whereas Algorithm 1 appears to clip only the ordinary model update. In addition, the consensus degrees are transmitted without protection, and the coordinator receives client updates before adding noise. The authors should correct the mechanism, clarify the threat model, and recalculate the privacy budget using a validated accountant. Otherwise, the claims of formal differential privacy and protection against an honest-but-curious coordinator should be removed or substantially narrowed.

2. The baseline comparison does not clearly separate the contribution of the model architecture from that of SpectralFedAvg. The main FedAvg result appears to use a 1-D CNN, whereas the proposed method uses Mamba with Cheb-KAN. Therefore, the reported improvement cannot be attributed solely to the aggregation method. FedAvg, FedProx, SCAFFOLD, FedBN, and SpectralFedAvg should be compared using the same Mamba plus Cheb-KAN architecture, client partitions, seeds, and tuning budget.

The recent FedMamba study, “FedMamba: Robust multimodal federated intrusion detection for heterogeneous IoT systems,” should also be discussed. Its published results should not be copied directly because the datasets and tasks differ, but a Mamba plus FedProx baseline should be implemented under the present protocol.

3. The data-splitting procedure may permit leakage. CWRU and Paderborn use overlapping windows, but the manuscript does not state whether source recordings or machine units were separated before window generation. Randomly splitting overlapping windows can place nearly identical samples in training and testing sets and inflate performance. The authors should split recordings, bearing units, or engines first, generate windows afterward, and fit all normalization parameters using training data only. Exact split ratios, sample counts, class distributions, and client assignments should be reported. The relationship between the physical client partitions and the Dirichlet partitions must also be clarified.

4. Several evaluation procedures and numerical results are inconsistent. The manuscript reports Macro-AUROC for C-MAPSS without defining how this classification metric applies to the RUL regression task. It should also clarify whether the PHM08 score is calculated per engine or over individual windows. The main CWRU accuracy is reported as a non-private result, although the text identifies privacy noise as the default setting. FedAvg is also reported to reach a 93% target despite having a lower final accuracy. The bandwidth totals do not clearly follow from the update size, number of participating clients, and training rounds. These inconsistencies should be corrected using the underlying per-seed results.

5. The statistical analysis should be revised. Eight random seeds do not automatically make a Wilcoxon test adequately powered. The manuscript also conducts many tests across datasets, metrics, and baselines without addressing multiple comparisons. The authors should define primary outcomes or apply an appropriate correction. The signs of the rank-biserial correlations should also be checked. Finally, the centralized model should be described as a pooled-data reference, not a guaranteed upper bound.

6. The theoretical proposition does not appear to match the actual aggregation procedure. A local Chebyshev truncation bound does not directly describe the error between the averaged global function and each heterogeneous client function. Client heterogeneity introduces an additional error that is currently omitted. Moreover, zero-padding a shorter coefficient vector does not recover coefficients excluded by local truncation. The authors should either provide a corrected result that covers heterogeneous aggregation or restrict the proposition to local approximation error. The manuscript should not describe this approximation result as an exponential federated-learning convergence rate.

7. The reproducibility materials are insufficient. Public availability of the original datasets does not cover the processed samples, client partitions, random seeds, per-seed outputs, or values underlying the tables and figures. The authors should release the implementation, dependency versions, preprocessing scripts, split and client-assignment files, experiment configurations, privacy-accountant code, and data supporting all reported results. Pseudocode alone is insufficient to reproduce the proposed method.

8. The presentation and interpretation claims should be moderated. The figures are difficult to read and should be regenerated at publication quality with larger labels, consistent fonts, and clear legends. Figure 12 should be cited and explained or removed. Learned Mamba dimensions should not be labelled as specific physical quantities unless the mapping is validated. Similarly, claims of mechanical interpretability, governance compliance, formal privacy, and being the “first” study should be retained only when directly supported. Promotional expressions should be replaced with neutral scientific language, and the Abstract should be reduced to the journal’s 300-word limit.

**********

7. PLOS authors have the option to publish the peer review history of their article (what does this mean?). If published, this will include your full peer review and any attached files.

If you choose “no”, your identity will remain anonymous but your review may still be made public.

**Do you want your identity to be public for this peer review?** For information about this choice, including consent withdrawal, please see our Privacy Policy.

Reviewer #1: **Yes:** Dr Muhammad Ali Abid

Reviewer #3: No

**********

[NOTE: If reviewer comments were submitted as an attachment file, they will be attached to this email and accessible via the submission site. Please log into your account, locate the manuscript record, and check for the action link "View Attachments". If this link does not appear, there are no attachment files.]

To ensure your figures meet our technical requirements, please review our figure guidelines: https://journals.plos.org/plosone/s/figures

You may also use PLOS’s free figure tool, NAAS, to help you prepare publication quality figures: https://journals.plos.org/plosone/s/figures#loc-tools-for-figure-preparation.

NAAS will assess whether your figures meet our technical requirements by comparing each figure against our figure specifications.

# Author comment: Author response to Decision Letter 3

**DOI:** 10.1371/journal.pone.0354012.r006

8 Oct 2026

Response to Reviewers in Related file

Attachment Submitted filename: Response_to_Reviewers_R3.pdf

# Peer review: Decision Letter 3

**DOI:** 10.1371/journal.pone.0354012.r007

**Authors:** Sohail Saif

26 Oct 2026

FedMamba-ChebKAN: Federated Mamba with Chebyshev-KAN for Heterogeneous IIoT Fault Detection

PONE-D-26-32102R3

Dear Dr. Tawfik,

We’re pleased to inform you that your manuscript has been judged scientifically suitable for publication and will be formally accepted for publication once it meets all outstanding technical requirements.

Within one week, you’ll receive an e-mail detailing the required amendments. When these have been addressed, you’ll receive a formal acceptance letter and your manuscript will be scheduled for publication.

An invoice will be generated when your article is formally accepted. Please note, if your institution has a publishing partnership with PLOS and your article meets the relevant criteria, all or part of your publication costs will be covered. Please make sure your user information is up-to-date by logging into Editorial Manager at Editorial Manager® and clicking the ‘Update My Information' link at the top of the page. For questions related to billing, please contact billing support.

If your institution or institutions have a press office, please notify them about your upcoming paper to help maximize its impact. If they’ll be preparing press materials, please inform our press team as soon as possible -- no later than 48 hours after receiving the formal acceptance. Your manuscript will remain under strict press embargo until 2 pm Eastern Time on the date of publication. For more information, please contact onepress@plos.org.

Kind regards,

Sohail Saif, Ph.D

Academic Editor

PLOS One

Additional Editor Comments (optional):

Reviewers' comments:

Reviewer's Responses to Questions

**Comments to the Author**

1. If the authors have adequately addressed your comments raised in a previous round of review and you feel that this manuscript is now acceptable for publication, you may indicate that here to bypass the “Comments to the Author” section, enter your conflict of interest statement in the “Confidential to Editor” section, and submit your "Accept" recommendation.

Reviewer #3: (No Response)

**********

2. Is the manuscript technically sound, and do the data support the conclusions?

The manuscript must describe a technically sound piece of scientific research with data that supports the conclusions. Experiments must have been conducted rigorously, with appropriate controls, replication, and sample sizes. The conclusions must be drawn appropriately based on the data presented.

Reviewer #3: (No Response)

**********

3. Has the statistical analysis been performed appropriately and rigorously?

Reviewer #3: (No Response)

**********

4. Have the authors made all data underlying the findings in their manuscript fully available?

The PLOS Data policy requires authors to make all data underlying the findings described in their manuscript fully available without restriction, with rare exception (please refer to the Data Availability Statement in the manuscript PDF file). The data should be provided as part of the manuscript or its supporting information, or deposited to a public repository. For example, in addition to summary statistics, the data points behind means, medians and variance measures should be available. If there are restrictions on publicly sharing data—e.g. participant privacy or use of data from a third party—those must be specified.

Reviewer #3: (No Response)

**********

5. Is the manuscript presented in an intelligible fashion and written in standard English?

PLOS ONE does not copyedit accepted manuscripts, so the language in submitted articles must be clear, correct, and unambiguous. Any typographical or grammatical errors should be corrected at revision, so please note any specific errors here.

Reviewer #3: (No Response)

**********

6. Review Comments to the Author

Please use the space provided to explain your answers to the questions above. You may also include additional comments for the author, including concerns about dual publication, research ethics, or publication ethics. (Please upload your review as an attachment if it exceeds 20,000 characters)

Reviewer #3: (No Response)

**********

7. PLOS authors have the option to publish the peer review history of their article (what does this mean?). If published, this will include your full peer review and any attached files.

If you choose “no”, your identity will remain anonymous but your review may still be made public.

**Do you want your identity to be public for this peer review?** For information about this choice, including consent withdrawal, please see our Privacy Policy.

Reviewer #3: No

**********

# Editor report: Acceptance letter

**DOI:** 10.1371/journal.pone.0354012.r008

**Authors:** Sohail Saif

PONE-D-26-32102R3

PLOS One

Dear Dr. Tawfik,

I'm pleased to inform you that your manuscript has been deemed suitable for publication in PLOS One. Congratulations! Your manuscript is now being handed over to our production team.

At this stage, our production department will prepare your paper for publication. This includes ensuring the following:

* All references, tables, and figures are properly cited

* All relevant supporting information is included in the manuscript submission,

* There are no issues that prevent the paper from being properly typeset

You will receive further instructions from the production team, including instructions on how to review your proof when it is ready. Please keep in mind that we are working through a large volume of accepted articles, so please give us a few days to review your paper and let you know the next and final steps.

Lastly, if your institution or institutions have a press office, please let them know about your upcoming paper now to help maximize its impact. If they'll be preparing press materials, please inform our press team within the next 48 hours. Your manuscript will remain under strict press embargo until 2 pm Eastern Time on the date of publication. For more information, please contact onepress@plos.org.

You will receive an invoice from PLOS for your publication fee after your manuscript has reached the completed accept phase. If you receive an email requesting payment before acceptance or for any other service, this may be a phishing scheme. Learn how to identify phishing emails and protect your accounts at https://explore.plos.org/phishing.

If we can help with anything else, please email us at customercare@plos.org.

Thank you for submitting your work to PLOS One and supporting open access.

Kind regards,

PLOS One Editorial Office Staff

on behalf of

Dr. Sohail Saif

Academic Editor

PLOS One
